# Synthesis and characterization of 2-(anthracene-9-yl)-4,5-diphenyl-1*H*-imidazole derivatives as environmentally sensitive fluorophores†

**DOI:** 10.1039/d4ra03735a

**Published:** 2024-07-26

**Authors:** Eyad A. Younes, Amneh M. AL-Snaid, Kayed A. Abu-Safieh, Fatemeh Salami, Nayyef Aljaar, Yuming Zhao

**Affiliations:** a Department of Chemistry, Faculty of Science, The Hashemite University PO Box 330127 Zarqa 13133 Jordan e.younes@hu.edu.jo +962 (5) 3903333 ext. 4572; b Department of Chemistry, Memorial University of Newfoundland St. John's NL Canada A1C 5S7

## Abstract

2-(Anthracene-9-yl)-4,5-diphenyl-1*H*-imidazole (ADPI) provides an intriguing molecular platform for developing organic fluorophores with diverse properties and fluorescence performances. However, derivatives of ADPI have not yet been well explored and extensive studies are warranted. To shed more light on this, we have synthesized a series of π-extended ADPIs through a concise synthetic route involving an efficient cross-condensation reaction followed by Pd-catalyzed Suzuki cross-coupling. The obtained compounds were subjected to X-ray single crystallographic analysis to understand their molecular conformational and solid-state packing properties. Furthermore, UV-Vis absorption and fluorescence spectroscopic analyses were conducted. Our experimental results have disclosed interesting solvatofluorochromic properties of these compounds which are useful for solvent polarity-sensitive applications. The presence of an amphoteric imidazolyl group in the ADPI derivatives also renders them sensitive fluorescence responses to strong protic acids (*e.g.*, trifluoroacetic acid) as well as fluoride anion. It transpires that the fluorescence changes are dependent on the functional groups attached to the ADPI core, suggesting a bottom-up molecular tuning approach for development of fluorophores and chemosensors with diverse functions.

## Abstract 2

A series of π-extended 2-(anthracene-9-yl)-4,5-diphenyl-1*H*-imidazole (ADPI) derivatives were synthesized. These compounds show sensitive fluorescence responses to solvent polarity, acidity, and fluoride anion.

## Graphical abstract

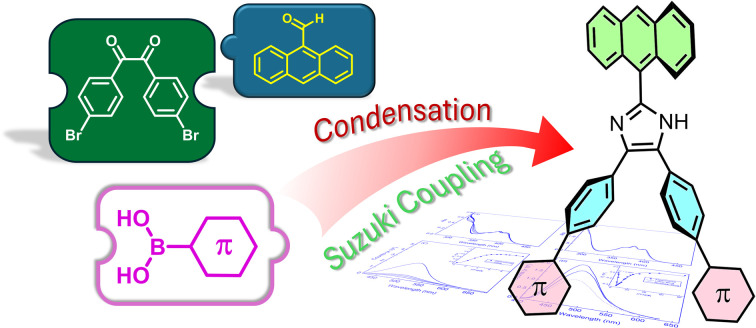

## 1 Introduction

**Fig. 1:**
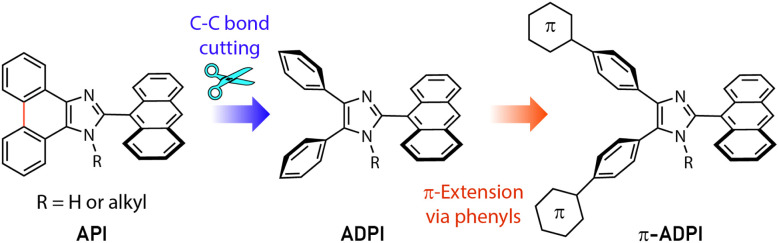
The design strategy for derivatizing the π-extended ADPI (π-ADPI) system in this work.

Imidazole is an important five-membered heterocycle that is widely present in natural products,^1^ biomolecules,^2^ pharmaceutical drugs,^3–6^ synthetic polymers,^7^ selective ligands,^8^ functional organic chromophores,^9,10^ and to just name a few. Over the past decade, planar imidazole-fused polycyclic aromatic systems, such as phenanthroimidazole^11–15^ and pyrenoimidazole derivatives,^16–19^ have captured considerable attention in the field of organic optoelectronics primarily because of their excellent electroluminescence performances and thermal stability. Changing their fused π-motif into non-fused polyaryl-substitution results in increased steric clashing among the aryl groups and force the molecular structures to take more twisted conformations (such as that illustrated in Fig. 1). This type of variation has been found to be beneficial for bringing about new optoelectronic performances that are very different from their planar counterparts. For example, Alreja and Kaur recently reported that 2-(anthracene-9-yl)-4,5-diphenyl-1*H*-imidazole (ADPI, see Fig. 1) shows more selective colorimetric and fluorescence sensing properties for certain transition metal ions (*e.g.*, Cu^2+^) than an analogous fluorophore that contains a planar imidazole-fused phenanthroline.^20^ Li *et al.* in 2014 discovered that ADPI exhibits intriguing polymorphism-dependent fluorescence and piezochromic behavior in the solid state.^21^ Pan and co-workers reported that ADPI molecules are packed in a non-parallel fashion in the crystalline state, yielding single-crystalline microwires that can act as efficient optical waveguides.^22^

Besides the above-mentioned features, ADPI and its numerous analogues have also been investigated in terms of optical absorption/emission properties,^23^ solvatofluorochromism,^24^ electroluminescence,^25^ and catalytic effects on Pd-catalyzed coupling reactions^26^ over the past decade. Nonetheless, the number of ADPI derivatives so far documented in the literature is still much less than many other imidazole-based π-conjugated systems. Continued synthesis and characterizations of new variants of ADPI are therefore warranted. In this work, we designed a series of π-ADPIs that carry various aryl substituents at the *para*-positions of the ADPI's two phenyl groups (see Fig. 1). Synthetically, these π-ADPIs can be flexibly prepared through efficient transition metal-catalyzed cross-coupling reactions, especially the well-established Suzuki-Miyaura cross coupling.^27^ Fundamentally, it is important to acquire understanding about how the aryl substituents influence the structural, electronic, and photophysical properties. Disclosure of relevant substituent–property correlations will offer useful guidance to further exploration of ADPI-based materials in optoelectronic applications. The following presents our synthesis and systematic characterizations of four π-ADPI derivatives based on single-crystal X-ray diffraction (SCXRD), NMR, UV-Vis absorption, and fluorescence analyses.

## 2 Results and discussion

### 2.1 Synthesis

**Fig. 2:**
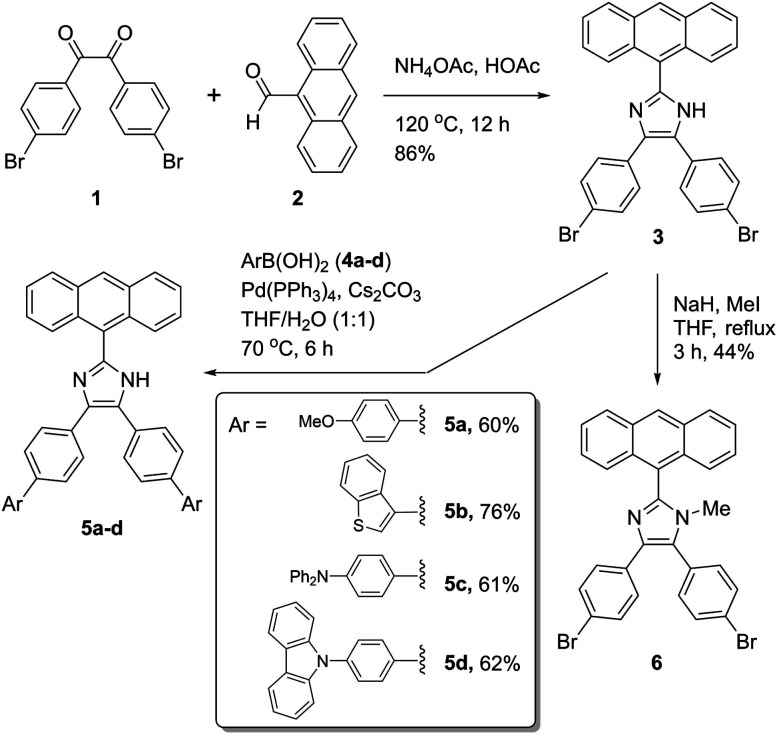
Synthesis of π-ADPI derivatives 5a–d and *N*-methylated ADPI 6.

2-(Anthracene-9-yl)-4,5-bis(4-bromophenyl)-1*H*-imidazole 3 was synthesized as a key intermediate for making diverse π-ADPIs (see Fig. 2). This imidazole intermediate was readily prepared through a one-pot condensation reaction between dione 1 and anthracene-9-carbaldehyde (2) in the presence of ammonium acetate and acetic acid, the reaction mechanism of which follows the multi-component Debus–Radziszewski imidazole synthesis.^28,29^ Upon refluxing at 120 °C for 12 hours, this condensation reaction afforded intermediate 3 in a high yield of 86%. The two bromo groups in 3 provide synthetic handles for transition metal-mediated cross-coupling reactions. In our work, Suzuki–Miyaura cross-coupling reactions were respectively conducted between 3 and four areneboronic acids (4a–d). The reaction conditions of the Suzuki reactions involved Pd(PPh_3_)_4_ as the catalyst, cesium carbonate as the base, and a mixture of THF and water as the solvent. All the coupling reactions went smoothly at 70 °C and accomplished within 6 hours. The resulting cross-coupled products 5a–d were then obtained after standard column chromatographic purification in satisfactory yields ranging from 60% to 76%. It is worth remarking that Suzuki–Miyaura cross-coupling reactions taking place on imidazole-containing substrates are often problematic (*e.g.*, low-yielding or lack of selectivity) owing to the presence of relatively acidic imidazolyl N–H group.^30–33^ In our cases here, the cross-coupling efficiency turned out to be really good, presumably due to steric effects delivered by the 9-anthryl group that is adjacent to the imidazolyl unit of 3. Imidazole is a versatile group that can act as both hydrogen bond donor and acceptor. For the purpose of comparative analysis in our structural and photophysical studies, we also prepared compound 6 which is an *N*-methylated derivative of ADPI 3. The synthesis of 6 was done through a substitution reaction by deprotonation of 3 with sodium hydride, followed by treatment with methyl iodide. The structures of all the prepared ADPI and π-ADPI compounds were elucidated by ^1^H NMR, ^13^C NMR, infrared (IR) spectroscopic, and high resolution mass spectrometric (HR-MS) analyses. Detailed characterization data are provided in the ESI.†

### 2.2 X-ray single crystallographic properties

**Fig. 3:**
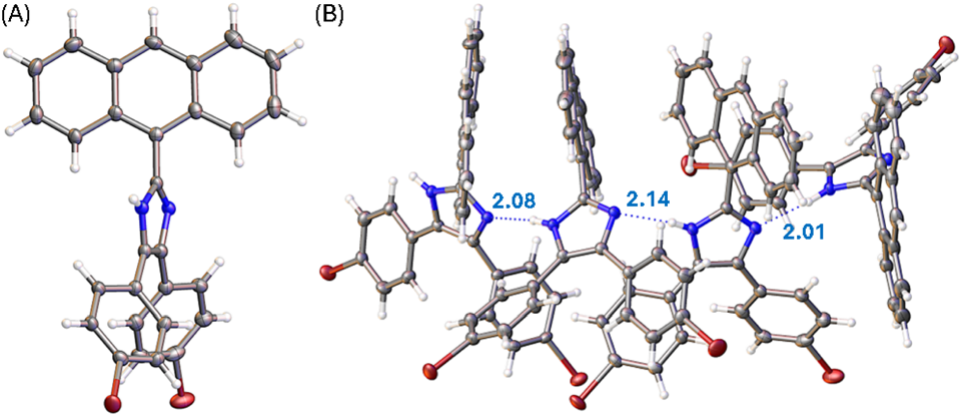
(A) ORTEP drawing (at 50% ellipsoid probability) of the molecular structure of 3 determined by single-crystal X-ray diffraction analysis. (B) Hydrogen bonding interactions in the crystal structure of 3 with hydrogen bond distances highlighted in Å. CCDC 2354158.

Single crystals of compounds 3, 5b, and 6 were successfully grown through slowly diffusing hexane into a CH_2_Cl_2_ solution of 3 or letting the solutions of 5b and 6 in CH_2_Cl_2_/THF (1 : 2, v/v) slowly evaporate at room temperature. The crystal structures of these compounds were then elucidated by XRD analysis to understand their molecular structural and solid-state packing properties. Fig. 3A illustrates one of the molecular structures in the unit cell of the single crystal of compound 3. The crystal structure of 3 presents a triclinic system with *P*-1 space group. As expected, the arene groups around the imidazole core in 3 all rotate significantly to avoid steric clashing. The imidazolyl group of 3 acts as both a hydrogen bond donor and a hydrogen bond acceptor, affording a network of hydrogen bonded assembly in the crystal structure as depicted by Fig. 3B. Along the hydrogen-bonded network, three similar hydrogen bonding interactions can be observed with N⋯H distances of 2.08, 2.14, and 2.01 Å, respectively. In the meantime, the intermolecular contact is further enhanced through π-stacking of anthryl rings.

**Fig. 4:**
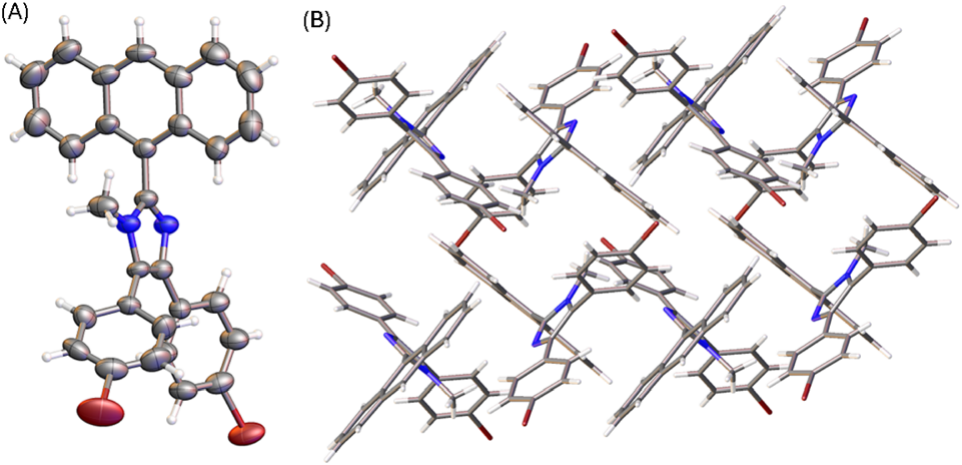
(A) ORTEP drawing (at 50% ellipsoid probability) of the molecular structure of 6 determined by single-crystal X-ray diffraction analysis. (B) Packing motif shown in the crystal structure of 6. CCDC 2354159.

The molecular structure of compound 6 is similar to that of 3 (see Fig. 4A). The presence of an *N*-methyl group makes the anthryl unit of 6 take a nearly perpendicular orientation with respect to the central imidazole ring at a torsion angle of 84°. The other two phenyl rings are somewhat less rotated, particularly the ring at the 4-position of imidazole shows a torsion angle of 20 to 23° with respect to the imidazole plane. The solid-state packing motif of 6, however, is very different from that of 5a. The crystal structure of 6 is in an orthorhombic system with *Pna*2_1_ space group. Because of *N*-methylation, the imidazole unit of 6 lacks the ability to form intermolecular hydrogen bonding interactions. The twisted conformation of 6 thus leads to relatively loose packing in the solid state, where the dominant intermolecular forces are C–H⋯π stacking as shown in Fig. 4B.

**Fig. 5:**
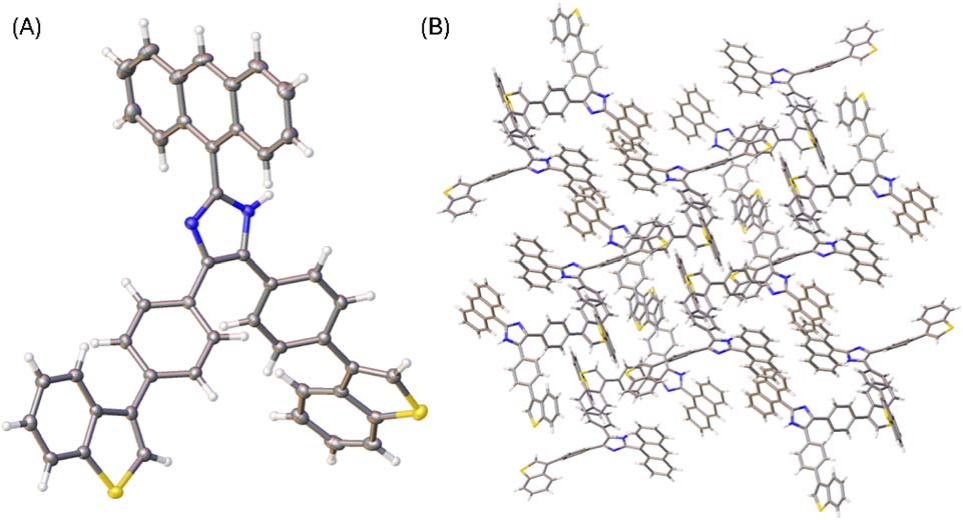
(A) ORTEP drawing (at 50% ellipsoid probability) of the molecular structure of 5b determined by single-crystal X-ray diffraction analysis. (B) Packing motif shown in the crystal structure of 5b. CCDC 2354160.

The X-ray determined molecular structure of 5b is shown in Fig. 5A. Like the other two compounds, the anthryl unit in 5b takes a perpendicular orientation relative to the central imidazole ring. The two phenyl rings show torsion angles of 35–39° with respective to the imidazole unit, and 36–40° to the two benzo[*b*]thiophene units, respectively. The steric bulkiness of the arene groups surrounding the imidazole ring of 5b hinders the formation of intermolecular hydrogen bonds among the molecules of 5b in the crystalline state. In the crystal structure, molecules of 5b are packed with a triclinic unit cell and *P*-1 space group. As shown in Fig. 5B, the packing motif of 5b has no significant π-stacking involved, but C–H⋯π interactions between benzo[*b*]thiophene units present notably.

**Fig. 6:**
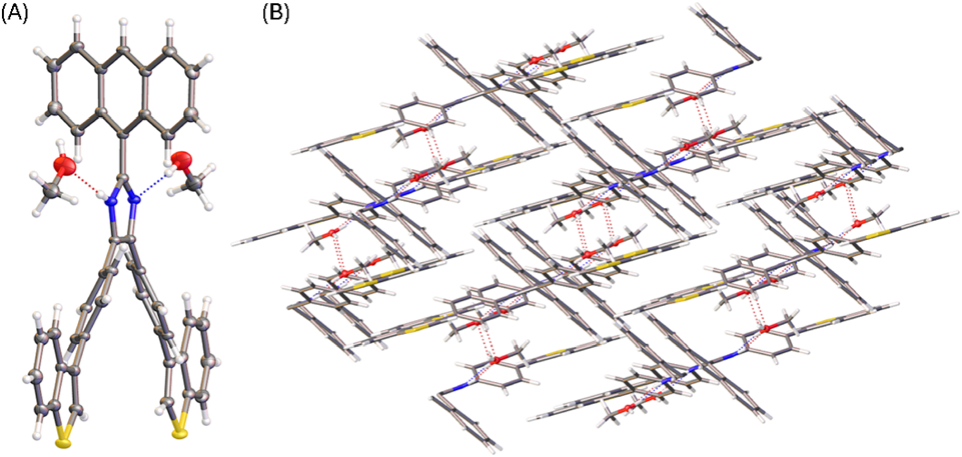
(A) ORTEP drawing (at 50% ellipsoid probability) of the molecular structure of 5b solvate determined by single-crystal X-ray diffraction analysis. (B) Packing motif shown in the crystal structure of 5b solvate. CCDC 2354150.

Diffusion of methanol into a THF solution of 5b produced solvates, the solid-state structure of which was determined by X-ray analysis. Fig. 6 shows the molecular structure of 5b where the imidazole unit interacts with two molecules of methanol through hydrogen bonds. Compared with the molecular conformation observed in the single crystal of 5b, the incorporation of methanol molecules in the crystal lattice causes the anthryl group to take a relatively small torsion angle of 57° with respect to the imidazole ring. It is also interesting to observe that the molecular conformation of 5b in the solvate structure possesses a *C*_2_ symmetry. As such, the solvate of 5b shows an intimate packing motif in the solid state, forming a monoclinic *I*2/*a* system. In the crystal packing, methanol molecules filled in the space between the molecules of 5b through hydrogen bonds (see Fig. 6B). The imidazole unit of 5b acts as both hydrogen donor and acceptor, showing intermolecular O⋯H (2.04 Å) and N⋯H (1.96 Å) interactions, respectively.

### 2.3 UV-Vis absorption and fluorescence spectroscopic properties

**Fig. 7:**
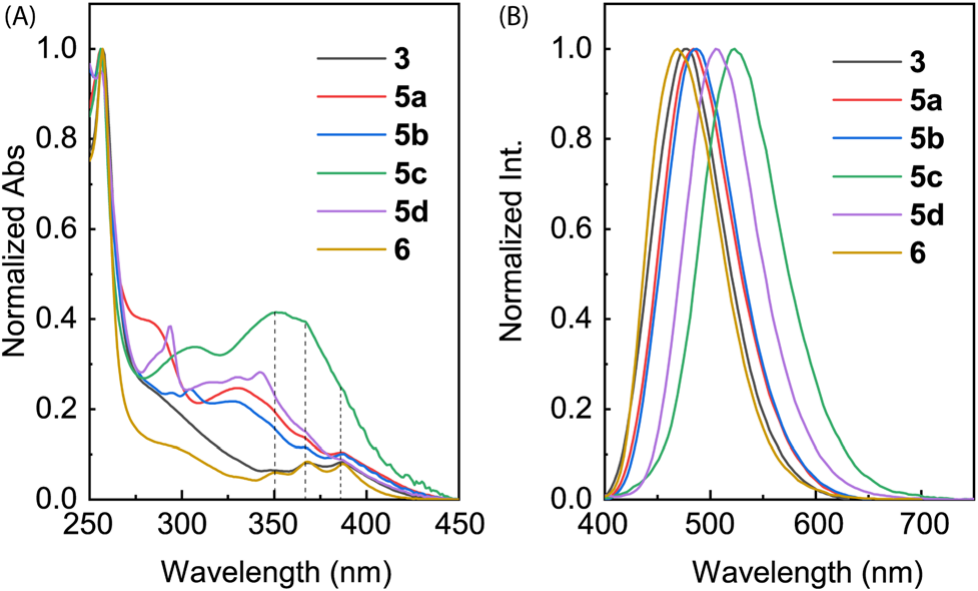
(A) Normalized UV-Vis absorption and (B) normalized fluorescence spectra (*λ*_ex_ = 360 nm) of compounds 3, 5a–d, and 6 measured in CH_2_Cl_2_ at room temperature.

The electronic absorption properties of compounds 3, 5a–d, and 6 were investigated by UV-Vis absorption spectroscopic analysis. As shown in Fig. 7A, the spectra of ADPIs 3 and 6 measured in CH_2_Cl_2_ show three vibronic bands at 388, 368, and 348 nm in the low-energy region, which are characteristic of the π → π* transitions at the anthryl unit. Similar absorption bands are discernible in the spectra of 5a–d in CH_2_Cl_2_ (highlighted by dashed lines in Fig. 7A), but they significantly overlap with the absorption bands of the arene groups appended to the ADPI scaffold. Detailed UV-Vis absorption data are summarized in Table 1.

**Table 1:** Summary of photophysical data of compounds 3, 5a–d, and 6. *λ*_abs_: wavelength of UV-Vis absorption peak; *ε*: extinction coefficient; *λ*_em_: wavelength of maximum emission; *Φ*: fluorescence quantum yield; *ν*: Stokes shift

Entry	*λ*_abs_/nm (*ε*/×10^3^ M^−1^ cm^−1^)	*λ*_em_/nm	*Φ*	*ν*/cm^−1^
3	387 (3.83), 368 (3.59), 348 (2.87), 304 (5.69)	477	0.095	4875
5a	387 (3.44), 366 (4.87), 350 (5.61), 333 (6.46), 280 (12.05)	484	0.088	5179
5b	386 (1.95), 368 (2.34), 329 (4.80), 305 (5.97), 293 (6.17)	486	0.166	5331
5c	351 (16.61), 307 (13.00)	522	0.289	6750
5d	386 (8.65), 342 (25.92), 332 (25.14), 293 (32.92)	506	0.132	6144
6	387 (2.76), 368 (2.80), 351 (2.12), 296 (3.76)	469	0.147	4518

Fig. 7B shows the normalized fluorescence spectra of 3, 5a–d, and 6 measured in CH_2_Cl_2_. As can be seen, all the emission spectra exhibit a smooth Gaussian-type profile with different maximum emission wavelengths (*λ*_em_) ranging from 469 to 522 nm (see Table 1 for details). These emission peaks are considerably redshifted than pristine anthracene, which typically shows a set of vibronic bands in the spectral region of 360–460 nm.^34^ According to the UV-Vis analysis, the excitation light should be dominantly absorbed by the anthracene unit, promoting it to the first excited state (S_1_) through vertical electronic transition.

It is interesting to note that the *λ*_em_ of 6 is blueshifted by 8 nm relative to 3, although they possess the same π-frameworks except that 6 is methylated at the imidazolyl unit and 3 has a free imidazole ring. Since both of them show nearly identical low-energy absorption bands, the difference in their maximum emission wavelengths can be explained by that the anthracene and imidazole rings of 6 in the first excited state are more twisted than that of 3 due to the *N*-methyl group of 6. For π-ADPIs 5a–d, the maximum emission wavelengths are further redshifted; in particular, the effects of strong electron-donating diphenylamine and carbazole groups in 5c and 5d are more significant than the others.

**Fig. 8:**
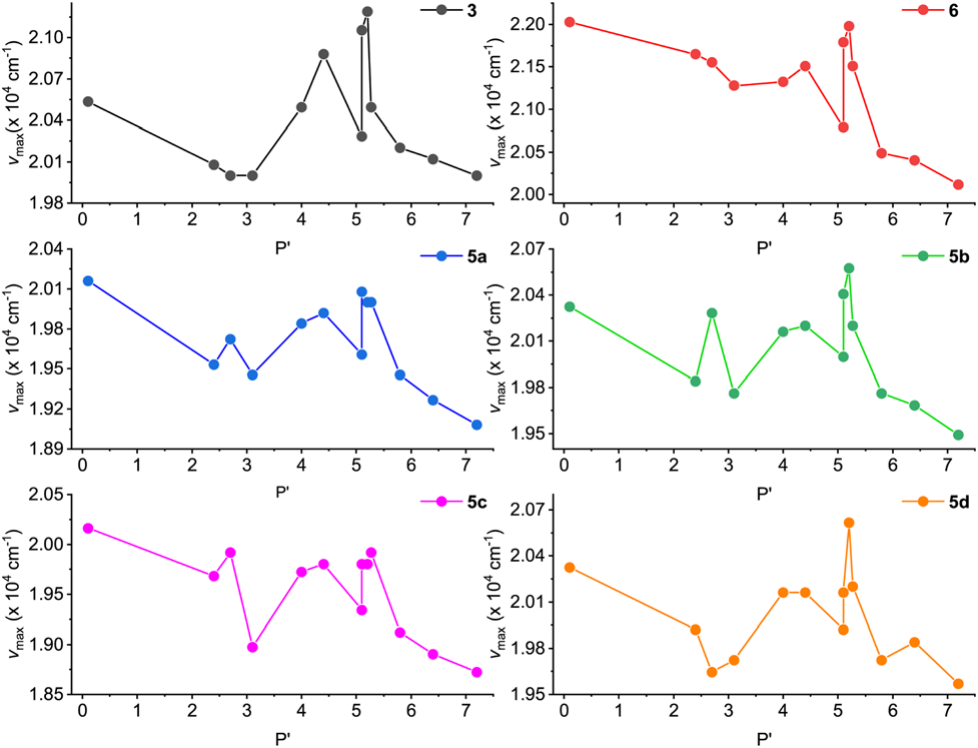
Plots of maximum emission energy (*ν*_max_) *versus* solvent polarity index (*P*′) for compounds 3, 5a–d, and 6.

In a previous study by Li and co-workers,^21^ the fluorescence of ADPIs was reported to exhibit solvatofluorochormic effects. In view of these properties, we also investigated the fluorescence behavior of our synthesized ADPIs and π-ADPIs in organic solvents with different degrees of polarity. Fig. 8 shows the correlations of the maximum emission energies of 3, 5a–d, and 6 with the polarity indexes (*P*′) of various solvents. For all of the six ADPI derivatives, the correlation plots exhibit a general trend of decreasing emission energy (*i.e.*, redshifted *λ*_em_) with increasing solvent polarity, but significant irregularities can be seen in the polarity range of 3–6; particularly, methanol and ethanol are the most notable outliers. The solvent-dependent fluorescence suggests that the emissive states of these compounds possesse intramolecular charge-transfer (ICT) character. As demonstrated in the X-ray analysis of 5b, protic solvents (methanol and ethanol) can form hydrogen bonding interactions with the imidazole C=N group and hence contribute more stabilization to the ground state. This effect in turn modifies the energy gap between the emissive state and the ground state to significantly affect the emission energy.

**Fig. 9:**
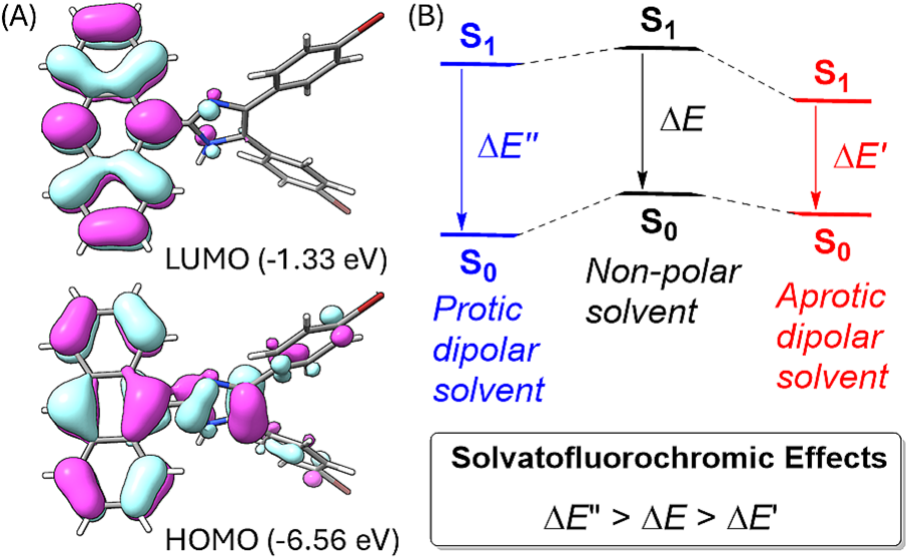
(A) Plots of frontier molecular orbitals (FMOs) of ADPI 3 calculated at the M06-2X/Def2-SVP level (isovalue = 0.03 a.u.). (B) Schematic illustration of the solvent effects on the energies of the ground (S_0_) state and the first excited state (S_1_) of an ADPI.

To better understand the solvatofluorochromic effects observed for the ADPIs and π-ADPIs, density functional theory (DFT) calculations were carried out on compound 3 and its frontier molecular orbital properties are illustrated in Fig. 9A. The highest occupied molecular orbital (HOMO) is distributed among the anthracene and imidazole units, while the lowest unoccupied molecular orbital (LUMO) is predominantly located in the anthracene unit. When an ADPI (*e.g.*, 3) is photoexcited, the vertical electronic transition contains a character of mainly HOMO → LUMO transition. From the FMO analysis, it is reasonable to say that the S_0_ → S_1_ transition should result in an ICT from imidazole to anthracene. Moreover, the S_1_ is more polar in nature than the S_0_ due to the occurrence of ICT. As such, an aprotic dipolar solvent should provide more stabilization on the S_1_ than the S_0_ state to reduce the energy gap and cause redshifted *λ*_em_. On the other hand, if the solvent is a hydrogen bond donor (*e.g.*, an alcohol), the ground-state ADPI can form significant hydrogen bonding interactions similar to the case observed in the methanol-solvated X-ray structure of 5b. These interactions, contrary to the polarity effect, stabilize the ground (S_0_) state more than the first excited (S_1_) state. In this case, the energy gap between S_0_ and S_1_ is widened and the *λ*_em_ is blueshifted. Overall, the observed solvatofluorochromic effects of 3, 5a–d, and 6 point toward a potential of utilizing these ADPI derivatives as a class of environment-sensitive fluorophores for probing different solvent properties.

### 2.4 Interactions of ADPI derivatives with trifluoroacetic acid

The imidazole group has an amphoteric character, allowing it to act as both an acid and a base.^8,35^ In view of this property, we subsequently investigated our synthesized ADPI derivatives in terms of their spectral responses to interactions with acids. In our experiments, a strong organic acid, trifluoroacetic acid (TFA), was used to protonate compounds 3, 5a–d, and 6, respectively, in organic solution, and the detailed processes were monitored by UV-Vis, fluorescence, and NMR analysis.

**Fig. 10:**
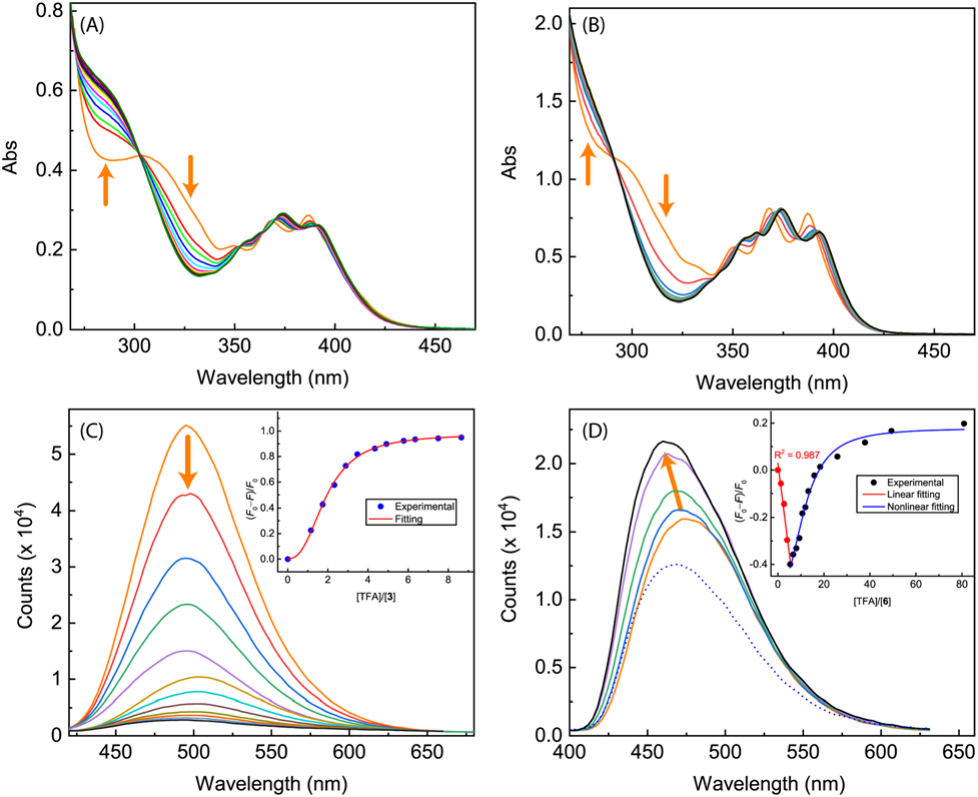
UV-Vis spectra monitoring the titrations of (A) compound 3 (1.40 × 10^−4^ M in CH_2_Cl_2_) with TFA (0 to 17.8 mole equiv.) and (B) compound 6 (7.90 × 10^−5^ M in CH_2_Cl_2_) with TFA (0 to 98.1 mole equiv.). (C) Fluorescence spectra monitoring the titration of compound 3 (1.40 × 10^−4^ M in CH_2_Cl_2_) with TFA (0 to 8.65 mole equiv.). (D) Fluorescence spectra monitoring the titration of compound 6 (7.90 × 10^−5^ M in CH_2_Cl_2_) with TFA (0 to 5.34 mole equiv., solid lines; at 80.9 mole equiv., dashed line). Arrows indicate the trends of spectral changes with increasing addition of TFA. Insets in (C) and (D) are plots of (*F*_0_ − *F*)/*F*_0_*vs.* the mole equiv. of TFA, where *F*_0_ is the fluorescence intensity at the maximum emission wavelength before titration and *F* is the fluorescence intensity at the same wavelength during titration.

Fig. 10 shows the results of titration of ADPI derivatives 3 and 6 with TFA. Interaction of compound 3 with TFA caused the three characteristic anthracene absorption bands in the low-energy region (350–390 nm) to be slightly redshifted by *ca.* 5 nm (see Fig. 10A). In the high-energy region of the absorption spectrum, two isosbestic points can be clearly seen at 303 and 270 nm, respectively. In contrast to the moderate changes observed in the UV-Vis titration, the fluorescence profile of 3 was found to be substantially quenched by TFA titration. As shown in Fig. 10C, the fluorescence intensity of 3 at the maximum emission wavelength is quenched by nearly 93% after interacting with 8.65 mole equiv. of TFA. The fluorescence quenching trend can be well described by a logistic nonlinear regression model (see the inset of Fig. 10C).

The UV-Vis absorption spectrum of *N*-methylated 6 varies in a similar way to that of 3 during the TFA titration (Fig. 10B), but the fluorescence of 6 responds to TFA titration quite differently. As shown in Fig. 10D, the fluorescence intensity of 6 increases linearly with the amount of added TFA from 0 to 5.34 mole equiv., along with a noticeable blueshift of the maximum emission wavelength. Further increase in TFA addition results in a trend of fluorescence quenching that follows a non-linear logistic model (see Fig. 10D). At the saturation point, the fluorescence of 6 is only quenched by *ca.* 20%, which is significantly different from the nearly quantitative quenching effect observed for ADPI 3.

**Fig. 11:**
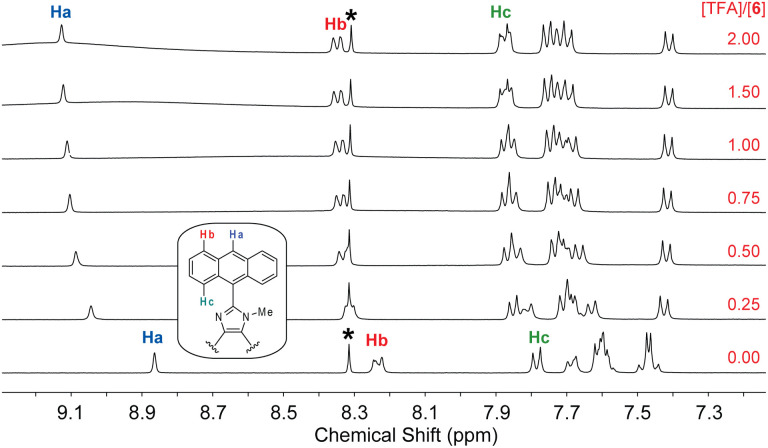
^1^H NMR (300 MHz, DMSO-*d*_6_) titration of 6 (5.57 mM) with TFA (0 to 2.0 mole equiv.) at room temperature. The singlet at 8.30 ppm (labelled by *) is due to residual DMF in the solution.

The fluorescence titration data indicates that the interactions of 6 with TFA undergo two separate stages. To better understand the protonation processes involved, ^1^H NMR titration of 6 with TFA was performed in deuterated DMSO. Fig. 11 shows the NMR titration results, where characteristic signals due to certain protons on the anthracene units (labelled as Ha–Hc) are highlighted to show the trend of spectral changes. When the amount of TFA added is 0.25 mole equiv., the aromatic proton signals are complex and broad, indicating a dynamic process of proton exchange on the imidazolyl unit. At this stage, it is possible that the presence of TFA induces hydrogen bonding interactions with 6, making the π-framework of 6 more twisted and less rotatable. The enhanced and blueshifted fluorescence observed in the early stage of fluorescence titration are consistent with this argument. When the amount of TFA is more than 0.50 mole equiv., the aromatic signals of 6 show better-resolved patterns in which the central proton (Ha) of the anthracene unit becomes significantly downfield-shifted to 9.11 ppm. The other anthryl protons are also downfield-shifted but in a less magnitude. This can be explained by that the imidazole is protonated on the C=N site, converting it into a more electron-withdrawing imidazolium ring. As a result of protonation, the excited state of 6 can be deactivated more efficiently through some non-radiative decay pathways to cause fluorescence quenching; for example, the photoinduced electron transfer from anthracene (donor) to imidazolium ion (acceptor).^36^

**Fig. 12:**
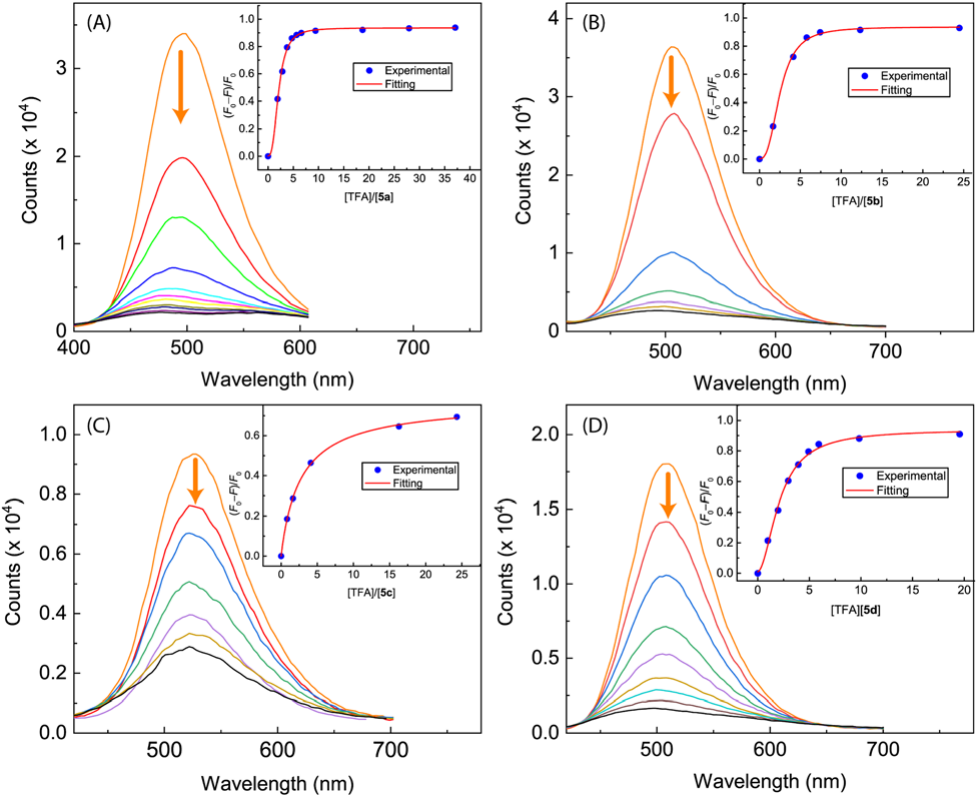
Fluorescence spectra monitoring the titrations of (A) compound 5a (8.62 × 10^−5^ M in CH_2_Cl_2_) with TFA (0 to 37.1 mole equiv.), (B) compound 5b (9.84 × 10^−5^ M in CH_2_Cl_2_) with TFA (0 to 24.5 mole equiv.), (C) compound 5c (9.91 × 10^−5^ M in CH_2_Cl_2_) with TFA (0 to 24.3 mole equiv.), and (D) compound 5d (8.25 × 10^−5^ M in CH_2_Cl_2_) with TFA (0 to 19.6 mole equiv.). Arrows indicate the trends of spectral changes with increasing addition of TFA. Insets are plots of (*F*_0_ − *F*)/*F*_0_*vs.* the mole equiv. of TFA, where *F*_0_ is the fluorescence intensity at the maximum emission wavelength before titration and *F* is the fluorescence intensity at the same wavelength during titration.

**Fig. 13:**
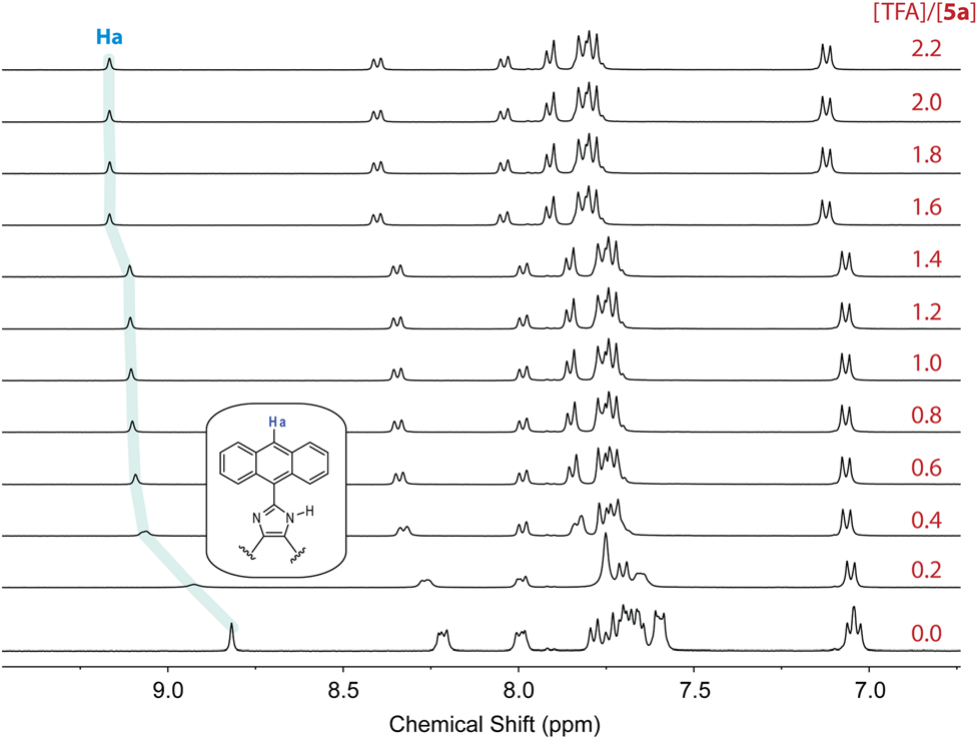
^1^H NMR (300 MHz, DMSO-*d*_6_) titration of 5a (5.57 mM) with TFA (0 to 2.2 mole equiv.) at room temperature.

In contrast to *N*-methylated ADPI 6, TFA titrations of ADPI 3 and its π-extended derivatives 5a–d all show similar fluorescence quenching behavior. As can be seen from Fig. 12, the fluorescence intensities of π-ADPIs 5a–b and 5d are nearly quantitatively quenched at the saturation point, which is similar to the results of ADPI 3. Obviously, the imidazolyl N–H group in these compounds plays a key role in the acid-induced quenching effect. It is interesting to note that ^1^H NMR monitoring the titration of 5a with TFA reveals three stages of change (see Fig. 13), which is different from the one-step protonation of ADPI 3 (see Fig. S-12 in the ESI†). It is likely that the anisolyl groups attached to compound 5a also participate in interactions with TFA and hence result in strong fluorescence quenching. In the case of 5c where diphenylamino groups are appendages, the fluorescence is quenched by *ca.* 70% at the saturation point. The exact reason for compound 5c to retain a relatively high level of fluorescence during TFA titration is not quite clear. However, it is tentatively proposed that protonation of the diphenylamino groups makes the structure of 5c more twisted and sterically hindered. This effect somewhat decelerates the non-radiative decays of the excited state of 5c.

### 2.5 Interactions of ADPI derivatives with fluoride anion

**Fig. 14:**
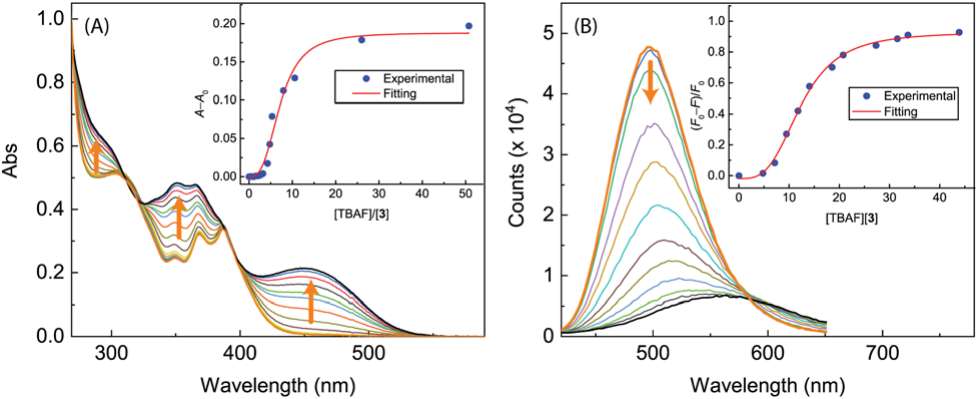
(A) UV-Vis spectra monitoring the titration of 3 (9.51 × 10^−5^ M in CH_2_Cl_2_) with TBAF (0.00 to 50.79 mole equiv.). Inset: plot of (*A* − *A*_0_) against the mole equiv. of TBAF, where *A*_0_ and *A* are the absorbance values measured at 450 nm before and during titration of TBAF. (B) Fluorescence spectra monitoring the titration of 3 (9.51 × 10^−5^ M in CH_2_Cl_2_) with TBAF (0.00 to 43.86 mole equiv.). Inset: plot of (*F*_0_ − *F*)/*F*_0_*vs.* the mole equiv. of TFA, where *F*_0_ is the fluorescence intensity at the maximum emission wavelength before titration and *F* is the fluorescence intensity at the same wavelength during titration. Arrows indicate the trends of spectral changes with increasing addition of TBAF.

The performance of free imidazole as a hydrogen bond donor^7,37^ inspired us to investigate the potential of ADPIs 3 and 5a–d as fluoride anion receptors and/or sensors, considering the strong hydrogen bond acceptor ability of fluoride anion.^38,39^Fig. 14 shows the UV-Vis and fluorescence titration results of ADPI 3 with tetrabutylammonium fluoride (TABF) in CH_2_Cl_2_. As can be seen from the UV-Vis data (Fig. 14A), a broad long-wavelength absorption band centering at *ca.* 450 nm emerges with increasing addition of TBAF to the solution of 3. This band can be attributed to the deprotonation of the imidazolyl N–H group by fluoride anion,^38^ which leads to enhanced π-electron delocalization and reduced HOMO–LUMO gap. In the meantime, the high-energy absorption bands due to anthracene in the range of 340–390 nm are also observed to grow in intensity with increasing titration of TBAF. The change in UV-Vis absorbance at 450 nm follows a nonlinear model using the Hill equation^40^ (see the plot in the inset of Fig. 14A).

The fluorescence titration results of 3 with TBAF are provided in Fig. 14B. During the titration, the emission intensity of 3 gradually deacreases with increasing amount of TBAF, showing a straightforward fluorescence quenching effect. The trend of fluorescence quenching at the maximum emission wavelength of 3 follows a nonlinear logistic model very well, and at the saturation point of titration the fluorescence is quenched by more than 80%. Moreover, the fluorescence spectral profiles show a significant redshift as the titration progresses. At the end of titration, the maximum emission wavelength is considerably shifted to 567 nm, indicating that the corresponding emissive state has a significant degree of ICT character.^41–43^

**Fig. 15:**
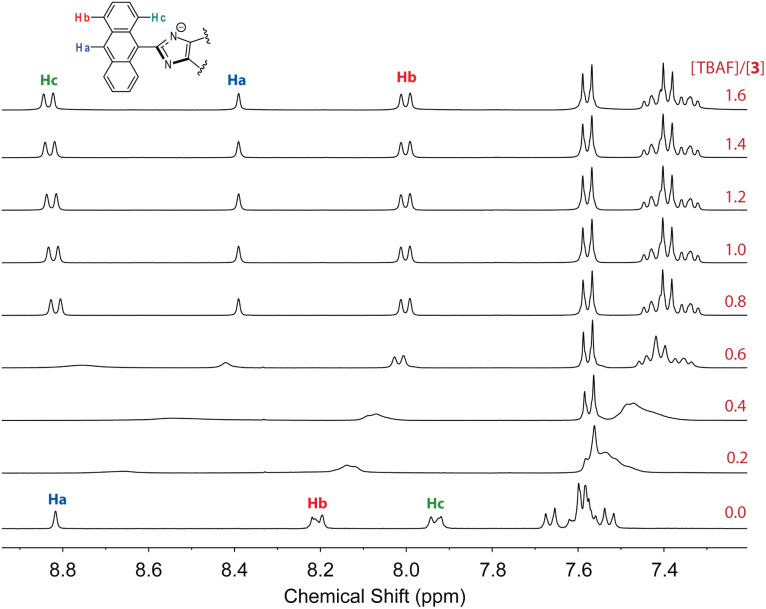
^1^H NMR (300 MHz, DMSO-*d*_6_) titration of 3 (3.61 mM) with TBAF (0 to 2.2 mole equiv.) at room temperature.

To further understand the interactions 3 with fluoride anion, ^1^H NMR titration experiments were carried out and the results are illustrated in Fig. 15. It is interesting to note that when only a small amount of TBAF (0.2–0.6 mole equiv.) was added to the solution of 3, the NMR signals appeared to be significantly broadened. This stage of NMR responses can be attributed to a rapid exchange between free ADPI 3 and its hydrogen bonded complex, [3⋯F^−^]. When the amount of TBAF was increased to more than 0.8 mole equiv., the NMR spetral profile showed well-resolved features, indicating another stage of interaction with fluoride anion. Given the basicity of fluoride anion, it is believed that the imidazolyl unit is deprotonated at the N–H site to yield an imidazole anion. It is worth noting that in the NMR spectrum, the central anthryl proton that is labelled as Ha gives a singlet at 8.82 ppm, while the other two anthryl protons labelled as Hb and Hc appear as two pseudo doublets at 8.21 and 7.93 ppm, respectively. During the titration, these peaks are considerably shifted. The singlet Ha and the proton adjacent to it, which is labelled as Hb, are both shifted to the upfield (8.39 and 8.00 ppm, respectively). The proton (labelled as Hc) that is close to the imidazole unit, however, is considerably downfield-shifted to 8.83 ppm. It has been reported that imidazole anion has a greater degree of aromaticity than neutral imidazole ring.^44,45^ Upon fluoride titration, the anthryl proton Hc of 3 is therefore subjected to increasing deshielding effect of imidazole anion ring current, which in turn causes its resonance frequency to be substantially downfield-shifted.

**Fig. 16:**
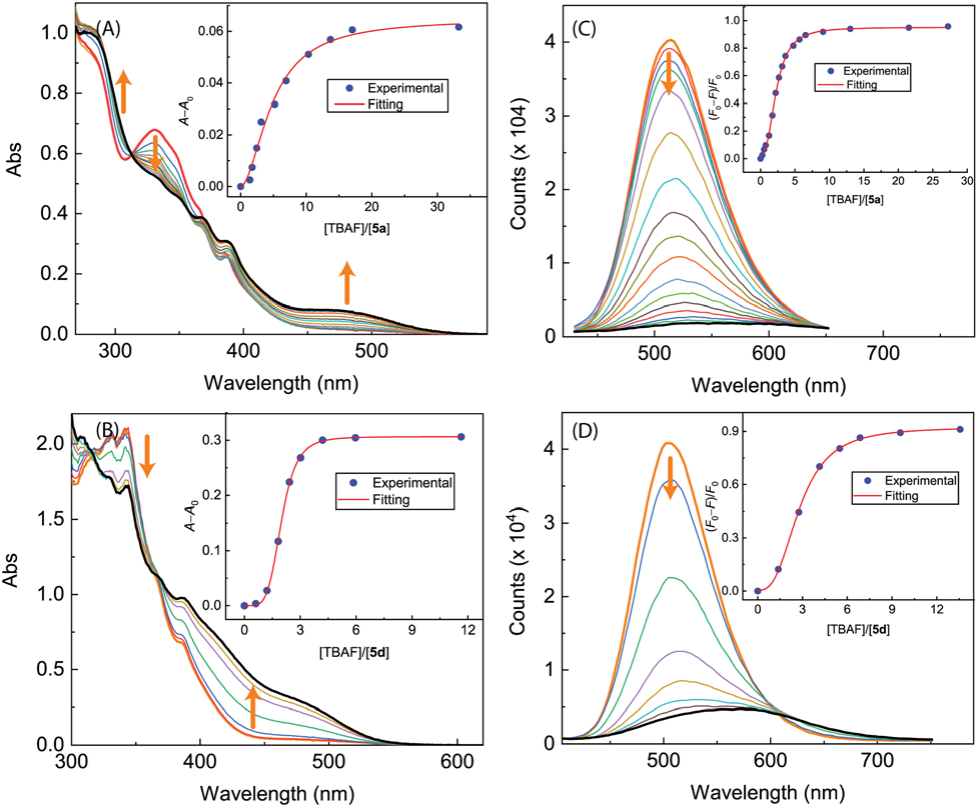
UV-Vis spectra monitoring the titrations of (A) 5a (8.62 × 10^−5^ M in CH_2_Cl_2_) with TBAF (0.00 to 33.26 mole equiv.) and (B) 5d (8.25 × 10^−5^ M in CH_2_Cl_2_) with TBAF (0.00 to 11.63 mole equiv.). Insets: plots of (*A* − *A*_0_) against the mole equiv. of TBAF, where *A*_0_ and *A* are the absorbance values measured at 470 nm and before and during titration of TBAF. Fluorescence spectra monitoring the titrations of (C) 5a (8.62 × 10^−5^ M in CH_2_Cl_2_) with TBAF (0.00 to 43.86 mole equiv.) and (D) 5d (8.25 × 10^−5^ M in CH_2_Cl_2_) with TBAF (0.00 to 11.56 mole equiv.). Insets: plot of (*F*_0_ − *F*)/*F*_0_*vs.* the mole equiv. of TFA, where *F*_0_ is the fluorescence intensity at the maximum emission wavelength before titration and *F* is the fluorescence intensity at the same wavelength during titration. Arrows indicate the trends of spectral changes with increasing addition of TBAF.

The results of UV-Vis and fluorescence titrations of π-ADPIs 5a and 5d with TBAF are shown in Fig. 16. Similar to compound 3, both 5a and 5d show a notable growth of a long-wavelength broad absorption band in their UV-Vis spectra, ranging from *ca.* 440 to 550 nm. The fluorescence spectra of 5a and 5d exhibit significant quenching in response to the titration of TBAF. For compound 5a, the fluorescence is almost quantitatively quenched at the saturation point of titration. Compound 5d shows fluorescence responses like those of 3; that is, the emission peak is redshifted significantly at the end of titration (see Fig. 16D).

**Fig. 17:**
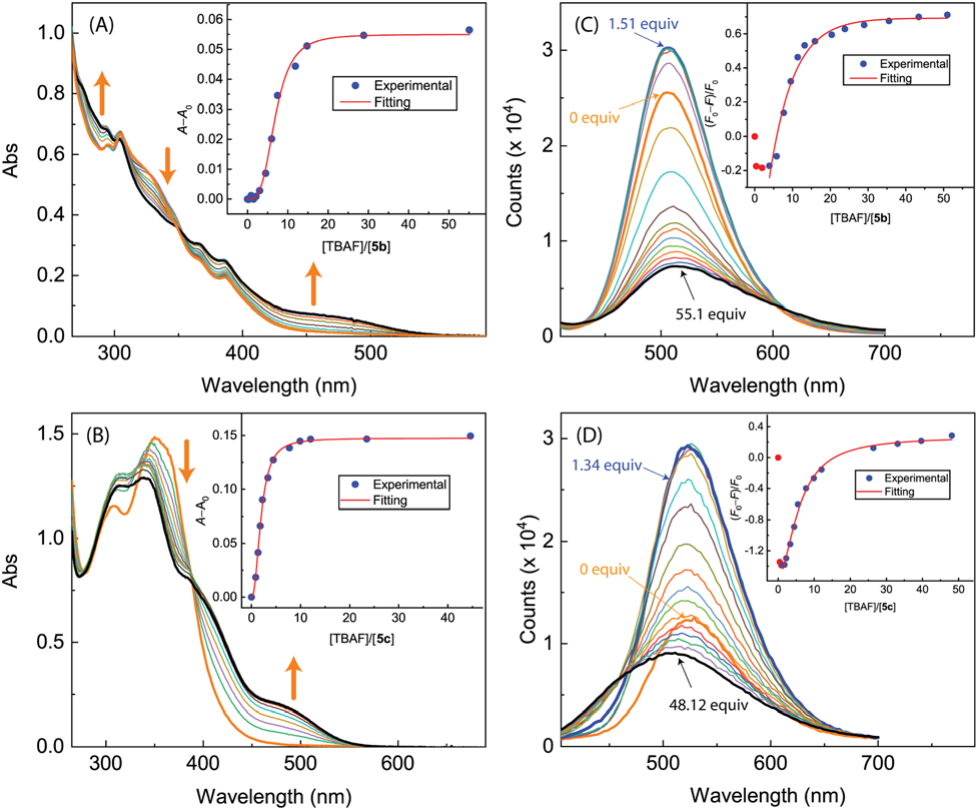
UV-Vis spectra monitoring the titrations of (A) 5b (1.17 × 10^−4^ M in CH_2_Cl_2_) with TBAF (0.00 to 50.99 mole equiv.) and (B) 5c (9.06 × 10^−5^ M in CH_2_Cl_2_) with TBAF (0.00 to 44.59 mole equiv.). Insets: plot of (*A* − *A*_0_) against the mole equiv. of TBAF, where *A*_0_ and *A* are the absorbance values measured at 500 nm before and during titration of TBAF. Fluorescence spectra monitoring the titrations of (C) 5b (1.17 × 10^−4^ M in CH_2_Cl_2_) with TBAF (0.00 to 50.99 mole equiv.) and (D) 5c (9.06 × 10^−4^ M in CH_2_Cl_2_) with TBAF (0.00 to 62.17 mole equiv.). Insets: plot of (*F*_0_ − *F*)/*F*_0_*vs.* the mole equiv. of TFA, where *F*_0_ is the fluorescence intensity at the maximum emission wavelength before titration and *F* is the fluorescence intensity at the same wavelength during titration. Arrows indicate the trends of spectral changes with increasing addition of TBAF.

As can be seen from Fig. 17A and B, the UV-Vis spectra of 5b and 5c exhibit similar behaviors to the other ADPI derivatives during TBAF titrations. The fluorescence spectra of 5b and 5c, however, show very different trends at the early stage of titration. Interestingly, both compounds exhibit fluorescence enhancement when they interact with a relatively small amount of TBAF. Especially, the fluorescence of compound 5c is increased by more than one fold when interacting with *ca.* 1.5 mole equiv. of TBAF. As the titration continues, the fluorescence behavior changes to a trend of quenching that is similar to the other ADPI derivatives. It is also interesting to comment that the maximum emission band of 5c at the end of titration is notably blueshifted relative to that of pristine 5c. Clearly, the electron-donating diphenylamino groups in 5c are responsible for this unusual spectral behavior. It is possible that the electron pushing between the diphenylamino and anionic imidazole ring causes the molecular framework to be more twisted and less π-conjugated.

## 3 Conclusions

We herein report a systematic study of a series of ADPI derivatives that contain π-extended units and appendage groups with different electronic effects. These compounds can be readily accessed through concise synthetic routes with satisfactory to good yields. X-ray single crystallographic analysis shows that the ADPI compounds take twisted molecular conformations and are packed in very different motifs depending on the appendage groups attached. All of these compounds show significant solvatofluorochromic effects that can be potentially useful as molecular probes for solvent polarity. The amphoteric properties of the imidazole unit in these compounds allow them to interact with strong protic acids (*e.g.*, TFA) and hard anionic species (*e.g.*, fluoride anion). Significant UV-Vis and fluorescence responses to TFA and fluoride anion have been thoroughly examined and analyzed. Our results point to the applicability of π-ADPIs in achieving colorimetric and fluorescence sensing of various acidic and/or anionic species. Moreover, the observations of some ADPI derivatives showing unexpected fluorescence enhancement in response to TFA or TBAF titrations indicate that the appendage groups in these compounds play an important role in dictating their photophysical properties. It is therefore anticipated that preparation of more arene-appended π-ADPIs would allow diversely behaving fluorophores to be attained, which may be further applied to form sensor arrays for rapid and accurate detection of chemical species of interest. Studies along this direction are underway.

## Data availability

Relevant data are within the paper and its ESI† files. Other data that supports the findings of this study are available from the corresponding author upon request.

## Conflicts of interest

There are no conflicts to declare.

## Supplementary Material

RA-014-D4RA03735A-s001

RA-014-D4RA03735A-s002
