# Supplementary material for: Synthesis and characterization of 2-(anthracene-9-yl)-4,5-diphenyl-1H-imidazole derivatives as environmentally sensitive fluorophores
Source: RSC Adv. 2024 Jul 26;14(32):23511–9. doi: 10.1039/d4ra03735a (PMC11275565; doi:10.1039/d4ra03735a)
Supplement: RA-014-D4RA03735A-s001 [file RA-014-D4RA03735A-s001.pdf]

Electronic Supplementary Material (ESI) for RSC Advances.
This journal is © The Royal Society of Chemistry 2024

Supporting Information for

Synthesis and characterization of 2-(anthracene-9-yl)-4,5-diphenyl-1*H*-imidazole derivatives as environmentally sensitive fluorophores

Eyad A. Younes,[*a] Amneh M. AL-Snaid,[a] Kayed A. Abu-Safieh,[a] Fatemeh Salami,[b] Nayyef Aljaar,[a] and Yuming Zhao[b]

[a]Department of Chemistry, Faculty of Science, The Hashemite University, P.O. Box 330127, Zarqa 13133, Jordan. E-mail: e.younes@hu.edu.jo; Tel: +962 (5) 3903333 ext. 4572

[b]Department of Chemistry, Memorial University of Newfoundland, St. John's, NL, Canada A1C 5S7. E-mail: yuming@mun.ca; Tel: +1 709 864 8747

Table of Content

1. Experimental Details S-2
2. NMR Spectra of Compounds **3, 5a-d,** and **6** S-7
3. ^{1}H NMR Titration Result of **3** with TBAF S-15
4. High-Resolution Mass Spectra for Compounds **3, 5a-d,** and **6** S-16
5. DFT Calculations of Compound **3** S-21
6. Crystallographic Data and Detailed Refinements S-23

1. Experimental Details

1.1 Materials and instrumentation

All chemicals, solvents and reagents were obtained of standard quality from commercial suppliers (Sigma Aldrich, Fluka, Acros, and Tedia) and were used as received without any further purification. All reactions were conducted in standard, dry glassware and under an inert atmosphere of nitrogen unless otherwise noted. Melting points (m.p.) were determined using a Stuart Scientific (Britain) melting point apparatus with samples placed in one-end open glass capillaries. Thin-layer chromatography (TLC) was performed with silica gel F254 covered on aluminum sheets and visualized by UV light. ^{1}H and ^{13}C NMR spectra were recorded at room temperature on a Bruker III 400 MHz ADVANCE spectrometer. Chemical shifts (δ) are reported in ppm downfield relative to the signal of an internal reference ($SiMe_4$). Coupling constants (*J*) are given in Hz. Fourier transform infrared (FTIR) spectra were recorded on a Bruker vertex 70 instrument. High-resolution APPI-TOF MS analysis was conducted on a Micromass GCT Premier instrument.

UV-Vis absorption spectra were recorded on a Cary 100 bio spectrophotometer. Solution-phase fluorescence spectra were measured using a Photon Technology International (PTI) QuantaMaster spectrofluorometer. Relative fluorescence quantum yields (Φ_f) were determined by the following equation:

$$\Phi_f = \Phi_s \times \frac{I_s}{I_r} \times \frac{A_r}{A_s} \times \frac{n_s^2}{n_r^2}$$

where Φ_f is the fluorescence quantum yield, *I* is the integrated area under the emission profile, *A* is the absorbance at a particular excitation wavelength, *n* is the refractive index of the medium. The subscripts s and r denote sample and reference, respectively. Quinine sulfate (Φ_f = 0.546) was used as the reference.

Single-crystal XRD data was collected at 100 (2) K on a XtaLAB Synergy-S, Dualflex, HyPix-6000HE diffractometer using Cu Kα radiation (λ = 1.5406 Å). The crystal was mounted on nylon CryoLoops with Paraton-N. Data collection and reduction were processed within CrysAlisPro (Rigaku OD, 2020). A multi-scan absorption correction was applied to the collected

reflections. Using the Olex2[1] software package, structures were solved with the ShelXT[2] and ShelXL[3] programs.

1.2 Synthesis

2-(Anthracene-9-yl)-4,5-bis(4- bromophenyl)-1H-imidazole (3)

In sealed tube a mixture of 4,4-dibromobenzil (0.15 g, 0.40 mmol), 9-anthracene carboxaldehyde (0.11 g, 0.52 mmol), ammonium acetate (0.50 g, 6.0 mmol), and acetic acid (4.0 mL) was heated at 120 °C. The mixture was stirred for 12 h. The reaction mixture was then poured into 100 mL of water. The resulting yellow precipitate was filtered and recrystallized from hot chloroform to offer compound **3** as a yellow crystal (0.19 g, 0.34 mmol, 86% yield). m. p = 164-166 °C. ^{1}H NMR (400 MHz, DMSO-d_6): δ 13.11 (s, 1H), 8.91 – 8.68 (m, 1H), 8.21 (d, *J* = 5.8 Hz, 2H), 7.94 (d, *J* = 5.9 Hz, 2H), 7.76–7.45 (m, 12H). ^{13}C NMR (100 MHz, DMSO-d_6): δ 144.34, 136.72, 134.85, 132.31, 131.86, 131.31, 131.25, 130.46, 129.80, 128.93, 127.51, 127.21, 126.38, 126.09, 121.37, 120.26. FTIR (KBr): 3387, 3048, 1493 cm^{-1}. HRMS (LC-TOF, positive mode) *m/z* calcd for $C_{29}H_{19}Br_2N_2$ 554.9895; found $[M + H]^{+\cdot}$ 554.9893.

General synthetic procedure for π-ADPIs 5a–d

A mixture of **3** (0.20 g, 0.30 mmol), areneboronic acid (**4a**–**d**), $Pd(PPh_3)_4$ (0.015 g, 0.013 mmol) and cesium carbonate (0.35 g, 1.1 mmol) in THF/H_2O (1:1, 30 mL) were bubbled with nitrogen gas for 10 min. The reaction mixture was heated at 70 °C under nitrogen protection and stirring for 6 h. The reaction was cooled down to room temperature. After evaporation of the THF, the product was extracted with chloroform (2 ×10 mL). The organic layer was removed under vacuum and the crude product was purified with silica gel chromatography using ethyl acetate and *n*-hexane (1:3, v/v) as eluent.[4]

2-(Anthracen-9-yl)-4,5-bis(4’-methoxy-[1,1’- biphenyl]-4-yl)-1*H*-imidazole (5a)

Compound **5a** was synthesized reacting **3** (0.20 g, 0.30 mmol) with 4-methoxyphenylboronic acid (**4a**, 0.164 g, 1.08 mmol), following the general procedure described above. Compound **5a** was obtained as a yellow powder (0.11 g, 0.18 mmol, 60% yield). m.p. = 170-172 °C. ^{1}H NMR (400 MHz, DMSO-d_6): δ 12.98 (s, 1H), 8.81 (s, 1H), 8.21 (dd, *J* = 7.3, 2.4 Hz, 2H), 8.06–7.92 (m, 2H), 7.79 (d, *J* = 8.4 Hz, 2H), 7.76–7.63 (m, 10H), 7.63–7.55 (m, 4H), 7.04 (dd, *J* = 8.8, 7.0 Hz, 4H), 3.81 (d, *J* = 3.5 Hz, 6H). ^{13}C NMR (100 MHz, DMSO-d_6): δ 159.54,

159.32, 143.89, 139.19, 138.28, 137.30, 134.44, 132.66, 132.21, 131.40, 131.33, 129.96, 128.89, 128.74, 128.30, 128.07, 127.93, 127.07, 126.75, 126.54, 126.41, 126.05, 114.92, 114.86, 55.67, 55.65. FTIR (KBr): 3375, 3032, 1501, 1246 cm^{-1}. HRMS (LC-TOF, positive mode) *m/z* calcd for $C_{43}H_{33}N_2O_2$ 609.2542; found $[M + H]^{+\cdot}$ 609.2561.

2-(Anthracen-9-yl)-4,5-bis(4- (benzo[*b*]thiophen-3-yl) phenyl)-1*H*-imidazole (5b)

Compound **5b** was synthesized by reacting **3** (0.20 g, 0.30 mmol) with benzo[*b*]thiene-3-ylboronic acid (**4b**, 0.196 g, 1.10 mmol), following the general procedure described above. Compound **5b** was obtained as a yellow-green powder (0.15 g, 0.23 mmol, 76% yield). m.p. = 163-165 °C. ^{1}H NMR (400 MHz, DMSO-d_6): δ 13.13 (s, 1H), 8.83 (s, 1H), 8.23 (dd, *J* = 6.6, 3.0 Hz, 2H), 8.10 (t, *J* = 6.3 Hz, 2H), 8.06 – 7.96 (m, 4H), 7.93 (d, *J* = 7.4 Hz, 3H), 7.89 – 7.81 (m, 3H), 7.75 (d, *J* = 8.1 Hz, 2H), 7.67 (d, *J* = 8.1 Hz, 2H), 7.61 (dd, *J* = 6.7, 3.1 Hz, 4H), 7.46 (h, *J* = 7.1, 6.6 Hz, 4H). ^{13}C NMR (101 MHz, DMSO) δ 144.14, 140.68, 137.65, 137.52, 137.38, 137.34, 136.93, 135.25, 134.95, 133.99, 131.39, 131.33, 130.80, 129.17, 128.93, 128.86, 128.82, 128.27, 128.17, 127.17, 126.53, 126.38, 126.11, 125.48, 125.25, 125.17, 125.12, 125.05, 124.93, 123.82, 123.77, 123.03, 122.93. FTIR (KBr): 3368, 3032, 1534 cm^{-1}. HRMS (LC-TOF, positive mode) *m/z* calcd for $C_{45}H_{29}N_2S_2$ 661.1772; found $[M + H]^{+\cdot}$ 661.1768.

4’,4”’-(2-(Anthracen-9-yl)-1*H*-imidazole-4,5-diyl)bis(*N*,*N*-diphenyl-[1,1’-biphenyl]-4-amine) (5c)

Compound **5c** was synthesized by reacting **3** (0.20 g, 0.30 mmol) with 4-(diphenylamino)phenylboronic acid (**4c**, 0.313 g, 1.08 mmol), following the general procedure described above. Compound **5c** was obtained as a yellow-green powder (0.16 g, 0.18 mmol, 61% yield). m.p. = 157-159 °C. ^{1}H NMR (400 MHz, DMSO-d_6): δ 13.03 (s, 1H), 8.81 (s, 1H), 8.26 – 8.15 (m, 2H), 8.05 – 7.90 (m, 2H), 7.84 – 7.61 (m, 12H), 7.62 – 7.53 (m, 4H), 7.33 (t, *J* = 7.5 Hz, 8H), 7.16 – 6.89 (m, 16H).^{13}C NMR (101 MHz, DMSO) δ 147.53, 147.48, 147.34, 147.07, 143.96, 138.95, 138.02, 137.29, 134.61, 134.23, 133.65, 131.37, 131.30, 130.08, 128.90, 128.30, 128.08, 127.93, 127.83, 127.12, 126.79, 126.53, 126.45, 126.08, 124.67, 124.57, 123.91, 123.78, 123.70, 123.67. FTIR (KBr): 3368, 3053, 1589 cm^{-1}. HRMS (LCTOF, positive mode) *m/z* calcd for $C_{65}H_{46}N_4$ 882.3722; found $[M]^{+\cdot}$ 882.3723.

9,9'-((2-(anthracen-9-yl)-1*H*-imidazole-4,5-diyl)bis([1,1'-biphenyl]-4',4-diyl))bis(9*H*-carbazole) (5d)

Compound **5d** was synthesized by reacting **3** (0.20 g, 0.30 mmol) with 4-(9-carbazolyl)benzene boronic acid (0.31 g, 1.079 mmol), following the general procedure described above. Compound **5d** was obtained as a yellow powder (0.16 g, 0.18 mmol, 62% yield). m.p. = 240-242 °C. ^{1}H NMR (400 MHz, DMSO-d_6): δ 13.12 (s, 1H), 8.84 (s, 1H), 8.26 (dd, *J* = 13.8, 7.2 Hz, 6H), 8.06 (m, 6H), 8.01–7.82 (m, 8H), 7.75 (t, *J* = 8.8 Hz, 4H), 7.67–7.58 (m, 4H), 7.48 (d, *J* = 3.3 Hz, 8H), 7.36–7.26 (m, 4H). ^{13}C NMR (100 MHz, DMSO-d_6): δ 144.22, 140.62, 139.42, 138.98, 138.68, 137.75, 137.41, 136.82, 136.54, 135.48, 131.42, 131.34, 131.00, 129.12, 128.93, 128.68, 128.53, 127.61, 127.53, 127.16, 126.78, 126.41, 126.09, 123.30, 121.04, 120.63, 120.59, 110.18. FTIR (KBr): 3042, 2956, 1601, 1504 cm^{-1}. HRMS (LC-TOF, positive mode) *m/z* calcd for $C_{65}H_{43}N_4$ 879.3488; found $[M + H]^{+\cdot}$ 879.3479.

2-(Anthracen-9-yl)-4,5-bis(4-bromophenyl)-1-methyl-1*H*-imidazole (6)

Compound **6** was prepared according to the literature procedure[5] with modifications. To a dried two-neck round-bottomed flask were added **3** (0.15 g, 0.27 mmol), anhydrous THF (15 mL), and NaH (0.01 g, 0.4 mmol). The resulting mixture was refluxed under a nitrogen for 1 h. To this mixture, iodomethane (0.044 g, 0.30 mmol) was added and refluxed for another 3 h. The reaction was then quenched by adding water (15 mL) and extracted with dichloromethane (30 mL). The organic layer was washed with a saturated Na_2SO_3 solution (180 mL). The separated organic layer was dried using magnesium sulfate. After removing the solvent, the crude product was purified using silica gel chromatography column to yielded **6** (0.067 g, 0.12 mmol, 44% yield) as a yellow solid. ^{1}H NMR (400 MHz, DMSO-d_6) δ: 8.87 (s, 1H), 8.23 (d, *J* = 7.1 Hz, 2H), 7.79 (d, *J* = 8.3 Hz, 2H), 7.69 (d, *J* = 9.1 Hz, 2H), 7.64–7.55 (m, 6H), 7.54 – 7.41 (m, 4H), 3.02 (s, 3H). ^{13}C NMR (100 MHz, DMSO-d_6): δ 145.06, 136.35, 134.37, 133.43, 132.73, 131.73, 131.62, 131.26, 130.21, 129.53, 129.27, 129.17, 128.63, 127.69, 126.19, 125.91, 124.73, 122.98, 119.84, 32.21. FTIR (KBr): 3050, 3002, 2947, 1590, 1544, 1420 cm^{-1}. HRMS (LC-TOF, positive mode) *m/z* calcd for $C_{30}H_{21}Br_2N_2$ 569.0051; found $[M + H]^{+\cdot}$ 569.0034.

References:

1. O. Dolomanov, L. Bourhis, R. Gildea and J. Howard, *J. Appl. Crystallogr.*, 2009, **42**, 339–341.

2. G. M. Sheldrick, *Acta Crystallogr. A*, 2015, **71**, 3–8.
3. G. M. Sheldrick, *Acta Crystallogr. C*, 2015, **71**, 3–8.
4. E. A. Younes, Y. J. Hammad, F. Salami, A. J. Rasras, R. A. Al-Qawasmeh and Y. Zhao, *RSC Adv.*, 2024, **14**, 5331–5339.
5. M. Toba, T. Nakashima and T. Kawai, *J. Polym. Sci. A: Polym. Chem.*, 2011, **49**, 1895–1906.

2. NMR Spectra of Compounds **3**, **5a-d**, and **6**

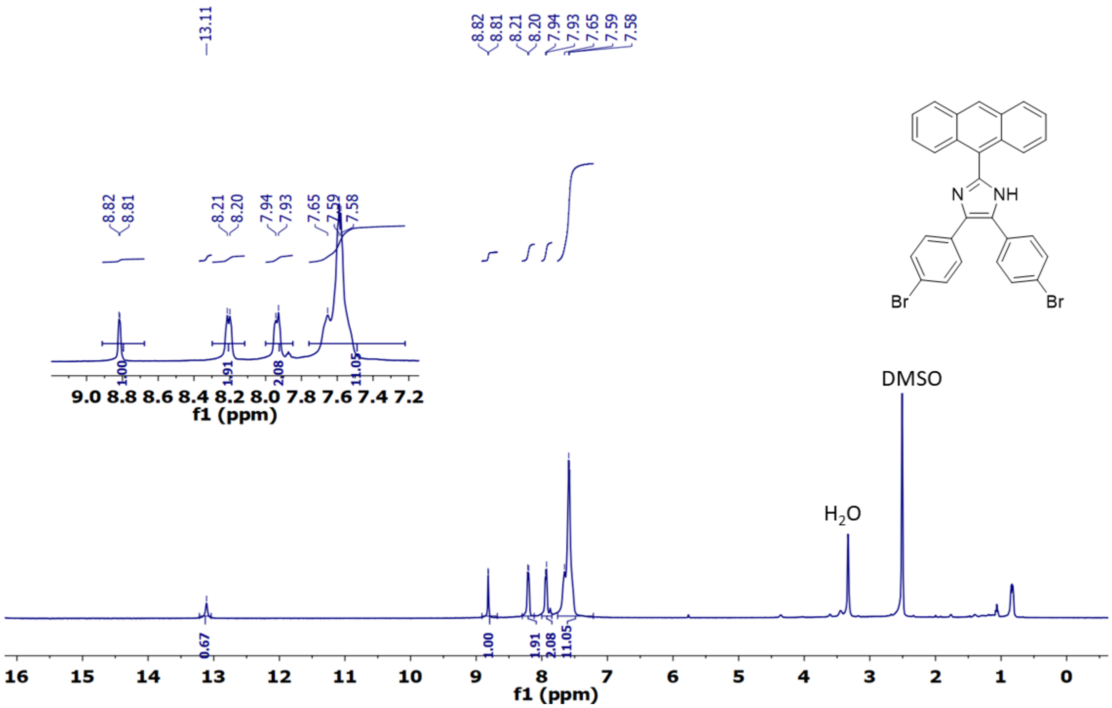

Fig. S-1 ^{1}H NMR (400 MHz, DMSO-d_6) spectrum of compound **3**.

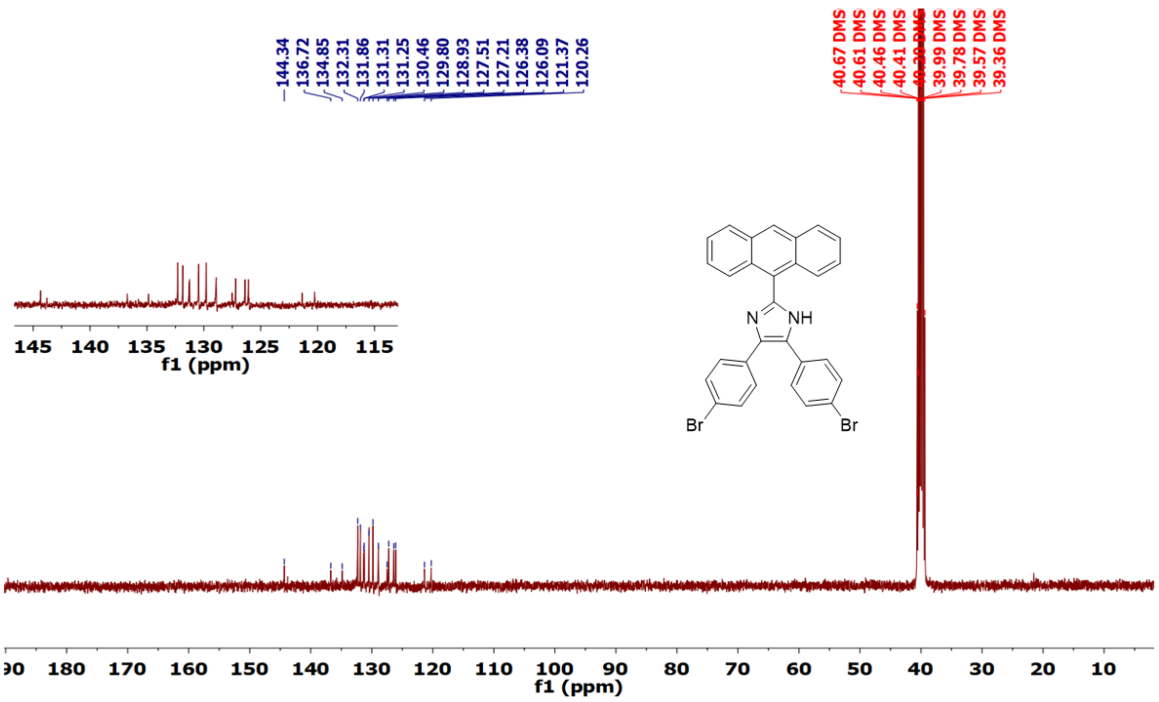

Fig. S-2 ^{13}C NMR (100 MHz, DMSO-d_6) spectrum of compound **3**.

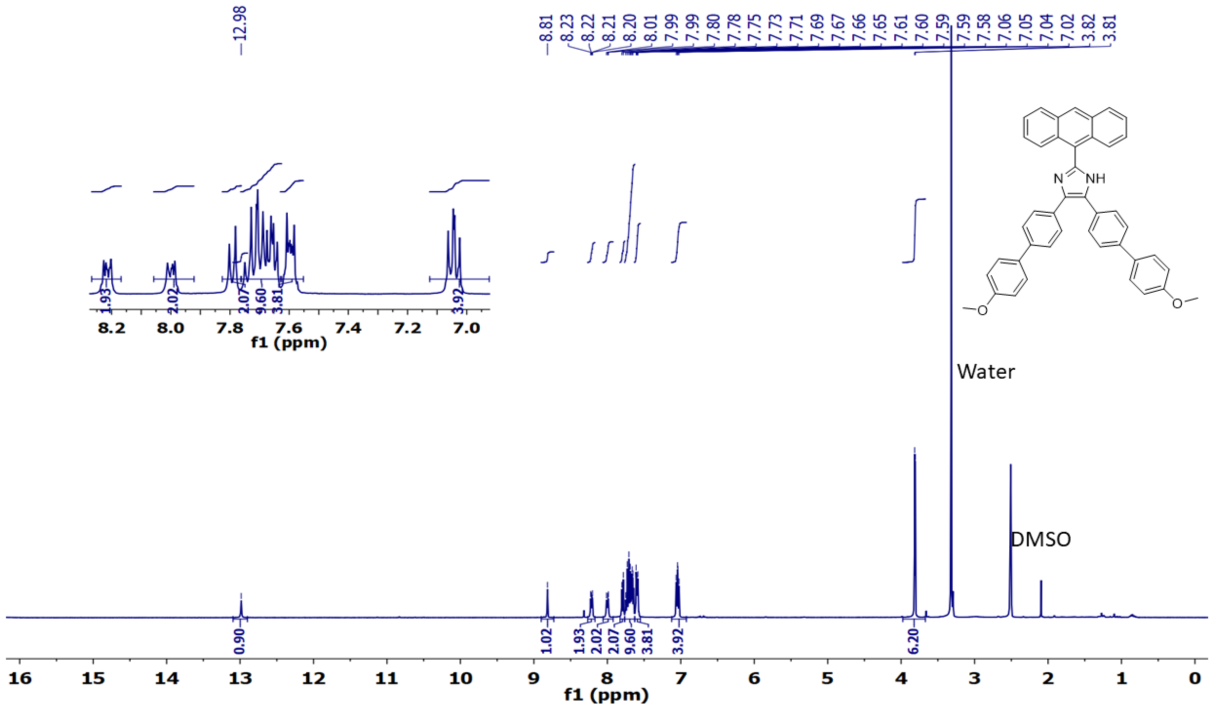

Fig. S-3 ^{1}H NMR (400 MHz, DMSO-d_6) spectrum of compound **5a**.

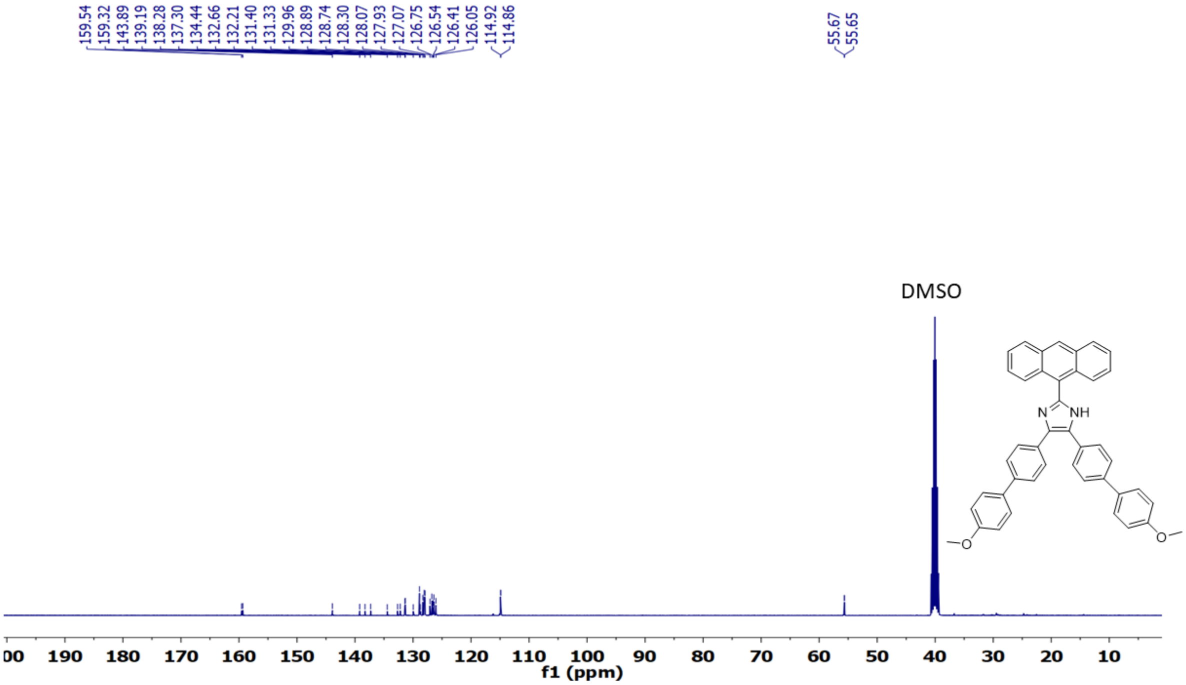

Fig. S-4 ^{13}C NMR (100 MHz, DMSO-d_6) spectrum of compound **5a**.

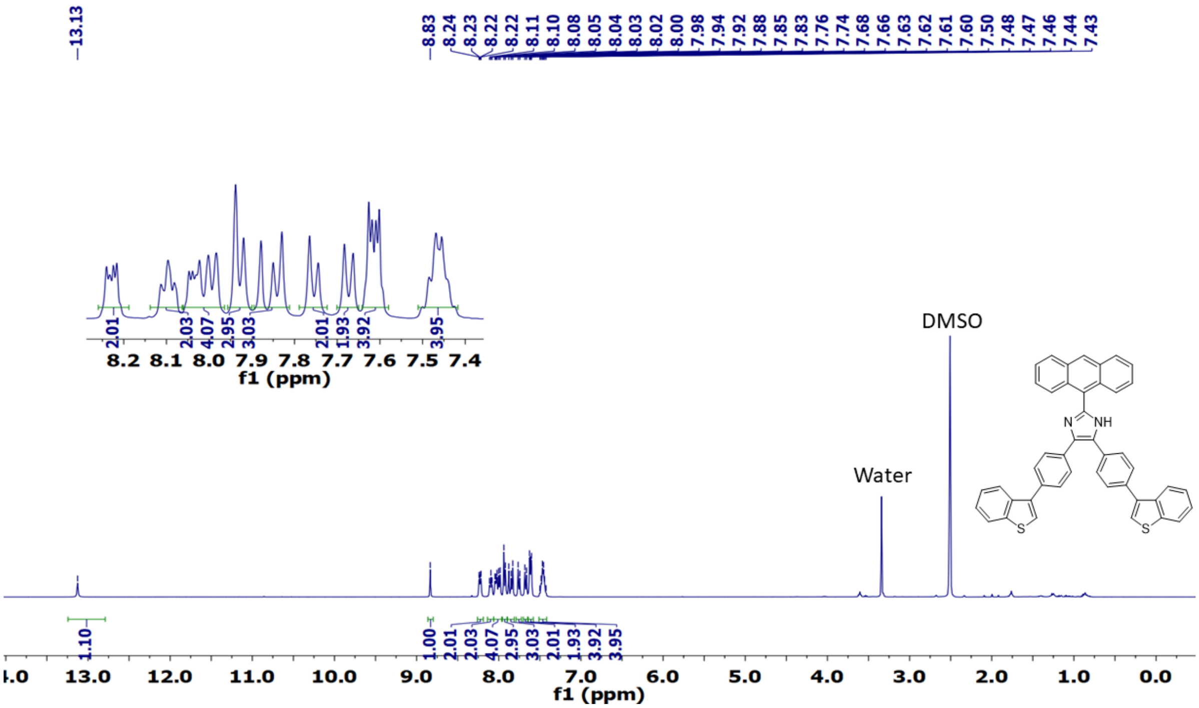

Fig. S-5 ^{1}H NMR (400 MHz, DMSO-d_6) spectrum of compound **5b**.

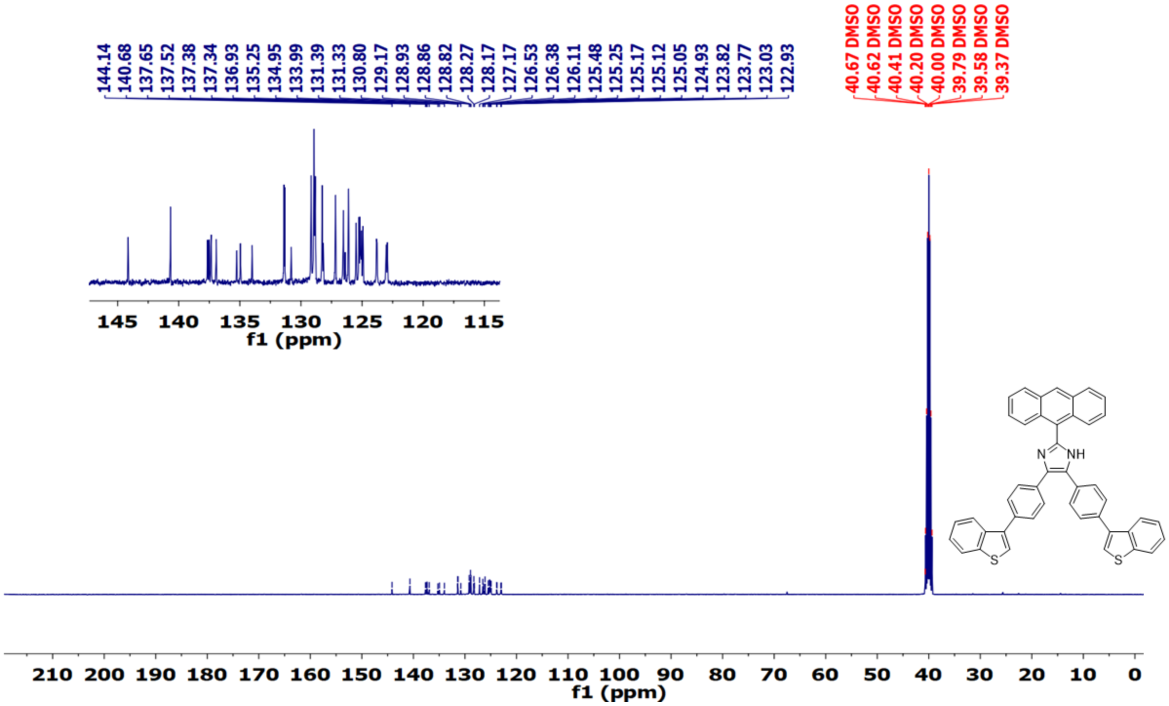

Fig. S-6 ^{13}C NMR (100 MHz, DMSO-d_6) spectrum of compound **5b**.

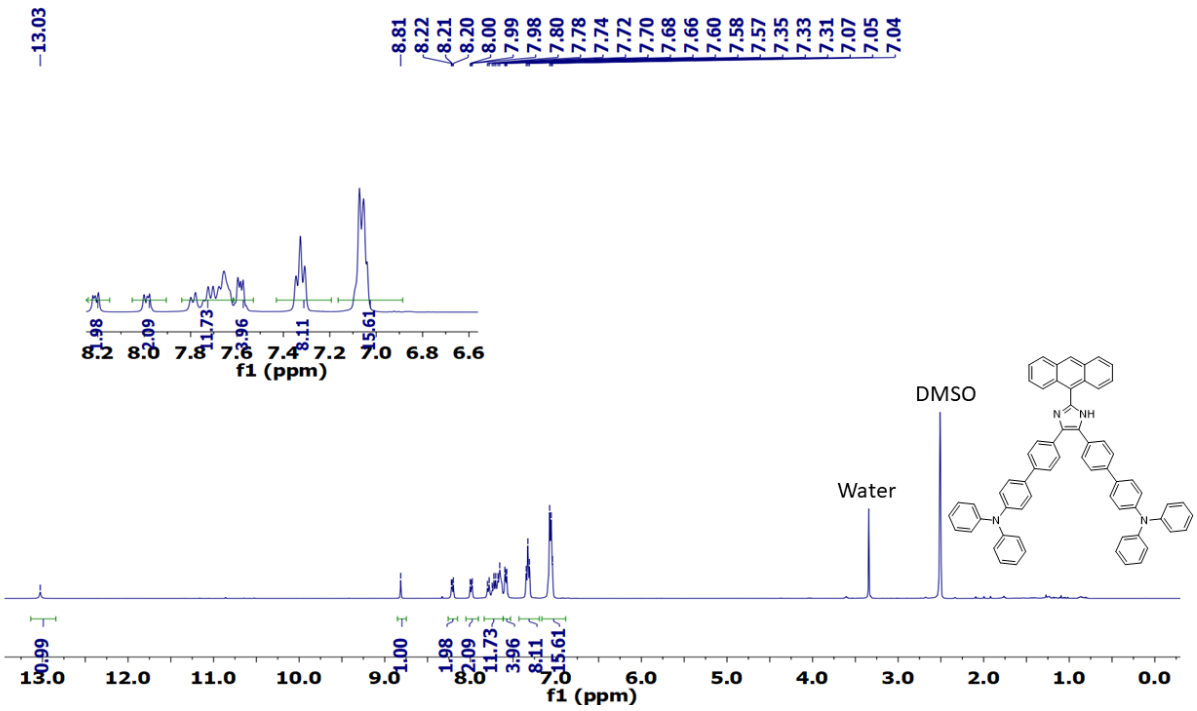

Fig. S-7 ^{1}H NMR (400 MHz, DMSO-d_6) spectrum of compound **5c**.

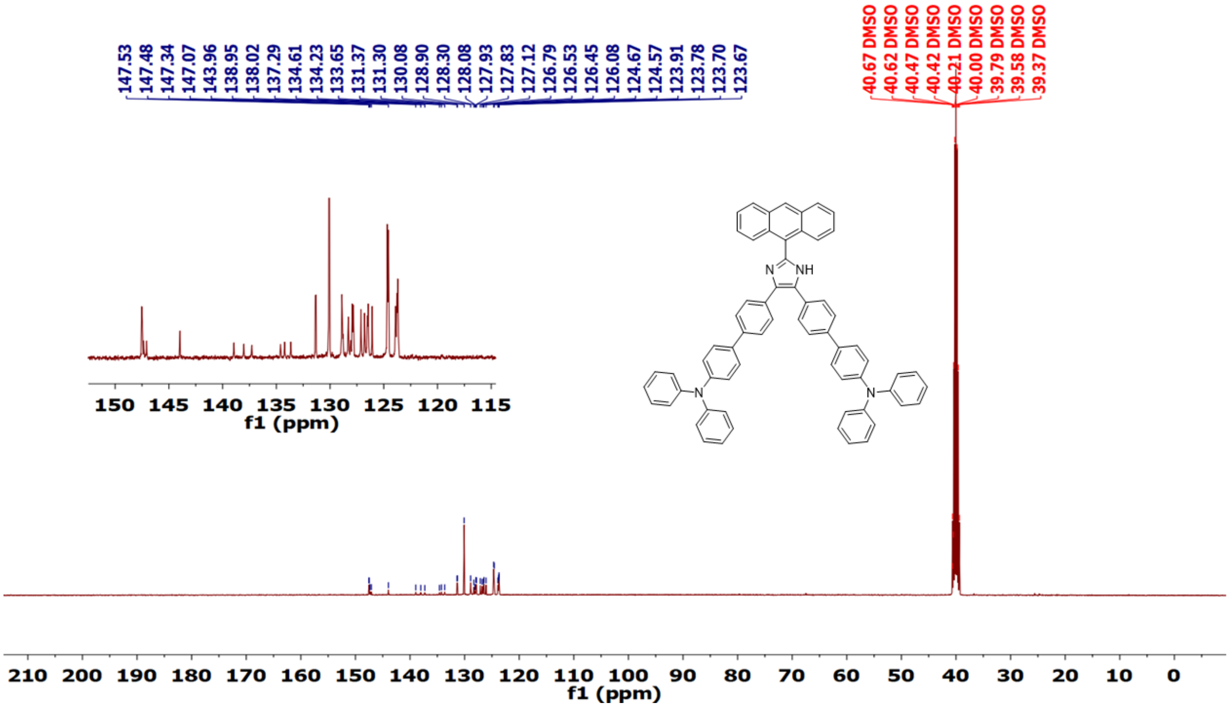

Fig. S-8 ^{13}C NMR (100 MHz, DMSO-d_6) spectrum of compound **5c**.

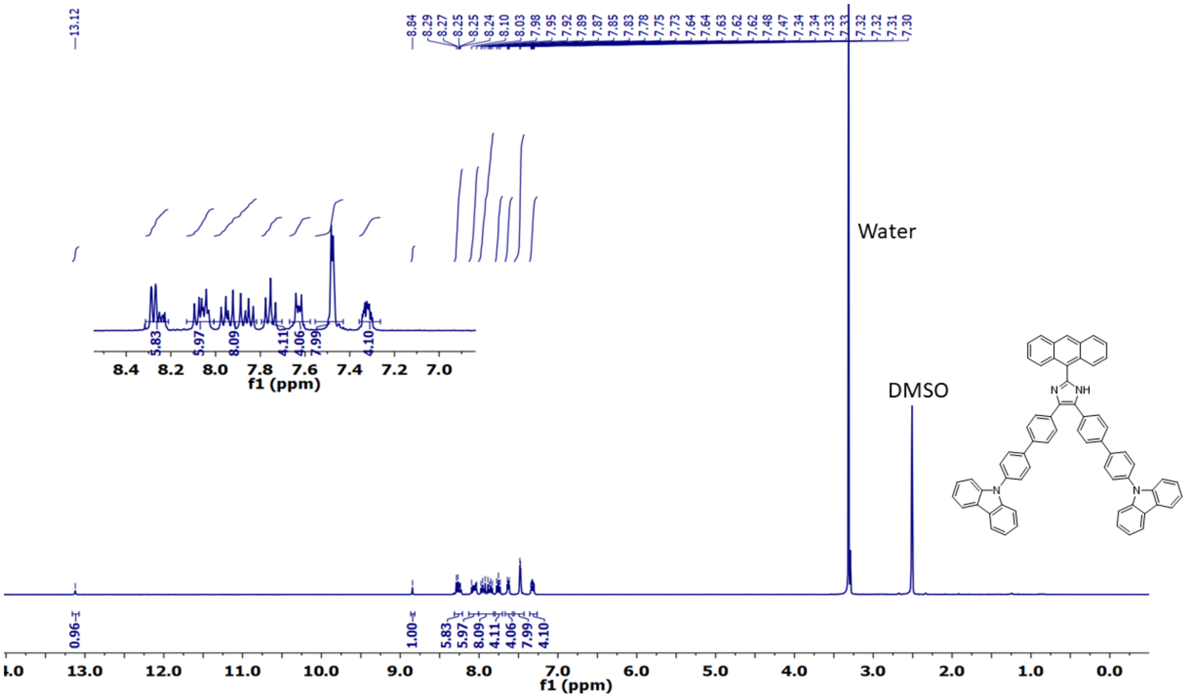

Fig. S-9 ^{1}H NMR (400 MHz, DMSO-d_6) spectrum of compound **5d**.

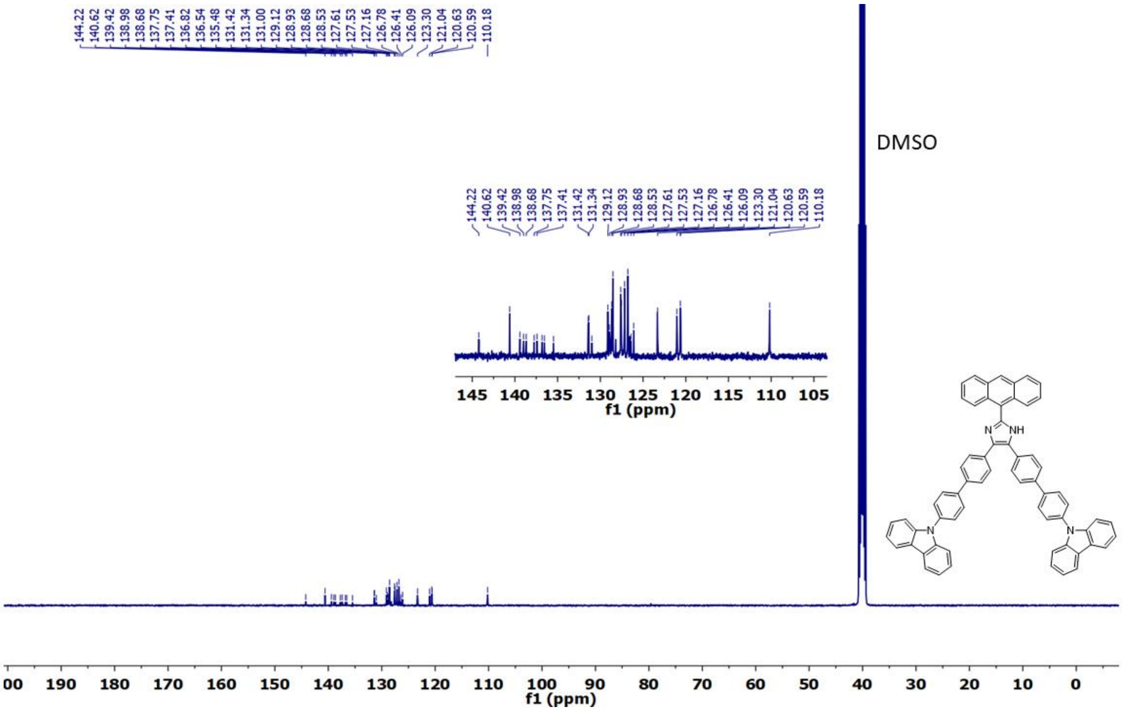

Fig. S-10 ^{13}C NMR (100 MHz, DMSO-*d6*) spectrum of compound **5d**.

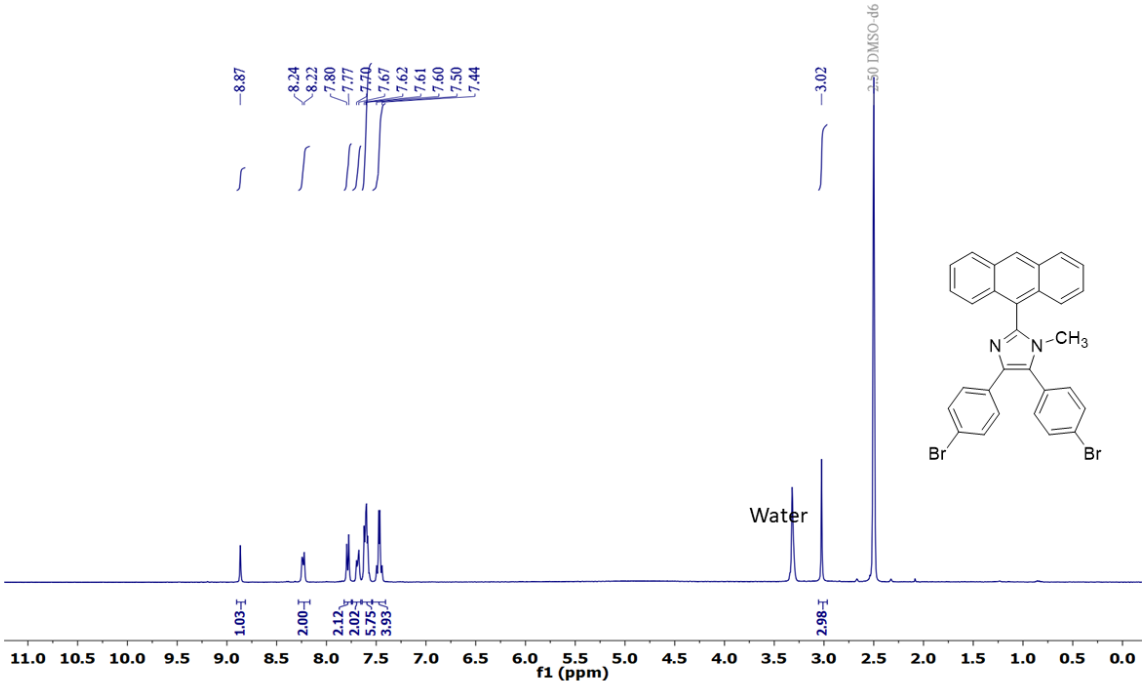

Fig. S-11 ^{1}H NMR (400 MHz, DMSO-d6) spectrum of compound **6**.

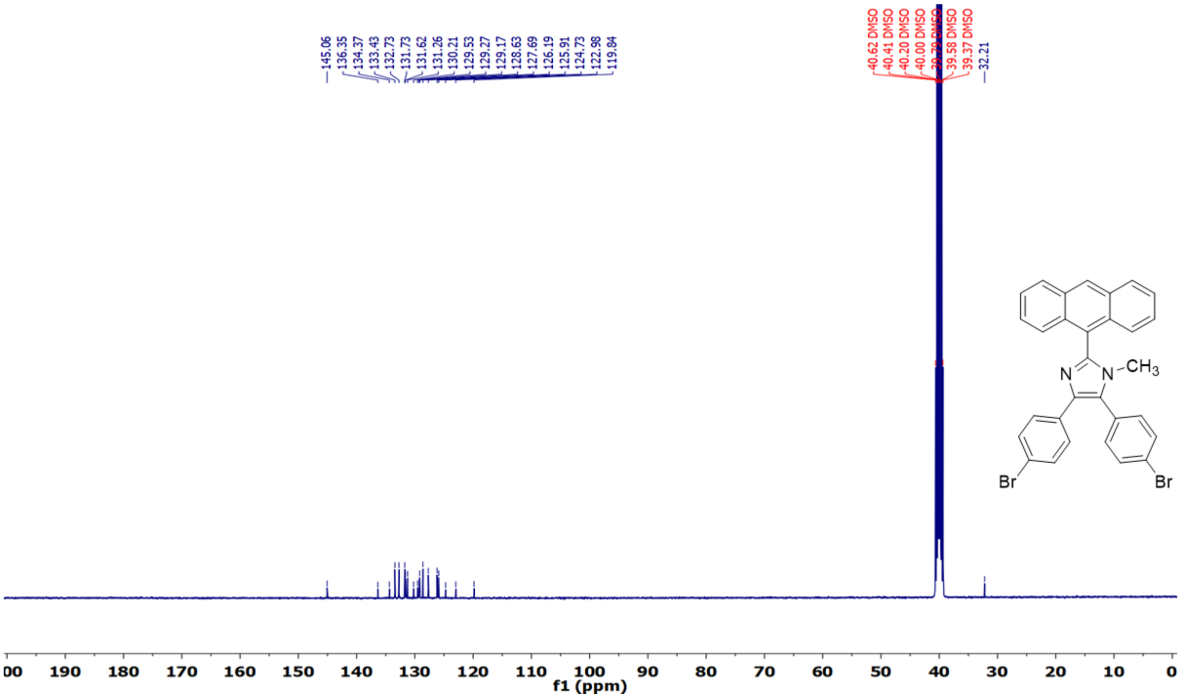

Fig. S-11 ^{13}C NMR (100 MHz, DMSO-d6) spectrum of compound **6**.

3. ^{1}H NMR Titration Result of **3** with TBAF

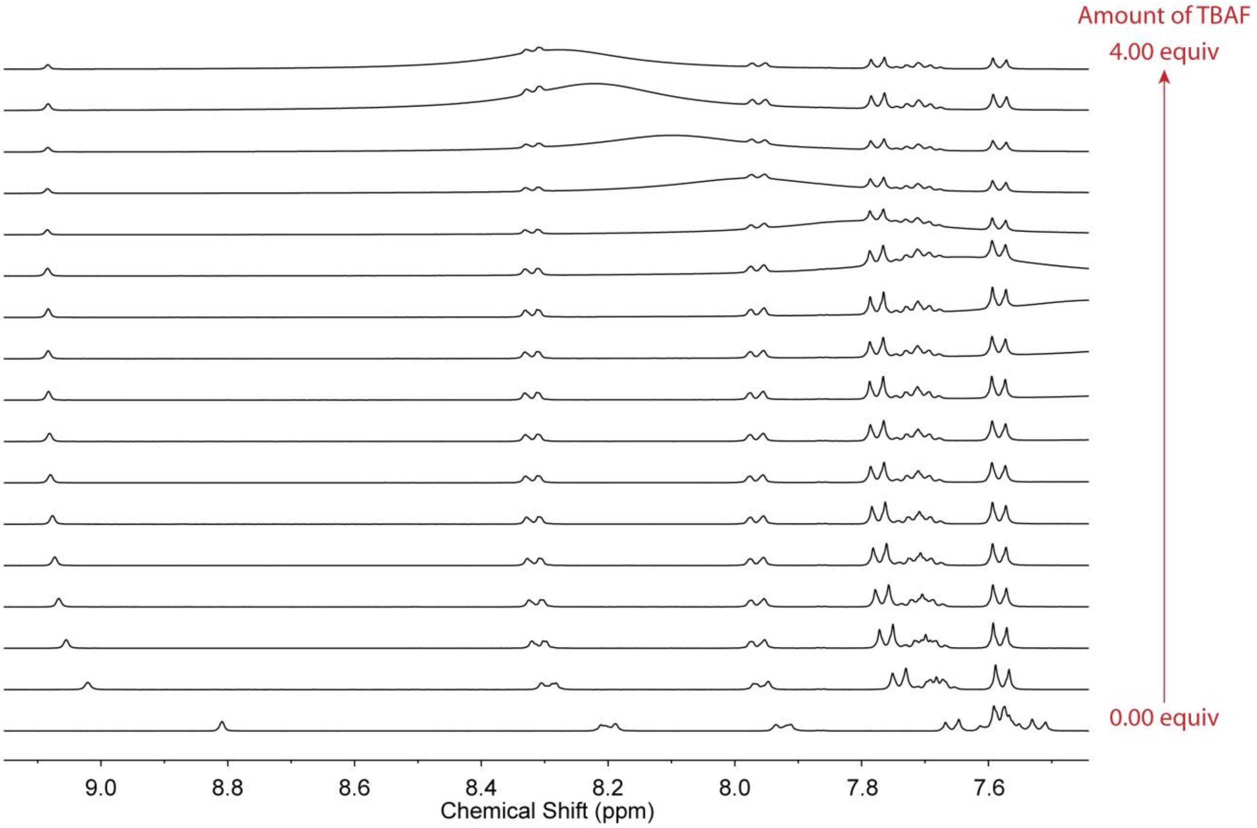

Fig. S-12 ^{1}H NMR (300 MHz, DMSO-d_6) spectra monitoring the titration f compound **3** (3.61mM) with TBAF from 0.00 to 4.00 mole equiv (addition of 0.25 mole equiv in each step).

3. High-Resolution Mass Spectra for Compounds **3, 5a-d,** and **6**

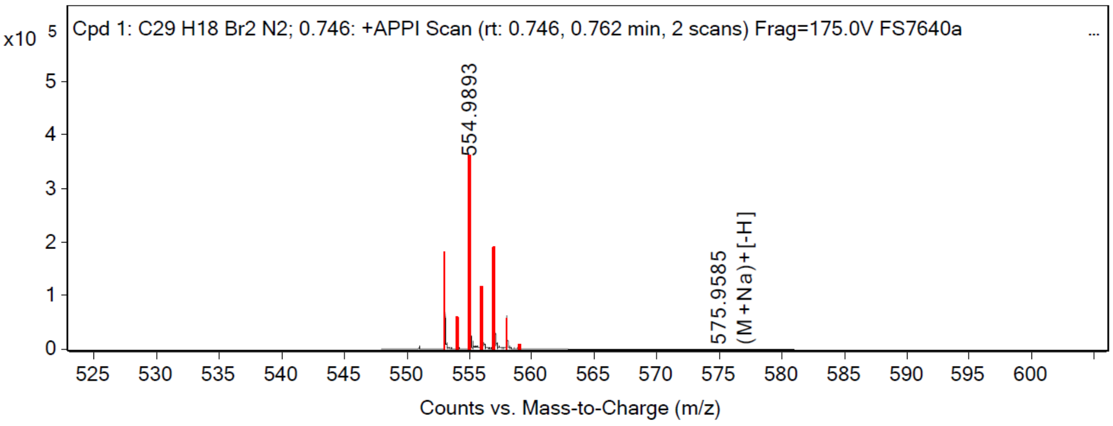

MS Spectrum Peak List

Obs. *m/z*	Charge	Abund	Ion/Isotope	Tgt Mass Error (ppm)
552.9977	1	170500.75	(M+H)+	-12.2
554.0014	1	53183.25	(M+H)+	-12.98
554.9893		362148.43		
554.9964	1	361546	(M+H)+	-13.11
555.9995	1	114515.75	(M+H)+	-13.04
556.9951	1	169493.25	(M+H)+	-13.35
557.9986	1	61632.75	(M+H)+	-14.69
559.0006	1	9767.56	(M+H)+	-12.85
560.0017	1	1161.15	(M+H)+	-9.13
575.9585	1	363.1	(M+Na)+[-H]	8.16

--- End Of Report ---

Fig. S-13 High-resolution mass spectrum (LC-TOF, positive mode) of compound **3.**

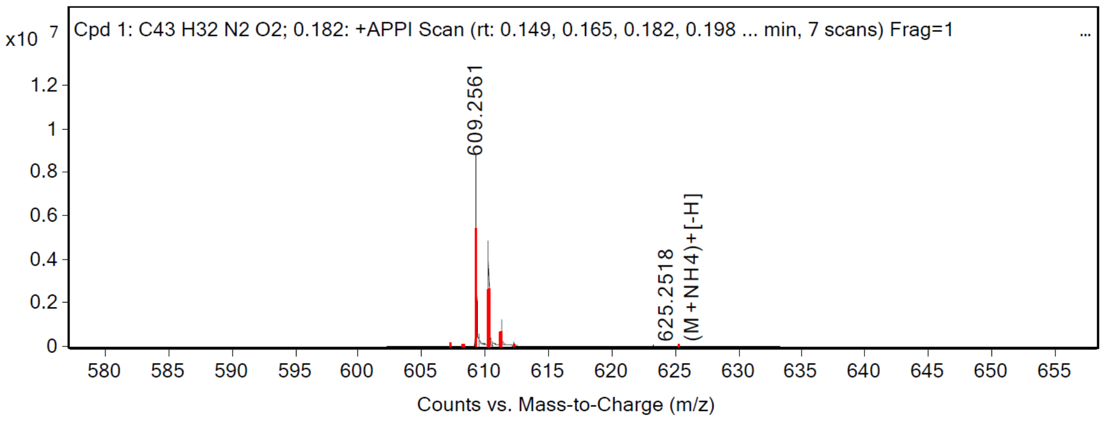

MS Spectrum Peak List

Obs. *m/z*	Charge	Abund	Ion/Isotope	Tgt Mass Error (ppm)
607.241	1	138680.44	M+[-H]	-4.94
608.2449	1	62663.53	M+[-H]	-6.02
609.2565	1	5282727	(M+H)+	-4.62
610.2593	1	2684005.25	(M+H)+	-3.9
611.2625	1	685582.44	(M+H)+	-3.86
612.2661	1	107463.72	(M+H)+	-4.67
613.2684	1	13322.15	(M+H)+	-3.39
625.2518	1	51605.78	(M+NH4)+[-H]	32.88
626.2543	1	22719.76	(M+NH4)+[-H]	34.07
627.2557	1	6610.25	(M+NH4)+[-H]	36.83

--- End Of Report ---

Fig. S-14 High-resolution mass spectrum (LC-TOF, positive mode) of compound **5a.**

MS Zoomed Spectrum

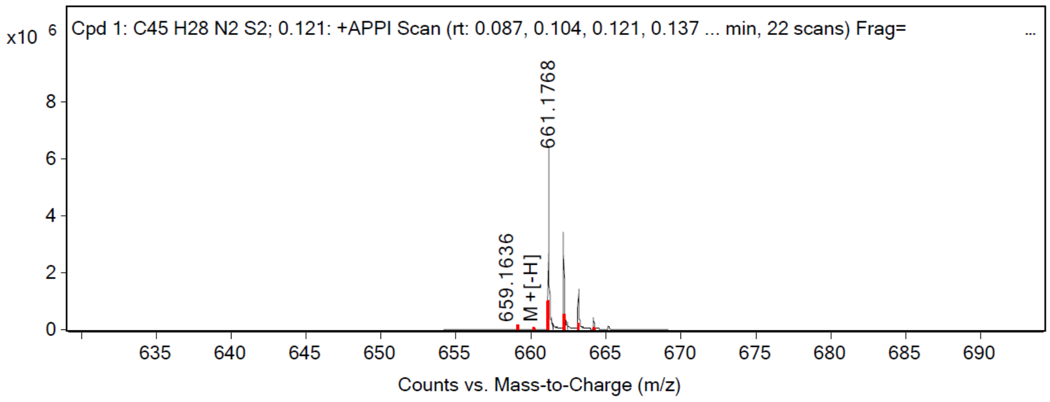

MS Spectrum Peak List

Obs. *m/z*	Charge	Abund	Ion/Isotope	Tgt Mass Error (ppm)
659.1636	1	132396.3	M+[-H]	-3.98
660.1668	1	67889.5	M+[-H]	-3.9
661.1768		6437437.58		
661.1785	1	1022435.75	(M+H)+	-2.8
662.1822	1	522301.31	(M+H)+	-3.61
663.1819	1	217120.09	(M+H)+	-4.81
664.1814	1	58764.23	(M+H)+	-3.66

--- End Of Report ---

Fig. S-15 High-resolution mass spectrum (LC-TOF, positive mode) of compound **5b.**

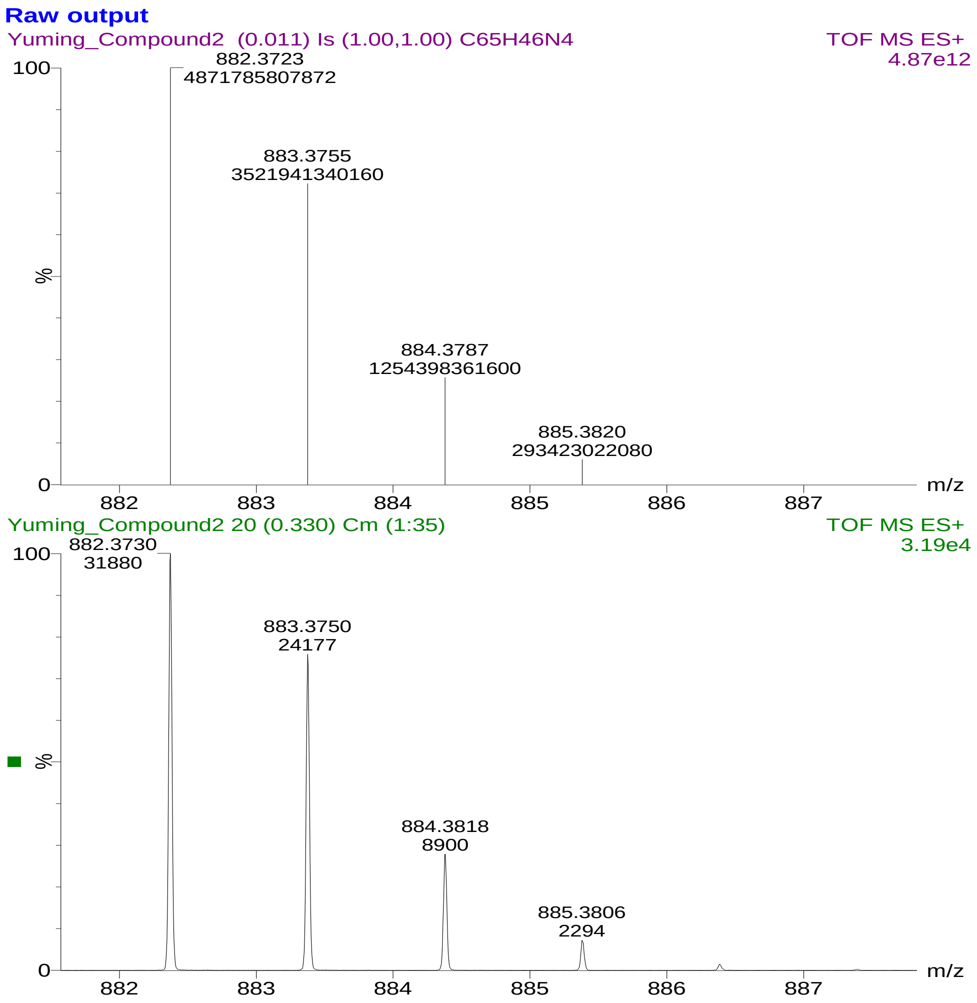

Fig. S-16 High-resolution mass spectrum (positive mode) of compound **5c** mesured using cyclic IMS cyclic ion mobility mass spectrometer (Wilmslow, UK)

MS Zoomed Spectrum

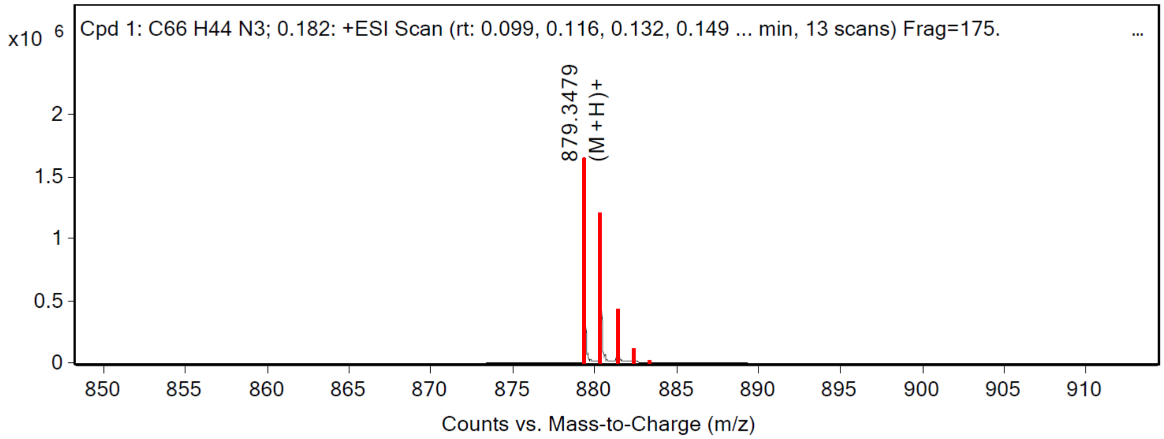

MS Spectrum Peak List

m/z	Calc *m/z*	Diff(ppm)	z	Abund	Ion
878.3338	878.353	21.87	1	530.95	M+
879.3479	879.3608	14.69		1637209.94	
879.3479	879.3608	14.69	1	1637432	(M+H)+
880.3513	880.3641	14.48	1	1197674.88	(M+H)+
881.3551	881.3674	13.88	1	422578.66	(M+H)+
882.3577	882.3706	14.66	1	95695.16	(M+H)+
883.3606	883.3739	15.09	1	13954.7	(M+H)+
884.3604	884.3772	18.97	1	2051.93	(M+H)+

--- End Of Report ---

Fig. S-17 High-resolution mass spectrum (LC-TOF, positive mode) of compound **5d.**

MS Zoomed Spectrum

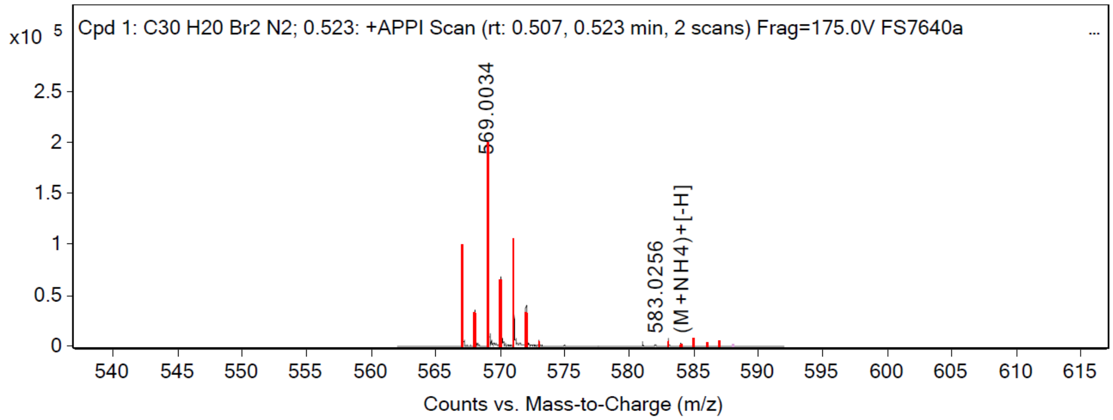

MS Spectrum Peak List

Obs. *m/z*	Charge	Abund	Ion/Isotope	Tgt Mass Error (ppm)
567.0119	1	96117.25	(M+H)+	-9.33
568.0158	1	36106.31	(M+H)+	-10.57
569.0101	1	191898.5	(M+H)+	-9.39
570.0134	1	68222	(M+H)+	-9.71
571.0087	1	102219.5	(M+H)+	-9.42
572.0122	1	39948.13	(M+H)+	-10.75
573.0143	1	6071.34	(M+H)+	-9.07
583.0256	1	8260.83	(M+NH4)+[-H]	-0.54
584.0285	1	2941.85	(M+NH4)+[-H]	-0.03
585.0227	1	3465.42	(M+NH4)+[-H]	1.39

--- End Of Report ---

Fig. S-18 High-resolution mass spectrum (LC-TOF, positive mode) of compound **6.**

5. DFT Calculations of Compound **3**

The molecular structure of ADPI **3** was optimized at the M06-2X/Def2-SVP level of theory with the solvent effect (CH_2Cl_2, SMD) considered. The optimized geometry was validated by frequency calculations at the same level of theory, which showed no presence of any imaginary frequencies. All calculations were done using the Gaussian 16 software package (Gaussian 16, Revision C.01, M. J. Frisch et al., Gaussian, Inc., Wallingford CT, 2016).

Cartesian coordinates of optimized **3** (*E* = -6371.907813 Hartree, dipole moment = 6.420254 Debye)

H	6.27687300	-3.16143300	-3.33578400
C	5.71657400	-2.53054500	-2.64388800
H	7.46012100	-1.61477700	-1.80009800
C	6.37008900	-1.68081100	-1.79896800
C	3.56067300	-1.81801900	-1.77013300
C	5.64292800	-0.84793100	-0.88616800
C	4.29051300	-2.59391500	-2.62725700
C	4.20554500	-0.91555600	-0.85794600
C	6.31184400	0.03722300	-0.03665000
H	3.77722000	-3.27319000	-3.31005600
H	2.47440300	-1.89087100	-1.76265600
C	5.61717200	0.85021200	0.86226400
H	7.40266600	0.09128500	-0.07298500
C	6.31676100	1.74208100	1.74031900
C	4.18052400	0.78348100	0.91829900
H	2.43165800	1.53051000	2.00700400
C	3.49220200	-0.08515000	0.03865400
C	5.64126300	2.51187700	2.64265700
H	7.40580300	1.78324700	1.67093200
H	6.18143800	3.18631400	3.30886300
C	4.21915700	2.42287200	2.72949300
H	3.68931200	3.01936600	3.47410000
C	3.51498100	1.59234300	1.90260500
C	2.01156400	-0.11184900	0.04194900
N	1.25759800	1.00388500	-0.14999800
H	1.61357100	1.94786900	-0.28023800
N	1.23951700	-1.16521500	0.20289200
C	-0.05499700	-0.72326900	0.11811500
C	-0.06659900	0.65220000	-0.09277200
C	-1.13087900	1.66054100	-0.21630000
C	-3.11806600	3.60818100	-0.43808300
C	-1.00699800	2.70544000	-1.14348200
C	-2.26704000	1.61722900	0.60570900
C	-3.26406300	2.58177300	0.49514500
C	-1.99375700	3.68184700	-1.25691100
H	-0.13538800	2.75223500	-1.79978400

H	-2.36935800	0.82372400	1.34829600
H	-4.14241900	2.53973600	1.14017100
H	-1.88900000	4.48744900	-1.98404300
C	-1.17348100	-1.67965200	0.21772900
C	-3.23064300	-3.56089700	0.41520400
C	-2.39306100	-1.47844500	-0.44513500
C	-1.00071900	-2.85556200	0.96238400
C	-2.02179200	-3.79619700	1.06721300
C	-3.42435900	-2.40915900	-0.34539400
H	-2.54047300	-0.59202300	-1.06417200
H	-0.04815300	-3.03015100	1.46431200
H	-1.87633000	-4.70392200	1.65379900
H	-4.36709900	-2.24235600	-0.86746900
Br	-4.46905300	4.92886900	-0.59264900
Br	-4.63085200	-4.83417700	0.55401000

6. Crystallographic Data and Detailed Refinements

*6.1 Crystallographic Data and Detailed Refinements for **3***

Table S-1 Crystal data and structure refinement

Identification code	AM-BA
Empirical formula	$C_{29.9}H_{19.4}Br_2Cl_{0.2}N_{2.4}$
Formula weight	579.12
Temperature/K	100(2)
Crystal system	triclinic
Space group	*P*-1
a/Å	14.37750(10)
b/Å	15.7779(2)
c/Å	23.0024(2)
α/°	84.7150(10)
β/°	78.8930(10)
γ/°	72.8160(10)
Volume/Å^3	4888.27(9)
Z	8
ρ_{calc}g/cm^3	1.574
μ/mm^{-1}	4.573
F(000)	2312.0
Crystal size/mm^3	0.064 × 0.052 × 0.036
Radiation	Cu *K*α (λ = 1.54184)
2θ range for data collection/°	5.868 to 158.75
Index ranges	$-18 \leq h \leq 17$, $-20 \leq k \leq 20$, $-29 \leq l \leq 29$
Reflections collected	111890
Independent reflections	20384 [R_{int} = 0.0539, R_{sigma} = 0.0346]
Data/restraints/parameters	20384/51/1289
Goodness-of-fit on F^2	1.055
Final *R* indexes [I>=2σ (I)]	R_1 = 0.0455, wR_2 = 0.1087
Final *R* indexes [all data]	R_1 = 0.0536, wR_2 = 0.1139
Largest diff. peak/hole / e Å^{-3}	1.58/-1.29

Table S-2 Fractional Atomic Coordinates (×10^4) and Equivalent Isotropic Displacement Parameters (Å^2×10^3). U_{eq} is defined as 1/3 of the trace of the orthogonalized U_{IJ} tensor.

**Atom**	***x***	***y***	***z***	**U(eq)**
Br1	-2763.3(3)	7642.4(3)	4597.9(2)	33.25(9)
Br2	3904.4(3)	4406.7(3)	4896.5(2)	42.67(11)
N1	-667.5(19)	8055.3(18)	6971.0(12)	20.1(5)
N2	875(2)	7342.5(18)	7065.3(13)	21.4(6)
C1	-232(2)	7507(2)	6494.2(14)	19.8(6)
C2	727(2)	7066(2)	6546.7(14)	18.8(6)
C3	17(2)	7930(2)	7307.6(15)	21.8(6)
C4	-808(2)	7494(2)	6035.2(15)	19.3(6)
C5	-1837(2)	7754(2)	6178.4(16)	26.2(7)
C6	-2415(3)	7783(3)	5755.3(17)	31.3(8)
C7	-1966(3)	7556(2)	5186.8(15)	24.3(7)
C8	-948(2)	7286(2)	5027.7(16)	23.9(7)
C9	-374(2)	7254(2)	5453.8(15)	21.8(6)
C10	1512(2)	6410(2)	6170.7(15)	21.0(6)
C11	1326(2)	5643(2)	6018.4(16)	23.3(7)
C12	2040(3)	5045(2)	5646.3(16)	26.9(7)
C13	2938(3)	5212(2)	5428.2(16)	27.9(7)
C14	3148(3)	5957(3)	5578.1(18)	31.9(8)
C15	2422(3)	6559(2)	5950.2(17)	27.9(7)
C16	-183(2)	8313(2)	7898.6(16)	25.4(7)
C17	246(3)	8971(2)	7998.5(17)	28.2(8)
C18	809(3)	9364(2)	7528(2)	33.9(9)
C19	1202(3)	10014(3)	7639(2)	43.7(11)
C20	1051(3)	10302(3)	8226(2)	47.3(12)
C21	501(3)	9964(3)	8677(2)	45.5(11)
C22	60(3)	9296(3)	8585.4(19)	35.3(9)
C23	-578(3)	8994(3)	9029.8(18)	37.1(9)
C24	-1064(3)	8397(3)	8931.8(17)	32.6(8)
C25	-1781(3)	8137(3)	9377.0(18)	38.7(10)
C26	-2254(3)	7565(3)	9265.3(18)	40.9(10)
C27	-2020(3)	7173(3)	8704.7(18)	39.7(9)
C28	-1329(3)	7396(3)	8264.2(17)	31.5(8)
C29	-850(3)	8032(2)	8351.5(16)	27.6(7)
Br3	4155.3(4)	9721.6(3)	5494.4(2)	42.43(11)
Br4	8833.3(3)	5381.0(3)	7412.2(2)	34.5(1)
N3	2796.3(19)	6466.6(18)	7380.5(13)	21.2(5)
N4	3913(2)	5328.1(19)	7707.5(13)	20.9(5)

Atom	*x*	*y*	*z*	U(eq)
C30	3726(2)	6588(2)	7195.3(15)	22.5(7)
C31	4424(2)	5894(2)	7407.3(15)	21.7(6)
C32	2943(2)	5696(2)	7682.5(15)	20.3(6)
C33	3849(2)	7339(2)	6790.7(15)	23.4(7)
C34	3110(3)	8149(2)	6844.4(16)	26.3(7)
C35	3185(3)	8851(3)	6450.9(17)	30.4(8)
C36	4001(3)	8745(3)	6008.5(17)	32.7(8)
C37	4736(3)	7940(3)	5939.2(17)	30.3(8)
C38	4656(3)	7239(2)	6328.3(16)	26.3(7)
C39	5490(2)	5763(2)	7380.0(15)	22.0(6)
C40	5825(3)	6486(2)	7469.1(16)	25.9(7)
C41	6815(3)	6385(2)	7459.5(16)	25.8(7)
C42	7485(2)	5545(2)	7368.5(16)	25.6(7)
C43	7181(2)	4824(2)	7274.4(15)	23.3(7)
C44	6184(2)	4939(2)	7273.8(15)	23.3(7)
C45	2156(2)	5267(2)	7919.5(15)	21.3(6)
C46	2099(2)	4562(2)	7607.0(15)	21.9(6)
C47	2777(2)	4240(2)	7081.4(16)	24.4(7)
C48	2685(3)	3565(2)	6788.7(17)	28.9(8)
C49	1913(3)	3162(3)	7000.3(18)	32.9(8)
C50	1245(3)	3462(3)	7488.9(19)	33.5(8)
C51	1302(3)	4172(2)	7810.5(16)	26.3(7)
C52	615(3)	4494(3)	8310.5(17)	29.2(8)
C53	672(3)	5185(2)	8623.3(16)	27.8(7)
C54	-16(3)	5490(3)	9149.8(18)	35.4(9)
C55	59(3)	6160(3)	9451.1(17)	36.1(9)
C56	806(3)	6584(3)	9239.3(17)	34.9(9)
C57	1485(3)	6308(2)	8746.9(17)	28.6(7)
C58	1449(2)	5591(2)	8419.9(15)	23.6(7)
Br5	4538.1(3)	7330.4(3)	9908.2(2)	41.46(11)
Br6	10638.4(3)	2310.4(3)	9147.2(2)	36.14(10)
N5	4973.5(19)	3698.8(18)	8281.8(12)	19.6(5)
N6	6414(2)	2717.1(18)	8005.8(12)	19.4(5)
C59	5694(2)	3919(2)	8508.6(14)	19.3(6)
C60	6595(2)	3305(2)	8349.9(14)	19.5(6)
C61	5439(2)	2964(2)	7983.2(14)	19.8(6)
C62	5427(2)	4743(2)	8839.9(14)	20.7(6)
C63	4471(3)	5060(2)	9159.4(16)	27.1(7)
C64	4189(3)	5835(3)	9469.3(17)	31.4(8)
C65	4886(3)	6284(2)	9461.7(16)	28.3(7)
C66	5833(3)	5993(2)	9147.5(16)	29.1(8)

Atom	*x*	*y*	*z*	U(eq)
C67	6095(3)	5229(2)	8829.9(16)	25.8(7)
C68	7571(2)	3139(2)	8518.7(15)	20.9(6)
C69	7667(3)	3398(2)	9061.0(16)	27.0(7)
C70	8573(3)	3182(3)	9243.6(16)	29.5(8)
C71	9395(3)	2671(2)	8883.5(17)	27.3(7)
C72	9331(3)	2405(2)	8342.0(16)	25.5(7)
C73	8420(2)	2653(2)	8159.1(15)	21.3(6)
C74	4960(2)	2393(2)	7743.0(15)	20.8(6)
C75	5017(2)	2338(2)	7129.8(16)	23.9(7)
C76	5486(3)	2859(2)	6694.8(16)	27.1(7)
C77	5513(3)	2802(3)	6103.1(17)	33.7(8)
C78	5096(3)	2186(3)	5907.6(19)	40.5(11)
C79	4653(3)	1674(3)	6302(2)	38.1(10)
C80	4574(2)	1741(2)	6922.7(18)	28.6(8)
C81	4065(3)	1259(2)	7336(2)	35.9(9)
C82	3983(3)	1323(2)	7940(2)	33.1(9)
C83	3454(3)	835(3)	8365(2)	43.8(11)
C84	3402(3)	885(3)	8955(3)	49.4(12)
C85	3908(3)	1409(3)	9165(2)	45.1(11)
C86	4421(3)	1890(3)	8779.7(19)	33.4(8)
C87	4461(2)	1883(2)	8155.9(17)	25.6(7)
Br7	8994.7(3)	5069.6(2)	5730.4(2)	31.17(9)
Br8	12547.3(3)	-918.2(3)	4573.7(2)	29.65(9)
N7	7811.8(19)	1388.4(17)	7221.4(12)	18.2(5)
N8	8579.5(19)	31.3(18)	6922.9(12)	18.3(5)
C88	8586(2)	1420(2)	6764.0(14)	18.7(6)
C89	9083(2)	571(2)	6572.9(14)	18.9(6)
C90	7837(2)	543(2)	7308.0(14)	18.9(6)
C91	8723(2)	2288(2)	6510.0(14)	19.6(6)
C92	8096(2)	2786(2)	6135.2(15)	23.8(7)
C93	8178(3)	3610(2)	5895.2(16)	25.4(7)
C94	8890(2)	3939(2)	6047.7(15)	21.2(6)
C95	9519(2)	3465(2)	6420.7(15)	21.8(6)
C96	9438(2)	2635(2)	6648.2(15)	21.3(6)
C97	9909(2)	227(2)	6097.7(14)	19.3(6)
C98	10643(3)	652(2)	5905.8(17)	27.2(7)
C99	11423(3)	322(2)	5448.7(16)	27.3(7)
C100	11480(2)	-457(2)	5191.2(15)	23.3(7)
C101	10762(3)	-895(2)	5374.8(16)	27.4(7)
C102	9974(3)	-546(2)	5819.5(15)	24.4(7)
C103	7156(2)	182(2)	7757.4(15)	20.4(6)

Atom	*x*	*y*	*z*	U(eq)
C104	6544(2)	-258(2)	7571.7(15)	21.2(6)
C105	6483(2)	-300(2)	6963.8(16)	24.0(7)
C106	5849(3)	-704(2)	6808.7(17)	28.1(7)
C107	5258(3)	-1110(2)	7244.9(19)	33.3(8)
C108	5298(3)	-1082(2)	7828.3(19)	31.6(8)
C109	5914(2)	-638(2)	8016.8(17)	25.4(7)
C110	5891(3)	-536(2)	8613.1(17)	28.0(7)
C111	6446(3)	-55(2)	8796.8(16)	25.7(7)
C112	6369(3)	110(3)	9408.9(17)	32.3(8)
C113	6908(3)	586(3)	9579.5(17)	33.6(8)
C114	7586(3)	914(2)	9156.9(16)	30.8(8)
C115	7697(3)	763(2)	8569.2(16)	24.7(7)
C116	7111(2)	298(2)	8363.6(15)	21.2(6)
Cl1	3340(4)	7679(3)	8394.6(18)	84.1(19)
Cl2	2026(4)	8255(4)	9494(3)	50.7(12)
C119	2873(11)	8553(8)	8898(6)	65(4)
N9	2256(3)	4816(3)	10070(2)	53.5(10)
C117	2350(3)	4329(3)	9710(2)	39.2(9)
C118	2497(3)	3697(3)	9250(2)	42.7(10)
N10	2829(9)	8160(8)	8592(5)	89(3)
C120	2382(9)	8228(9)	9079(5)	76(3)
C121	1735(12)	8411(14)	9628(6)	67(4)

Table S-3 Selected Bond Distances (Å)

Atom	Atom	Length/Å	Atom	Atom	Length/Å
Br1	C7	1.908(3)	N5	C61	1.332(4)
Br2	C13	1.905(3)	N6	C60	1.383(4)
N1	C1	1.391(4)	N6	C61	1.349(4)
N1	C3	1.325(4)	C59	C60	1.376(4)
N2	C2	1.381(4)	C59	C62	1.481(4)
N2	C3	1.359(4)	C60	C68	1.471(4)
C1	C2	1.372(4)	C61	C74	1.483(4)
C1	C4	1.467(4)	C62	C63	1.397(5)
C2	C10	1.480(4)	C62	C67	1.390(5)
C3	C16	1.482(5)	C63	C64	1.387(5)
C4	C5	1.394(5)	C64	C65	1.386(5)
C4	C9	1.398(5)	C65	C66	1.378(5)
C5	C6	1.385(5)	C66	C67	1.385(5)

Atom	Atom	Length/Å	Atom	Atom	Length/Å
C6	C7	1.371(5)	C68	C69	1.391(5)
C7	C8	1.382(5)	C68	C73	1.392(4)
C8	C9	1.387(5)	C69	C70	1.383(5)
C10	C11	1.401(5)	C70	C71	1.384(5)
C10	C15	1.389(5)	C71	C72	1.378(5)
C11	C12	1.384(5)	C72	C73	1.387(5)
C12	C13	1.385(5)	C74	C75	1.406(5)
C13	C14	1.384(5)	C74	C87	1.414(5)
C14	C15	1.394(5)	C75	C76	1.431(5)
C16	C17	1.410(5)	C75	C80	1.439(5)
C16	C29	1.408(6)	C76	C77	1.365(5)
C17	C18	1.431(6)	C77	C78	1.426(6)
C17	C22	1.437(5)	C78	C79	1.353(7)
C18	C19	1.373(5)	C79	C80	1.421(6)
C19	C20	1.422(7)	C80	C81	1.396(6)
C20	C21	1.347(8)	C81	C82	1.381(6)
C21	C22	1.430(6)	C82	C83	1.432(6)
C22	C23	1.386(7)	C82	C87	1.440(5)
C23	C24	1.383(6)	C83	C84	1.353(7)
C24	C25	1.431(6)	C84	C85	1.418(7)
C24	C29	1.445(5)	C85	C86	1.364(6)
C25	C26	1.347(7)	C86	C87	1.426(6)
C26	C27	1.420(6)	Br7	C94	1.900(3)
C27	C28	1.375(6)	Br8	C100	1.895(3)
C28	C29	1.421(5)	N7	C88	1.386(4)
Br3	C36	1.893(4)	N7	C90	1.322(4)
Br4	C42	1.900(3)	N8	C89	1.382(4)
N3	C30	1.386(4)	N8	C90	1.354(4)
N3	C32	1.325(4)	C88	C89	1.386(4)
N4	C31	1.375(5)	C88	C91	1.492(4)
N4	C32	1.353(4)	C89	C97	1.459(4)
C30	C31	1.374(5)	C90	C103	1.477(5)
C30	C33	1.469(5)	C91	C92	1.389(5)
C31	C39	1.473(4)	C91	C96	1.396(4)
C32	C45	1.477(5)	C92	C93	1.394(5)
C33	C34	1.397(5)	C93	C94	1.388(5)
C33	C38	1.396(5)	C94	C95	1.377(5)
C34	C35	1.381(5)	C95	C96	1.393(5)
C35	C36	1.377(6)	C97	C98	1.394(5)
C36	C37	1.392(6)	C97	C102	1.399(4)
C37	C38	1.374(5)	C98	C99	1.392(5)

Atom	Atom	Length/Å	Atom	Atom	Length/Å
C39	C40	1.408(5)	C99	C100	1.388(5)
C39	C44	1.394(5)	C100	C101	1.387(5)
C40	C41	1.381(5)	C101	C102	1.387(5)
C41	C42	1.395(5)	C103	C104	1.414(5)
C42	C43	1.380(5)	C103	C116	1.410(4)
C43	C44	1.391(5)	C104	C105	1.427(5)
C45	C46	1.410(5)	C104	C109	1.442(5)
C45	C58	1.404(5)	C105	C106	1.369(5)
C46	C47	1.427(5)	C106	C107	1.420(6)
C46	C51	1.437(5)	C107	C108	1.360(6)
C47	C48	1.361(5)	C108	C109	1.426(5)
C48	C49	1.424(6)	C109	C110	1.389(5)
C49	C50	1.350(6)	C110	C111	1.392(5)
C50	C51	1.428(5)	C111	C112	1.433(5)
C51	C52	1.389(5)	C111	C116	1.436(5)
C52	C53	1.390(5)	C112	C113	1.354(6)
C53	C54	1.429(5)	C113	C114	1.415(6)
C53	C58	1.429(5)	C114	C115	1.365(5)
C54	C55	1.357(6)	C115	C116	1.432(5)
C55	C56	1.414(6)	Cl1	C119	1.772(8)
C56	C57	1.356(5)	Cl2	C119	1.778(8)
C57	C58	1.433(5)	N9	C117	1.144(6)
Br5	C65	1.912(3)	C117	C118	1.463(6)
Br6	C71	1.908(3)	N10	C120	1.177(8)
N5	C59	1.386(4)	C120	C121	1.414(8)

Table S-4 Selected Bond Angles

Atom	Atom	Atom	Angle/°	Atom	Atom	Atom	Angle/°
C3	N1	C1	105.9(3)	N5	C59	C62	119.5(3)
C3	N2	C2	107.6(3)	C60	C59	N5	109.9(3)
N1	C1	C4	119.3(3)	C60	C59	C62	130.5(3)
C2	C1	N1	109.5(3)	N6	C60	C68	120.9(3)
C2	C1	C4	131.2(3)	C59	C60	N6	105.0(3)
N2	C2	C10	122.1(3)	C59	C60	C68	133.8(3)
C1	C2	N2	105.9(3)	N5	C61	N6	110.8(3)
C1	C2	C10	132.0(3)	N5	C61	C74	125.8(3)
N1	C3	N2	111.1(3)	N6	C61	C74	122.6(3)

Atom	Atom	Atom	Angle/°	Atom	Atom	Atom	Angle/°
N1	C3	C16	123.3(3)	C63	C62	C59	119.6(3)
N2	C3	C16	125.4(3)	C67	C62	C59	121.8(3)
C5	C4	C1	118.8(3)	C67	C62	C63	118.6(3)
C5	C4	C9	118.1(3)	C64	C63	C62	121.3(3)
C9	C4	C1	123.0(3)	C65	C64	C63	118.2(3)
C6	C5	C4	121.0(3)	C64	C65	Br5	119.4(3)
C7	C6	C5	119.4(3)	C66	C65	Br5	118.7(3)
C6	C7	Br1	119.3(3)	C66	C65	C64	121.9(3)
C6	C7	C8	121.5(3)	C65	C66	C67	119.0(3)
C8	C7	Br1	119.3(3)	C66	C67	C62	120.8(3)
C7	C8	C9	118.9(3)	C69	C68	C60	121.2(3)
C8	C9	C4	121.1(3)	C69	C68	C73	118.0(3)
C11	C10	C2	119.8(3)	C73	C68	C60	120.5(3)
C15	C10	C2	120.8(3)	C70	C69	C68	121.6(3)
C15	C10	C11	119.4(3)	C69	C70	C71	118.7(3)
C12	C11	C10	120.3(3)	C70	C71	Br6	119.4(3)
C11	C12	C13	119.2(3)	C72	C71	Br6	119.3(3)
C12	C13	Br2	119.2(3)	C72	C71	C70	121.3(3)
C14	C13	Br2	119.0(3)	C71	C72	C73	119.0(3)
C14	C13	C12	121.8(3)	C72	C73	C68	121.3(3)
C13	C14	C15	118.5(3)	C75	C74	C61	121.7(3)
C10	C15	C14	120.8(3)	C75	C74	C87	120.9(3)
C17	C16	C3	121.1(3)	C87	C74	C61	117.3(3)
C29	C16	C3	118.1(3)	C74	C75	C76	123.1(3)
C29	C16	C17	120.8(3)	C74	C75	C80	119.2(3)
C16	C17	C18	122.1(3)	C76	C75	C80	117.7(3)
C16	C17	C22	118.8(4)	C77	C76	C75	121.7(3)
C18	C17	C22	119.0(3)	C76	C77	C78	119.6(4)
C19	C18	C17	120.5(4)	C79	C78	C77	120.8(4)
C18	C19	C20	120.0(5)	C78	C79	C80	121.2(4)
C21	C20	C19	121.0(4)	C79	C80	C75	119.0(4)
C20	C21	C22	121.5(4)	C81	C80	C75	119.1(3)
C21	C22	C17	117.9(4)	C81	C80	C79	121.9(3)
C23	C22	C17	119.4(4)	C82	C81	C80	122.2(3)
C23	C22	C21	122.6(4)	C81	C82	C83	122.4(4)
C24	C23	C22	122.7(4)	C81	C82	C87	119.5(4)
C23	C24	C25	122.9(4)	C83	C82	C87	118.1(4)
C23	C24	C29	118.6(4)	C84	C83	C82	121.8(4)
C25	C24	C29	118.5(4)	C83	C84	C85	119.8(4)
C26	C25	C24	121.5(4)	C86	C85	C84	120.9(5)
C25	C26	C27	120.5(4)	C85	C86	C87	121.0(4)

Atom	Atom	Atom	Angle/°	Atom	Atom	Atom	Angle/°
C28	C27	C26	120.1(4)	C74	C87	C82	118.9(3)
C27	C28	C29	121.4(4)	C74	C87	C86	122.8(3)
C16	C29	C24	119.4(3)	C86	C87	C82	118.3(3)
C16	C29	C28	122.7(3)	C90	N7	C88	106.1(3)
C28	C29	C24	117.9(4)	C90	N8	C89	108.6(3)
C32	N3	C30	105.6(3)	N7	C88	C91	120.7(3)
C32	N4	C31	107.9(3)	C89	C88	N7	109.8(3)
N3	C30	C33	120.1(3)	C89	C88	C91	129.2(3)
C31	C30	N3	109.7(3)	N8	C89	C88	104.6(3)
C31	C30	C33	129.9(3)	N8	C89	C97	122.3(3)
N4	C31	C39	124.2(3)	C88	C89	C97	133.0(3)
C30	C31	N4	105.5(3)	N7	C90	N8	110.9(3)
C30	C31	C39	130.2(3)	N7	C90	C103	125.8(3)
N3	C32	N4	111.2(3)	N8	C90	C103	123.3(3)
N3	C32	C45	123.9(3)	C92	C91	C88	118.9(3)
N4	C32	C45	124.7(3)	C92	C91	C96	118.5(3)
C34	C33	C30	119.3(3)	C96	C91	C88	122.6(3)
C38	C33	C30	121.2(3)	C91	C92	C93	121.3(3)
C38	C33	C34	119.4(3)	C94	C93	C92	118.7(3)
C35	C34	C33	120.4(3)	C93	C94	Br7	118.9(3)
C36	C35	C34	119.1(4)	C95	C94	Br7	119.6(2)
C35	C36	Br3	119.7(3)	C95	C94	C93	121.5(3)
C35	C36	C37	121.4(3)	C94	C95	C96	119.0(3)
C37	C36	Br3	118.8(3)	C95	C96	C91	121.0(3)
C38	C37	C36	119.3(3)	C98	C97	C89	121.7(3)
C37	C38	C33	120.3(3)	C98	C97	C102	118.4(3)
C40	C39	C31	119.5(3)	C102	C97	C89	119.9(3)
C44	C39	C31	122.1(3)	C99	C98	C97	121.2(3)
C44	C39	C40	118.4(3)	C100	C99	C98	119.0(3)
C41	C40	C39	121.2(3)	C99	C100	Br8	119.4(3)
C40	C41	C42	118.8(3)	C101	C100	Br8	119.7(2)
C41	C42	Br4	119.1(3)	C101	C100	C99	120.9(3)
C43	C42	Br4	119.4(3)	C100	C101	C102	119.4(3)
C43	C42	C41	121.5(3)	C101	C102	C97	120.9(3)
C42	C43	C44	119.2(3)	C104	C103	C90	119.4(3)
C43	C44	C39	121.0(3)	C116	C103	C90	119.5(3)
C46	C45	C32	118.1(3)	C116	C103	C104	121.0(3)
C58	C45	C32	120.8(3)	C103	C104	C105	122.8(3)
C58	C45	C46	121.0(3)	C103	C104	C109	118.6(3)
C45	C46	C47	122.7(3)	C105	C104	C109	118.5(3)
C45	C46	C51	118.9(3)	C106	C105	C104	120.5(3)

Atom	Atom	Atom	Angle/°	Atom	Atom	Atom	Angle/°
C47	C46	C51	118.4(3)	C105	C106	C107	121.1(4)
C48	C47	C46	120.8(3)	C108	C107	C106	119.9(3)
C47	C48	C49	120.8(3)	C107	C108	C109	121.4(4)
C50	C49	C48	120.0(3)	C108	C109	C104	118.5(3)
C49	C50	C51	121.5(4)	C110	C109	C104	119.8(3)
C50	C51	C46	118.5(3)	C110	C109	C108	121.7(3)
C52	C51	C46	119.3(3)	C109	C110	C111	121.7(3)
C52	C51	C50	122.2(3)	C110	C111	C112	122.0(3)
C51	C52	C53	122.1(3)	C110	C111	C116	119.6(3)
C52	C53	C54	121.7(4)	C112	C111	C116	118.4(3)
C52	C53	C58	119.3(3)	C113	C112	C111	121.3(4)
C58	C53	C54	119.0(3)	C112	C113	C114	120.6(3)
C55	C54	C53	120.7(4)	C115	C114	C113	120.3(4)
C54	C55	C56	120.4(4)	C114	C115	C116	121.1(3)
C57	C56	C55	121.0(4)	C103	C116	C111	119.1(3)
C56	C57	C58	120.7(4)	C103	C116	C115	122.8(3)
C45	C58	C53	119.4(3)	C115	C116	C111	118.1(3)
C45	C58	C57	122.4(3)	Cl1	C119	Cl2	109.0(5)
C53	C58	C57	118.1(3)	N9	C117	C118	178.5(5)
C61	N5	C59	105.8(3)	N10	C120	C121	171.3(15)
C61	N6	C60	108.5(3)				

Table S-5 Selected Torsion Angles

A	B	C	D	Angle/°	A	B	C	D	Angle/°
Br1	C7	C8	C9	177.8(3)	Br5	C65	C66	C67	179.1(3)
Br2	C13	C14	C15	177.7(3)	Br6	C71	C72	C73	178.6(3)
N1	C1	C2	N2	0.6(4)	N5	C59	C60	N6	1.7(4)
N1	C1	C2	C10	179.8(3)	N5	C59	C60	C68	-171.3(3)
N1	C1	C4	C5	-23.3(5)	N5	C59	C62	C63	30.1(5)
N1	C1	C4	C9	155.1(3)	N5	C59	C62	C67	-148.2(3)
N1	C3	C16	C17	-112.9(4)	N5	C61	C74	C75	111.1(4)
N1	C3	C16	C29	64.1(5)	N5	C61	C74	C87	-70.5(4)
N2	C2	C10	C11	125.5(4)	N6	C60	C68	C69	-146.7(3)
N2	C2	C10	C15	-56.6(5)	N6	C60	C68	C73	28.0(5)
N2	C3	C16	C17	74.0(5)	N6	C61	C74	C75	-79.9(4)
N2	C3	C16	C29	-109.0(4)	N6	C61	C74	C87	98.4(4)
C1	N1	C3	N2	1.3(4)	C59	N5	C61	N6	-0.6(4)
C1	N1	C3	C16	-172.7(3)	C59	N5	C61	C74	169.5(3)

A	B	C	D	Angle/°	A	B	C	D	Angle/°
C1	C2	C10	C11	-53.7(5)	C59	C60	C68	C69	25.4(6)
C1	C2	C10	C15	124.3(4)	C59	C60	C68	C73	-159.9(4)
C1	C4	C5	C6	177.8(3)	C59	C62	C63	C64	-179.2(3)
C1	C4	C9	C8	-177.4(3)	C59	C62	C67	C66	-179.4(3)
C2	N2	C3	N1	-0.9(4)	C60	N6	C61	N5	1.7(4)
C2	N2	C3	C16	172.9(3)	C60	N6	C61	C74	-168.8(3)
C2	C1	C4	C5	158.2(4)	C60	C59	C62	C63	-153.3(4)
C2	C1	C4	C9	-23.4(6)	C60	C59	C62	C67	28.5(6)
C2	C10	C11	C12	177.2(3)	C60	C68	C69	C70	174.7(3)
C2	C10	C15	C14	-177.6(3)	C60	C68	C73	C72	-172.7(3)
C3	N1	C1	C2	-1.1(4)	C61	N5	C59	C60	-0.7(4)
C3	N1	C1	C4	-180.0(3)	C61	N5	C59	C62	176.6(3)
C3	N2	C2	C1	0.2(4)	C61	N6	C60	C59	-2.0(4)
C3	N2	C2	C10	-179.1(3)	C61	N6	C60	C68	172.1(3)
C3	C16	C17	C18	6.3(5)	C61	C74	C75	C76	-3.6(5)
C3	C16	C17	C22	-177.6(3)	C61	C74	C75	C80	177.2(3)
C3	C16	C29	C24	-179.7(3)	C61	C74	C87	C82	179.8(3)
C3	C16	C29	C28	0.5(5)	C61	C74	C87	C86	-1.1(5)
C4	C1	C2	N2	179.2(3)	C62	C59	C60	N6	-175.2(3)
C4	C1	C2	C10	-1.5(6)	C62	C59	C60	C68	11.8(7)
C4	C5	C6	C7	-0.3(6)	C62	C63	C64	C65	-0.9(6)
C5	C4	C9	C8	1.1(5)	C63	C62	C67	C66	2.4(5)
C5	C6	C7	Br1	-177.5(3)	C63	C64	C65	Br5	-177.7(3)
C5	C6	C7	C8	1.0(6)	C63	C64	C65	C66	1.4(6)
C6	C7	C8	C9	-0.6(5)	C64	C65	C66	C67	0.0(6)
C7	C8	C9	C4	-0.4(5)	C65	C66	C67	C62	-1.9(6)
C9	C4	C5	C6	-0.7(5)	C67	C62	C63	C64	-0.9(6)
C10	C11	C12	C13	0.2(5)	C68	C69	C70	C71	-2.1(6)
C11	C10	C15	C14	0.3(5)	C69	C68	C73	C72	2.2(5)
C11	C12	C13	Br2	-178.1(3)	C69	C70	C71	Br6	-176.6(3)
C11	C12	C13	C14	0.9(6)	C69	C70	C71	C72	2.4(6)
C12	C13	C14	C15	-1.3(6)	C70	C71	C72	C73	-0.5(6)
C13	C14	C15	C10	0.6(6)	C71	C72	C73	C68	-1.9(5)
C15	C10	C11	C12	-0.7(5)	C73	C68	C69	C70	-0.2(6)
C16	C17	C18	C19	178.9(3)	C74	C75	C76	C77	-178.8(3)
C16	C17	C22	C21	-180.0(3)	C74	C75	C80	C79	-178.6(3)
C16	C17	C22	C23	-3.6(5)	C74	C75	C80	C81	2.6(5)
C17	C16	C29	C24	-2.7(5)	C75	C74	C87	C82	-1.9(5)
C17	C16	C29	C28	177.5(3)	C75	C74	C87	C86	177.3(3)
C17	C18	C19	C20	0.0(6)	C75	C76	C77	C78	-2.3(5)
C17	C22	C23	C24	-1.1(6)	C75	C80	C81	C82	-1.1(5)

A	B	C	D	Angle/°	A	B	C	D	Angle/°
C18	C17	C22	C21	-3.7(5)	C76	C75	C80	C79	2.2(5)
C18	C17	C22	C23	172.6(3)	C76	C75	C80	C81	-176.6(3)
C18	C19	C20	C21	-1.9(6)	C76	C77	C78	C79	1.6(6)
C19	C20	C21	C22	0.8(7)	C77	C78	C79	C80	1.0(6)
C20	C21	C22	C17	2.0(6)	C78	C79	C80	C75	-2.9(5)
C20	C21	C22	C23	-174.2(4)	C78	C79	C80	C81	175.9(3)
C21	C22	C23	C24	175.0(4)	C79	C80	C81	C82	-179.8(3)
C22	C17	C18	C19	2.8(5)	C80	C75	C76	C77	0.4(5)
C22	C23	C24	C25	-174.9(4)	C80	C81	C82	C83	179.5(3)
C22	C23	C24	C29	4.0(5)	C80	C81	C82	C87	-1.9(5)
C23	C24	C25	C26	178.9(4)	C81	C82	C83	C84	178.2(4)
C23	C24	C29	C16	-2.0(5)	C81	C82	C87	C74	3.4(5)
C23	C24	C29	C28	177.8(3)	C81	C82	C87	C86	-175.8(3)
C24	C25	C26	C27	2.7(6)	C82	C83	C84	C85	-2.1(6)
C25	C24	C29	C16	176.9(3)	C83	C82	C87	C74	-177.9(3)
C25	C24	C29	C28	-3.3(5)	C83	C82	C87	C86	2.9(5)
C25	C26	C27	C28	-2.1(7)	C83	C84	C85	C86	2.3(7)
C26	C27	C28	C29	-1.3(6)	C84	C85	C86	C87	0.2(6)
C27	C28	C29	C16	-176.3(4)	C85	C86	C87	C74	178.1(3)
C27	C28	C29	C24	3.9(5)	C85	C86	C87	C82	-2.8(5)
C29	C16	C17	C18	-170.6(3)	C87	C74	C75	C76	178.1(3)
C29	C16	C17	C22	5.5(5)	C87	C74	C75	C80	-1.1(5)
C29	C24	C25	C26	0.0(6)	C87	C82	C83	C84	-0.5(6)
Br3	C36	C37	C38	176.8(3)	Br7	C94	C95	C96	-179.7(2)
Br4	C42	C43	C44	176.7(3)	Br8	C100	C101	C102	-178.8(3)
N3	C30	C31	N4	2.1(4)	N7	C88	C89	N8	0.7(3)
N3	C30	C31	C39	-173.5(3)	N7	C88	C89	C97	176.9(3)
N3	C30	C33	C34	35.7(5)	N7	C88	C91	C92	-75.2(4)
N3	C30	C33	C38	-139.7(3)	N7	C88	C91	C96	102.7(4)
N3	C32	C45	C46	101.6(4)	N7	C90	C103	C104	117.7(4)
N3	C32	C45	C58	-74.4(4)	N7	C90	C103	C116	-60.9(4)
N4	C31	C39	C40	-134.6(4)	N8	C89	C97	C98	-154.4(3)
N4	C31	C39	C44	44.7(5)	N8	C89	C97	C102	26.2(5)
N4	C32	C45	C46	-73.5(4)	N8	C90	C103	C104	-61.9(4)
N4	C32	C45	C58	110.5(4)	N8	C90	C103	C116	119.6(3)
C30	N3	C32	N4	1.0(4)	C88	N7	C90	N8	-1.0(3)
C30	N3	C32	C45	-174.7(3)	C88	N7	C90	C103	179.4(3)
C30	C31	C39	C40	40.2(5)	C88	C89	C97	C98	30.0(5)
C30	C31	C39	C44	-140.4(4)	C88	C89	C97	C102	-149.5(4)
C30	C33	C34	C35	-176.7(3)	C88	C91	C92	C93	178.7(3)
C30	C33	C38	C37	177.2(3)	C88	C91	C96	C95	-177.5(3)

A	B	C	D	Angle/°	A	B	C	D	Angle/°
C31	N4	C32	N3	0.3(4)	C89	N8	C90	N7	1.4(4)
C31	N4	C32	C45	175.9(3)	C89	N8	C90	C103	-178.9(3)
C31	C30	C33	C34	-150.0(4)	C89	C88	C91	C92	97.7(4)
C31	C30	C33	C38	34.5(5)	C89	C88	C91	C96	-84.4(4)
C31	C39	C40	C41	178.6(3)	C89	C97	C98	C99	-179.4(3)
C31	C39	C44	C43	-177.3(3)	C89	C97	C102	C101	-178.7(3)
C32	N3	C30	C31	-1.9(4)	C90	N7	C88	C89	0.2(3)
C32	N3	C30	C33	173.4(3)	C90	N7	C88	C91	174.3(3)
C32	N4	C31	C30	-1.5(4)	C90	N8	C89	C88	-1.3(3)
C32	N4	C31	C39	174.5(3)	C90	N8	C89	C97	-178.0(3)
C32	C45	C46	C47	1.1(5)	C90	C103	C104	C105	-6.0(5)
C32	C45	C46	C51	-176.3(3)	C90	C103	C104	C109	177.4(3)
C32	C45	C58	C53	177.4(3)	C90	C103	C116	C111	180.0(3)
C32	C45	C58	C57	-4.0(5)	C90	C103	C116	C115	0.5(5)
C33	C30	C31	N4	-172.6(3)	C91	C88	C89	N8	-172.8(3)
C33	C30	C31	C39	11.8(6)	C91	C88	C89	C97	3.4(6)
C33	C34	C35	C36	-0.6(5)	C91	C92	C93	C94	-1.4(5)
C34	C33	C38	C37	1.8(5)	C92	C91	C96	C95	0.5(5)
C34	C35	C36	Br3	-176.2(3)	C92	C93	C94	Br7	-179.2(3)
C34	C35	C36	C37	1.9(6)	C92	C93	C94	C95	1.0(5)
C35	C36	C37	C38	-1.4(6)	C93	C94	C95	C96	0.1(5)
C36	C37	C38	C33	-0.5(5)	C94	C95	C96	C91	-0.9(5)
C38	C33	C34	C35	-1.2(5)	C96	C91	C92	C93	0.6(5)
C39	C40	C41	C42	-1.0(6)	C97	C98	C99	C100	-1.6(6)
C40	C39	C44	C43	2.0(5)	C98	C97	C102	C101	1.8(5)
C40	C41	C42	Br4	-175.4(3)	C98	C99	C100	Br8	-179.4(3)
C40	C41	C42	C43	1.6(6)	C98	C99	C100	C101	1.4(6)
C41	C42	C43	C44	-0.4(5)	C99	C100	C101	C102	0.4(5)
C42	C43	C44	C39	-1.5(5)	C100	C101	C102	C97	-2.0(5)
C44	C39	C40	C41	-0.8(5)	C102	C97	C98	C99	0.0(5)
C45	C46	C47	C48	-179.0(3)	C103	C104	C105	C106	-177.4(3)
C45	C46	C51	C50	179.5(3)	C103	C104	C109	C108	-179.9(3)
C45	C46	C51	C52	-0.5(5)	C103	C104	C109	C110	2.7(5)
C46	C45	C58	C53	1.5(5)	C104	C103	C116	C111	1.4(5)
C46	C45	C58	C57	-179.9(3)	C104	C103	C116	C115	-178.1(3)
C46	C47	C48	C49	-0.2(5)	C104	C105	C106	C107	-1.7(5)
C46	C51	C52	C53	0.0(5)	C104	C109	C110	C111	1.5(5)
C47	C46	C51	C50	2.0(5)	C105	C104	C109	C108	3.4(5)
C47	C46	C51	C52	-178.0(3)	C105	C104	C109	C110	-174.1(3)
C47	C48	C49	C50	1.6(6)	C105	C106	C107	C108	1.7(5)
C48	C49	C50	C51	-1.2(6)	C106	C107	C108	C109	1.0(6)

A	B	C	D	Angle/˚	A	B	C	D	Angle/˚
C49	C50	C51	C46	-0.7(6)	C107	C108	C109	C104	-3.5(5)
C49	C50	C51	C52	179.4(4)	C107	C108	C109	C110	173.9(4)
C50	C51	C52	C53	180.0(4)	C108	C109	C110	C111	-175.9(3)
C51	C46	C47	C48	-1.6(5)	C109	C104	C105	C106	-0.9(5)
C51	C52	C53	C54	-177.7(4)	C109	C110	C111	C112	175.2(3)
C51	C52	C53	C58	1.2(6)	C109	C110	C111	C116	-4.2(5)
C52	C53	C54	C55	179.3(4)	C110	C111	C112	C113	-179.5(4)
C52	C53	C58	C45	-2.0(5)	C110	C111	C116	C103	2.7(5)
C52	C53	C58	C57	179.4(3)	C110	C111	C116	C115	-177.8(3)
C53	C54	C55	C56	1.8(6)	C111	C112	C113	C114	-1.8(6)
C54	C53	C58	C45	177.0(3)	C112	C111	C116	C103	-176.7(3)
C54	C53	C58	C57	-1.7(5)	C112	C111	C116	C115	2.8(5)
C54	C55	C56	C57	-2.8(6)	C112	C113	C114	C115	0.7(6)
C55	C56	C57	C58	1.5(6)	C113	C114	C115	C116	2.2(5)
C56	C57	C58	C45	-177.9(3)	C114	C115	C116	C103	175.6(3)
C56	C57	C58	C53	0.7(5)	C114	C115	C116	C111	-3.9(5)
C58	C45	C46	C47	177.1(3)	C116	C103	C104	C105	172.5(3)
C58	C45	C46	C51	-0.3(5)	C116	C103	C104	C109	-4.1(5)
C58	C53	C54	C55	0.4(6)	C116	C111	C112	C113	-0.1(5)

Table 6 Hydrogen Bonds

D	H	A	d(D-H)/Å	d(H-A)/Å	d(D-A)/Å	D-H-A/°
N2	H2	N3	0.91(5)	2.01(5)	2.898(4)	164(4)
N4	H4	N5	0.79(5)	2.13(5)	2.917(4)	173(5)
N6	H6A	N7	0.84(5)	2.09(5)	2.916(4)	171(5)
N8	H8	N1[1]	0.80(4)	2.20(4)	2.982(4)	166(4)

[1]1+X,-1+Y,+Z

*6.2 Crystallographic Data and Detailed Refinements for **5b***

Table S-7 Crystal data and structure refinement

Identification code	AM-TBA
Empirical formula	$C_{45}H_{28}N_2S_2$
Formula weight	660.81
Temperature/K	100(2)

Crystal system	triclinic
Space group	*P*-1
a/Å	11.57220(10)
b/Å	13.9231(2)
c/Å	24.5599(3)
α/°	91.4770(10)
β/°	94.0740(10)
γ/°	99.8870(10)
Volume/Å^3	3885.59(8)
Z	4
ρ_{calc}g/cm^3	1.130
μ/mm^{-1}	1.477
F(000)	1376.0
Crystal size/mm^3	0.149 × 0.094 × 0.055
Radiation	Cu *K*α (*λ* = 1.54184)
2*θ* range for data collection/°	6.448 to 158.748
Index ranges	$-14 \le h \le 14$, $-17 \le k \le 17$, $-30 \le l \le 31$
Reflections collected	96398
Independent reflections	16607 [R_{int} = 0.0426, R_{sigma} = 0.0276]
Data/restraints/parameters	16607/0/891
Goodness-of-fit on F^2	1.065
Final *R* indexes [*I*>=2σ (I)]	R_1 = 0.0432, wR_2 = 0.1043
Final *R* indexes [all data]	R_1 = 0.0481, wR_2 = 0.1070
Largest diff. peak/hole / e Å^{-3}	0.56/-0.49

Table S-8 Fractional Atomic Coordinates (×10^4) and Equivalent Isotropic Displacement Parameters (Å^2×10^3). U_{eq} is defined as 1/3 of the trace of the orthogonalized U_{IJ} tensor.

Atom	*x*	*y*	*z*	U(eq)
S1	11787.9(3)	3462.1(3)	4519.0(2)	25.99(9)
S2	3637.5(4)	-4196.4(3)	3683.8(2)	28.00(9)
N1	5922.5(12)	2461.3(10)	1893.6(5)	19.6(3)
N2	4295.2(12)	1365.1(10)	1785.4(5)	20.4(3)
C1	6024.8(13)	1800.8(11)	2296.9(6)	19.1(3)
C2	5005.0(14)	1113.1(11)	2219.5(6)	19.5(3)
C3	4879.6(14)	2178.3(11)	1601.6(6)	20.1(3)
C4	7008.3(14)	1972.7(11)	2718.9(6)	19.5(3)
C5	6857.7(14)	1671.8(12)	3250.8(7)	22.8(3)
C6	7752.1(14)	1913.7(12)	3663.6(7)	23.1(3)
C7	8825.0(14)	2481.9(11)	3561.6(7)	20.9(3)

Atom	*x*	*y*	*z*	U(eq)
C8	8992.7(14)	2747.4(12)	3023.7(7)	22.4(3)
C9	8102.9(14)	2497.3(11)	2608.9(7)	21.4(3)
C10	10916.2(14)	2930.6(12)	3951.9(7)	23.9(3)
C11	9740.3(14)	2844.2(11)	4006.7(7)	21.5(3)
C12	9508.2(14)	3213.3(11)	4537.3(7)	22.3(3)
C13	8436.5(15)	3290.7(12)	4755.8(7)	24.9(3)
C14	8438.4(16)	3698.4(13)	5273.8(7)	27.9(4)
C15	9493.6(17)	4054.0(13)	5587.0(7)	29.3(4)
C16	10557.5(16)	4004.7(12)	5381.8(7)	27.2(4)
C17	10557.7(14)	3580.2(12)	4860.4(7)	23.9(3)
C18	4641.4(14)	203.3(11)	2502.2(6)	20.3(3)
C19	3474.3(14)	-88.0(12)	2620.3(7)	24.4(3)
C20	3114.8(15)	-972.9(12)	2856.9(7)	25.7(3)
C21	3904.0(14)	-1612.0(11)	2965.5(7)	22.0(3)
C22	5076.0(14)	-1314.6(12)	2850.1(7)	21.9(3)
C23	5441.1(14)	-417.5(11)	2629.3(6)	20.8(3)
C24	4201.3(15)	-3002.9(12)	3551.7(7)	26.5(3)
C25	3533.7(14)	-2590.5(12)	3178.7(7)	23.2(3)
C26	2477.2(14)	-3265.5(11)	2985.9(7)	21.5(3)
C27	1575.5(14)	-3150.1(12)	2592.2(7)	23.3(3)
C28	648.4(15)	-3904.6(12)	2471.7(7)	25.9(3)
C29	594.9(15)	-4793.2(13)	2730.1(7)	27.7(4)
C30	1480.2(15)	-4939.7(12)	3108.9(7)	27.1(3)
C31	2419.3(15)	-4173.4(12)	3231.5(7)	23.3(3)
C32	4496.6(14)	2745.8(11)	1139.6(6)	21.1(3)
C33	5090.6(14)	2773.3(12)	657.9(7)	22.5(3)
C34	5996.6(15)	2217.0(13)	576.9(7)	25.6(3)
C35	6519.9(16)	2230.0(14)	98.2(8)	30.8(4)
C36	6174.4(17)	2805.5(15)	-337.7(8)	34.5(4)
C37	5330.1(17)	3353.9(14)	-276.2(7)	32.5(4)
C38	4762.2(15)	3365.7(13)	222.3(7)	26.1(3)
C39	3891.9(16)	3924.4(13)	292.2(7)	29.3(4)
C40	3296.0(15)	3900.4(13)	767.2(7)	26.4(3)
C41	2403.2(17)	4474.7(14)	841.3(8)	33.9(4)
C42	1829.2(18)	4440.2(15)	1302.3(9)	36.6(4)
C43	2107.3(17)	3826.3(14)	1726.8(8)	34.0(4)
C44	2951.4(16)	3268.2(13)	1677.8(7)	28.8(4)
C45	3587.4(14)	3285.5(12)	1198.4(7)	23.2(3)
S3	11962.6(4)	11359.7(3)	5426.6(2)	27.21(9)
S4	5123.1(4)	3507.2(3)	4879.3(2)	23.16(8)
N3	9129.9(12)	8435.0(10)	2167.5(5)	19.5(3)

Atom	*x*	*y*	*z*	U(eq)
N4	7957.3(11)	7005.9(9)	2044.0(5)	19.7(3)
C46	9003.8(13)	8068.4(11)	2683.1(6)	18.6(3)
C47	8264.9(13)	7178.9(11)	2598.6(6)	18.3(3)
C48	8490.1(13)	7777.5(11)	1800.0(6)	18.6(3)
C49	9641.7(13)	8580.6(11)	3173.0(6)	19.6(3)
C50	10113.6(14)	8064.4(11)	3589.0(6)	20.2(3)
C51	10722.8(14)	8548.4(12)	4051.5(6)	21.1(3)
C52	10893.0(14)	9568.0(11)	4112.0(6)	20.7(3)
C53	10420.1(14)	10081.3(12)	3693.5(7)	23.2(3)
C54	9803.1(14)	9597.6(12)	3232.7(7)	22.8(3)
C55	11148.9(15)	10892.9(12)	4834.0(7)	24.2(3)
C56	11494.2(14)	10094.7(11)	4611.6(6)	21.2(3)
C57	12488.0(14)	9831.4(12)	4930.1(6)	22.2(3)
C58	13142.5(15)	9094.7(12)	4838.0(7)	25.2(3)
C59	14080.4(15)	8998.2(13)	5203.5(8)	29.2(4)
C60	14390.4(15)	9631.3(14)	5664.5(7)	30.7(4)
C61	13776.2(16)	10368.5(14)	5762.4(7)	29.6(4)
C62	12831.3(15)	10469.8(12)	5393.3(7)	24.8(3)
C63	7751.5(13)	6471.2(11)	2987.7(6)	18.6(3)
C64	7597.1(14)	5469.3(11)	2860.4(6)	20.7(3)
C65	7016.4(14)	4796.3(11)	3198.3(7)	21.4(3)
C66	6563.2(13)	5095.9(11)	3674.0(6)	19.3(3)
C67	6762.0(14)	6096.3(11)	3812.8(6)	20.1(3)
C68	7345.5(13)	6769.4(11)	3474.8(6)	19.6(3)
C69	5996.0(14)	4447.9(12)	4574.8(7)	22.2(3)
C70	5890.1(13)	4384.7(11)	4021.2(6)	19.7(3)
C71	5036.3(13)	3539.9(11)	3816.3(6)	19.3(3)
C72	4606.6(14)	3243.2(12)	3278.9(7)	22.5(3)
C73	3783.0(15)	2400.7(12)	3183.0(7)	24.3(3)
C74	3363.8(14)	1837.9(12)	3615.9(7)	24.4(3)
C75	3733.4(14)	2130.5(12)	4149.8(7)	22.2(3)
C76	4561.6(14)	2981.2(11)	4243.5(6)	20.4(3)
C77	8349.4(14)	7933.4(11)	1205.7(6)	19.7(3)
C78	7474.5(14)	8453.5(11)	1012.9(6)	20.9(3)
C79	6737.1(15)	8848.8(12)	1368.0(7)	25.4(3)
C80	5922.6(16)	9374.9(14)	1169.6(8)	30.7(4)
C81	5785.4(16)	9532.5(14)	601.5(8)	32.5(4)
C82	6447.5(16)	9149.3(14)	248.9(7)	30.7(4)
C83	7312.3(15)	8592.3(13)	437.4(7)	25.6(3)
C84	7989.5(16)	8173.2(15)	80.5(7)	31.1(4)
C85	8850.7(15)	7652.0(14)	269.2(7)	28.8(4)

Atom	*x*	*y*	*z*	U(eq)
C86	9549.2(18)	7223.5(16)	-94.1(8)	37.8(4)
C87	10418.7(18)	6757.5(16)	97.4(9)	39.9(5)
C88	10646.0(17)	6678.3(14)	667.1(9)	34.3(4)
C89	9986.8(15)	7050.7(13)	1028.2(8)	27.6(4)
C90	9053.6(14)	7545.5(12)	846.2(7)	22.5(3)

Table S-9 Selected Bond Distances (Å)

Atom	Atom	Length/Å	Atom	Atom	Length/Å
S1	C10	1.7308(17)	S3	C55	1.7245(17)
S1	C17	1.7320(18)	S3	C62	1.7282(19)
S2	C24	1.7255(17)	S4	C69	1.7316(16)
S2	C31	1.7369(17)	S4	C76	1.7407(16)
N1	C1	1.3816(19)	N3	C46	1.3854(19)
N1	C3	1.351(2)	N3	C48	1.353(2)
N2	C2	1.389(2)	N4	C47	1.3874(19)
N2	C3	1.323(2)	N4	C48	1.323(2)
C1	C2	1.384(2)	C46	C47	1.380(2)
C1	C4	1.467(2)	C46	C49	1.466(2)
C2	C18	1.472(2)	C47	C63	1.472(2)
C3	C32	1.484(2)	C48	C77	1.483(2)
C4	C5	1.396(2)	C49	C50	1.397(2)
C4	C9	1.397(2)	C49	C54	1.399(2)
C5	C6	1.388(2)	C50	C51	1.387(2)
C6	C7	1.395(2)	C51	C52	1.402(2)
C7	C8	1.399(2)	C52	C53	1.400(2)
C7	C11	1.482(2)	C52	C56	1.478(2)
C8	C9	1.389(2)	C53	C54	1.388(2)
C10	C11	1.362(2)	C55	C56	1.358(2)
C11	C12	1.448(2)	C56	C57	1.449(2)
C12	C13	1.405(2)	C57	C58	1.399(2)
C12	C17	1.412(2)	C57	C62	1.416(2)
C13	C14	1.379(2)	C58	C59	1.385(2)
C14	C15	1.403(3)	C59	C60	1.404(3)
C15	C16	1.375(3)	C60	C61	1.371(3)
C16	C17	1.397(2)	C61	C62	1.399(2)
C18	C19	1.395(2)	C63	C64	1.400(2)
C18	C23	1.396(2)	C63	C68	1.395(2)
C19	C20	1.387(2)	C64	C65	1.386(2)
C20	C21	1.398(2)	C65	C66	1.397(2)

Atom	Atom	Length/Å	Atom	Atom	Length/Å
C21	C22	1.400(2)	C66	C67	1.401(2)
C21	C25	1.477(2)	C66	C70	1.479(2)
C22	C23	1.385(2)	C67	C68	1.388(2)
C24	C25	1.358(2)	C69	C70	1.356(2)
C25	C26	1.450(2)	C70	C71	1.453(2)
C26	C27	1.404(2)	C71	C72	1.403(2)
C26	C31	1.408(2)	C71	C76	1.409(2)
C27	C28	1.376(2)	C72	C73	1.382(2)
C28	C29	1.399(2)	C73	C74	1.402(2)
C29	C30	1.379(2)	C74	C75	1.379(2)
C30	C31	1.396(2)	C75	C76	1.393(2)
C32	C33	1.408(2)	C77	C78	1.405(2)
C32	C45	1.407(2)	C77	C90	1.401(2)
C33	C34	1.428(2)	C78	C79	1.426(2)
C33	C38	1.437(2)	C78	C83	1.435(2)
C34	C35	1.359(3)	C79	C80	1.361(2)
C35	C36	1.429(3)	C80	C81	1.420(3)
C36	C37	1.353(3)	C81	C82	1.354(3)
C37	C38	1.431(3)	C82	C83	1.427(2)
C38	C39	1.391(3)	C83	C84	1.397(3)
C39	C40	1.395(3)	C84	C85	1.391(3)
C40	C41	1.429(3)	C85	C86	1.430(3)
C40	C45	1.436(2)	C85	C90	1.436(2)
C41	C42	1.351(3)	C86	C87	1.352(3)
C42	C43	1.419(3)	C87	C88	1.416(3)
C43	C44	1.358(3)	C88	C89	1.358(3)
C44	C45	1.431(2)	C89	C90	1.430(2)

Table S-10 Selected Bond Angles

Atom	Atom	Atom	Angle/°	Atom	Atom	Atom	Angle/°
C10	S1	C17	91.24(8)	C55	S3	C62	91.15(8)
C24	S2	C31	90.78(8)	C69	S4	C76	91.12(8)
C3	N1	C1	108.42(13)	C48	N3	C46	108.03(13)
C3	N2	C2	105.87(13)	C48	N4	C47	106.05(13)
N1	C1	C2	104.85(13)	N3	C46	C49	121.88(13)
N1	C1	C4	121.06(14)	C47	C46	N3	105.17(13)
C2	C1	C4	133.76(14)	C47	C46	C49	132.84(14)
N2	C2	C18	120.09(14)	N4	C47	C63	119.32(13)
C1	C2	N2	109.67(13)	C46	C47	N4	109.58(13)

Atom	Atom	Atom	Angle/°	Atom	Atom	Atom	Angle/°
C1	C2	C18	130.18(14)	C46	C47	C63	131.02(14)
N1	C3	C32	121.72(14)	N3	C48	C77	124.10(14)
N2	C3	N1	111.19(13)	N4	C48	N3	111.18(13)
N2	C3	C32	127.09(14)	N4	C48	C77	124.63(14)
C5	C4	C1	120.99(14)	C50	C49	C46	120.74(14)
C5	C4	C9	117.98(14)	C50	C49	C54	118.40(14)
C9	C4	C1	120.90(14)	C54	C49	C46	120.85(14)
C6	C5	C4	121.21(15)	C51	C50	C49	120.82(14)
C5	C6	C7	120.94(15)	C50	C51	C52	121.01(14)
C6	C7	C8	117.74(15)	C51	C52	C56	121.86(14)
C6	C7	C11	121.90(15)	C53	C52	C51	117.91(14)
C8	C7	C11	120.29(14)	C53	C52	C56	120.14(14)
C9	C8	C7	121.31(15)	C54	C53	C52	121.12(15)
C8	C9	C4	120.68(15)	C53	C54	C49	120.73(15)
C11	C10	S1	113.86(13)	C56	C55	S3	114.19(13)
C10	C11	C7	123.65(15)	C55	C56	C52	122.78(15)
C10	C11	C12	111.55(15)	C55	C56	C57	111.58(15)
C12	C11	C7	124.67(14)	C57	C56	C52	125.63(15)
C13	C12	C11	130.31(15)	C58	C57	C56	130.46(15)
C13	C12	C17	117.75(16)	C58	C57	C62	118.12(15)
C17	C12	C11	111.88(15)	C62	C57	C56	111.38(15)
C14	C13	C12	119.78(15)	C59	C58	C57	119.62(17)
C13	C14	C15	121.30(17)	C58	C59	C60	121.10(17)
C16	C15	C14	120.41(17)	C61	C60	C59	120.70(16)
C15	C16	C17	118.40(16)	C60	C61	C62	118.33(17)
C12	C17	S1	111.46(13)	C57	C62	S3	111.70(13)
C16	C17	S1	126.16(13)	C61	C62	S3	126.18(14)
C16	C17	C12	122.35(16)	C61	C62	C57	122.11(17)
C19	C18	C2	120.83(14)	C64	C63	C47	120.15(14)
C19	C18	C23	118.33(14)	C68	C63	C47	121.71(14)
C23	C18	C2	120.74(14)	C68	C63	C64	118.04(14)
C20	C19	C18	120.95(15)	C65	C64	C63	120.87(15)
C19	C20	C21	120.81(15)	C64	C65	C66	121.13(15)
C20	C21	C22	118.06(15)	C65	C66	C67	117.88(14)
C20	C21	C25	122.33(15)	C65	C66	C70	121.37(14)
C22	C21	C25	119.58(14)	C67	C66	C70	120.75(14)
C23	C22	C21	121.00(15)	C68	C67	C66	120.88(14)
C22	C23	C18	120.78(15)	C67	C68	C63	121.07(14)
C25	C24	S2	114.50(13)	C70	C69	S4	114.02(12)
C24	C25	C21	123.54(15)	C69	C70	C66	123.61(14)
C24	C25	C26	111.26(15)	C69	C70	C71	111.65(14)

Atom	Atom	Atom	Angle/°	Atom	Atom	Atom	Angle/°
C26	C25	C21	124.99(15)	C71	C70	C66	124.74(14)
C27	C26	C25	129.95(15)	C72	C71	C70	130.16(14)
C27	C26	C31	118.22(15)	C72	C71	C76	117.92(14)
C31	C26	C25	111.77(14)	C76	C71	C70	111.87(14)
C28	C27	C26	119.71(15)	C73	C72	C71	119.73(15)
C27	C28	C29	121.09(16)	C72	C73	C74	120.95(15)
C30	C29	C28	120.71(16)	C75	C74	C73	120.71(15)
C29	C30	C31	118.16(15)	C74	C75	C76	118.00(15)
C26	C31	S2	111.68(12)	C71	C76	S4	111.32(12)
C30	C31	S2	126.26(13)	C75	C76	S4	126.09(12)
C30	C31	C26	122.07(15)	C75	C76	C71	122.58(15)
C33	C32	C3	119.24(14)	C78	C77	C48	118.38(14)
C45	C32	C3	119.70(15)	C90	C77	C48	120.42(14)
C45	C32	C33	120.98(15)	C90	C77	C78	121.18(14)
C32	C33	C34	122.67(15)	C77	C78	C79	122.47(15)
C32	C33	C38	119.22(15)	C77	C78	C83	118.97(15)
C34	C33	C38	118.10(15)	C79	C78	C83	118.56(15)
C35	C34	C33	121.16(16)	C80	C79	C78	120.94(16)
C34	C35	C36	120.53(17)	C79	C80	C81	120.34(17)
C37	C36	C35	120.29(17)	C82	C81	C80	120.59(16)
C36	C37	C38	120.96(17)	C81	C82	C83	121.13(16)
C37	C38	C33	118.93(16)	C82	C83	C78	118.40(16)
C39	C38	C33	119.36(16)	C84	C83	C78	119.41(16)
C39	C38	C37	121.71(16)	C84	C83	C82	122.18(16)
C38	C39	C40	121.84(16)	C85	C84	C83	121.76(16)
C39	C40	C41	122.04(16)	C84	C85	C86	122.01(17)
C39	C40	C45	119.36(16)	C84	C85	C90	119.17(16)
C41	C40	C45	118.60(16)	C86	C85	C90	118.81(17)
C42	C41	C40	121.39(17)	C87	C86	C85	121.24(19)
C41	C42	C43	120.14(17)	C86	C87	C88	120.06(18)
C44	C43	C42	120.84(18)	C89	C88	C87	120.92(18)
C43	C44	C45	120.98(17)	C88	C89	C90	121.16(17)
C32	C45	C40	119.17(15)	C77	C90	C85	119.42(15)
C32	C45	C44	122.76(15)	C77	C90	C89	122.84(15)
C44	C45	C40	118.05(15)	C89	C90	C85	117.74(16)

Table S-11 Selected Torsion Angles

A	B	C	D	Angle/°	A	B	C	D	Angle/°
S1	C10	C11	C7	175.20(12)	S3	C55	C56	C52	178.32(12)
S1	C10	C11	C12	-0.74(17)	S3	C55	C56	C57	-0.56(18)
S2	C24	C25	C21	-173.88(13)	S4	C69	C70	C66	-178.96(12)
S2	C24	C25	C26	1.0(2)	S4	C69	C70	C71	1.26(18)
N1	C1	C2	N2	-1.09(17)	N3	C46	C47	N4	0.48(17)
N1	C1	C2	C18	176.00(16)	N3	C46	C47	C63	-176.08(15)
N1	C1	C4	C5	145.76(16)	N3	C46	C49	C50	-140.28(16)
N1	C1	C4	C9	-30.1(2)	N3	C46	C49	C54	39.1(2)
N1	C3	C32	C33	68.9(2)	N3	C48	C77	C78	-84.04(19)
N1	C3	C32	C45	-107.98(18)	N3	C48	C77	C90	97.71(19)
N2	C2	C18	C19	-41.4(2)	N4	C47	C63	C64	39.2(2)
N2	C2	C18	C23	134.77(16)	N4	C47	C63	C68	-137.10(15)
N2	C3	C32	C33	-111.23(19)	N4	C48	C77	C78	92.00(19)
N2	C3	C32	C45	71.9(2)	N4	C48	C77	C90	-86.3(2)
C1	N1	C3	N2	-0.66(18)	C46	N3	C48	N4	-0.09(18)
C1	N1	C3	C32	179.23(14)	C46	N3	C48	C77	176.42(14)
C1	C2	C18	C19	141.80(18)	C46	C47	C63	C64	-144.49(17)
C1	C2	C18	C23	-42.1(3)	C46	C47	C63	C68	39.2(2)
C1	C4	C5	C6	-173.78(15)	C46	C49	C50	C51	179.66(14)
C1	C4	C9	C8	173.12(15)	C46	C49	C54	C53	-179.18(15)
C2	N2	C3	N1	-0.03(18)	C47	N4	C48	N3	0.38(17)
C2	N2	C3	C32	-179.91(16)	C47	N4	C48	C77	-176.10(14)
C2	C1	C4	C5	-26.5(3)	C47	C46	C49	C50	35.4(3)
C2	C1	C4	C9	157.62(17)	C47	C46	C49	C54	-145.28(18)
C2	C18	C19	C20	176.09(16)	C47	C63	C64	C65	-173.96(14)
C2	C18	C23	C22	-174.09(15)	C47	C63	C68	C67	173.77(14)
C3	N1	C1	C2	1.05(17)	C48	N3	C46	C47	-0.25(17)
C3	N1	C1	C4	-173.18(14)	C48	N3	C46	C49	176.46(14)
C3	N2	C2	C1	0.72(18)	C48	N4	C47	C46	-0.53(17)
C3	N2	C2	C18	-176.72(14)	C48	N4	C47	C63	176.49(14)
C3	C32	C33	C34	4.8(2)	C48	C77	C78	C79	1.2(2)
C3	C32	C33	C38	-176.60(14)	C48	C77	C78	C83	-178.72(14)
C3	C32	C45	C40	174.48(15)	C48	C77	C90	C85	176.02(15)
C3	C32	C45	C44	-4.0(2)	C48	C77	C90	C89	-4.7(2)
C4	C1	C2	N2	172.06(16)	C49	C46	C47	N4	-175.71(16)
C4	C1	C2	C18	-10.8(3)	C49	C46	C47	C63	7.7(3)
C4	C5	C6	C7	1.2(3)	C49	C50	C51	C52	-0.7(2)
C5	C4	C9	C8	-2.9(2)	C50	C49	C54	C53	0.2(2)
C5	C6	C7	C8	-3.8(2)	C50	C51	C52	C53	0.6(2)

A	B	C	D	Angle/°	A	B	C	D	Angle/°
C5	C6	C7	C11	172.86(15)	C50	C51	C52	C56	177.30(15)
C6	C7	C8	C9	3.2(2)	C51	C52	C53	C54	-0.1(2)
C6	C7	C11	C10	144.33(17)	C51	C52	C56	C55	-141.76(17)
C6	C7	C11	C12	-40.3(2)	C51	C52	C56	C57	37.0(2)
C7	C8	C9	C4	0.2(2)	C52	C53	C54	C49	-0.3(3)
C7	C11	C12	C13	2.0(3)	C52	C56	C57	C58	4.1(3)
C7	C11	C12	C17	-175.14(14)	C52	C56	C57	C62	-178.23(14)
C8	C7	C11	C10	-39.1(2)	C53	C52	C56	C55	34.9(2)
C8	C7	C11	C12	136.34(16)	C53	C52	C56	C57	-146.38(16)
C9	C4	C5	C6	2.2(2)	C54	C49	C50	C51	0.3(2)
C10	S1	C17	C12	0.03(12)	C55	S3	C62	C57	0.08(13)
C10	S1	C17	C16	-178.17(15)	C55	S3	C62	C61	179.22(16)
C10	C11	C12	C13	177.85(16)	C55	C56	C57	C58	-177.07(16)
C10	C11	C12	C17	0.76(19)	C55	C56	C57	C62	0.61(19)
C11	C7	C8	C9	-173.57(15)	C56	C52	C53	C54	-176.88(15)
C11	C12	C13	C14	-178.15(16)	C56	C57	C58	C59	178.89(16)
C11	C12	C17	S1	-0.44(17)	C56	C57	C62	S3	-0.40(17)
C11	C12	C17	C16	177.83(15)	C56	C57	C62	C61	-179.58(15)
C12	C13	C14	C15	1.0(3)	C57	C58	C59	C60	-0.3(3)
C13	C12	C17	S1	-177.94(12)	C58	C57	C62	S3	177.60(12)
C13	C12	C17	C16	0.3(2)	C58	C57	C62	C61	-1.6(2)
C13	C14	C15	C16	0.1(3)	C58	C59	C60	C61	-0.5(3)
C14	C15	C16	C17	-1.0(3)	C59	C60	C61	C62	0.3(3)
C15	C16	C17	S1	178.76(13)	C60	C61	C62	S3	-178.31(13)
C15	C16	C17	C12	0.7(2)	C60	C61	C62	C57	0.7(2)
C17	S1	C10	C11	0.42(13)	C62	S3	C55	C56	0.28(14)
C17	C12	C13	C14	-1.2(2)	C62	C57	C58	C59	1.3(2)
C18	C19	C20	C21	-2.3(3)	C63	C64	C65	C66	0.3(2)
C19	C18	C23	C22	2.1(2)	C64	C63	C68	C67	-2.6(2)
C19	C20	C21	C22	2.7(3)	C64	C65	C66	C67	-2.9(2)
C19	C20	C21	C25	-175.34(16)	C64	C65	C66	C70	176.82(15)
C20	C21	C22	C23	-0.7(2)	C65	C66	C67	C68	2.8(2)
C20	C21	C25	C24	-142.37(19)	C65	C66	C70	C69	140.41(17)
C20	C21	C25	C26	43.5(3)	C65	C66	C70	C71	-39.9(2)
C21	C22	C23	C18	-1.7(2)	C66	C67	C68	C63	0.0(2)
C21	C25	C26	C27	-3.0(3)	C66	C70	C71	C72	-4.6(3)
C21	C25	C26	C31	174.41(16)	C66	C70	C71	C76	178.28(14)
C22	C21	C25	C24	39.7(3)	C67	C66	C70	C69	-39.8(2)
C22	C21	C25	C26	-134.51(17)	C67	C66	C70	C71	139.91(16)
C23	C18	C19	C20	-0.1(3)	C68	C63	C64	C65	2.5(2)
C24	S2	C31	C26	0.79(14)	C69	S4	C76	C71	-0.91(12)

A	B	C	D	Angle/°	A	B	C	D	Angle/°
C24	S2	C31	C30	-179.64(17)	C69	S4	C76	C75	-179.74(15)
C24	C25	C26	C27	-177.79(17)	C69	C70	C71	C72	175.18(16)
C24	C25	C26	C31	-0.4(2)	C69	C70	C71	C76	-1.95(19)
C25	C21	C22	C23	177.40(15)	C70	C66	C67	C68	-176.96(14)
C25	C26	C27	C28	179.55(17)	C70	C71	C72	C73	-179.89(16)
C25	C26	C31	S2	-0.39(18)	C70	C71	C76	S4	1.77(17)
C25	C26	C31	C30	-179.98(16)	C70	C71	C76	C75	-179.36(14)
C26	C27	C28	C29	-0.8(3)	C71	C72	C73	C74	0.3(2)
C27	C26	C31	S2	177.36(12)	C72	C71	C76	S4	-175.75(12)
C27	C26	C31	C30	-2.2(2)	C72	C71	C76	C75	3.1(2)
C27	C28	C29	C30	-0.8(3)	C72	C73	C74	C75	2.3(3)
C28	C29	C30	C31	0.9(3)	C73	C74	C75	C76	-2.1(2)
C29	C30	C31	S2	-178.86(14)	C74	C75	C76	S4	178.08(13)
C29	C30	C31	C26	0.7(3)	C74	C75	C76	C71	-0.6(2)
C31	S2	C24	C25	-1.05(15)	C76	S4	C69	C70	-0.22(13)
C31	C26	C27	C28	2.3(2)	C76	C71	C72	C73	-2.9(2)
C32	C33	C34	C35	177.32(16)	C77	C78	C79	C80	177.94(16)
C32	C33	C38	C37	-177.02(16)	C77	C78	C83	C82	-177.95(15)
C32	C33	C38	C39	2.1(2)	C77	C78	C83	C84	2.8(2)
C33	C32	C45	C40	-2.3(2)	C78	C77	C90	C85	-2.2(2)
C33	C32	C45	C44	179.17(16)	C78	C77	C90	C89	177.10(15)
C33	C34	C35	C36	0.1(3)	C78	C79	C80	C81	0.6(3)
C33	C38	C39	C40	-2.3(3)	C78	C83	C84	C85	-2.4(3)
C34	C33	C38	C37	1.6(2)	C79	C78	C83	C82	2.2(2)
C34	C33	C38	C39	-179.25(16)	C79	C78	C83	C84	-177.13(16)
C34	C35	C36	C37	0.8(3)	C79	C80	C81	C82	1.1(3)
C35	C36	C37	C38	-0.4(3)	C80	C81	C82	C83	-1.1(3)
C36	C37	C38	C33	-0.8(3)	C81	C82	C83	C78	-0.6(3)
C36	C37	C38	C39	-179.92(18)	C81	C82	C83	C84	178.68(18)
C37	C38	C39	C40	176.76(17)	C82	C83	C84	C85	178.36(18)
C38	C33	C34	C35	-1.3(2)	C83	C78	C79	C80	-2.2(3)
C38	C39	C40	C41	179.85(18)	C83	C84	C85	C86	-179.82(18)
C38	C39	C40	C45	0.2(3)	C83	C84	C85	C90	-0.3(3)
C39	C40	C41	C42	179.65(19)	C84	C85	C86	C87	176.9(2)
C39	C40	C45	C32	2.1(2)	C84	C85	C90	C77	2.6(3)
C39	C40	C45	C44	-179.30(16)	C84	C85	C90	C89	-176.73(16)
C40	C41	C42	C43	0.1(3)	C85	C86	C87	C88	0.6(3)
C41	C40	C45	C32	-177.52(16)	C86	C85	C90	C77	-177.88(16)
C41	C40	C45	C44	1.0(2)	C86	C85	C90	C89	2.8(2)
C41	C42	C43	C44	0.1(3)	C86	C87	C88	C89	1.2(3)
C42	C43	C44	C45	0.2(3)	C87	C88	C89	C90	-0.8(3)

A	B	C	D	Angle/˚	A	B	C	D	Angle/˚
C43	C44	C45	C32	177.66(17)	C88	C89	C90	C77	179.55(16)
C43	C44	C45	C40	-0.8(3)	C88	C89	C90	C85	-1.2(3)
C45	C32	C33	C34	-178.35(15)	C90	C77	C78	C79	179.40(15)
C45	C32	C33	C38	0.2(2)	C90	C77	C78	C83	-0.5(2)
C45	C40	C41	C42	-0.7(3)	C90	C85	C86	C87	-2.6(3)

6.3 Crystallographic Data and Detailed Refinements for **5b** solvate

Table S-12 Crystal data and structure refinement

Identification code	TBA
Empirical formula	$C_{47}H_{36}N_2O_2S_2$
Formula weight	724.90
Temperature/K	123(30)
Crystal system	monoclinic
Space group	*I*2/*a*
a/Å	15.62220(10)
b/Å	11.89850(10)
c/Å	19.71010(10)
α/°	90
β/°	105.1980(10)
γ/°	90
Volume/Å^3	3535.59(4)
Z	4
ρ_{calc}g/cm^3	1.362
μ/mm^{-1}	1.713
F(000)	1520.0
Crystal size/mm^3	0.168 × 0.087 × 0.081
Radiation	Cu *K*α (λ = 1.54184)
2θ range for data collection/°	8.766 to 158.688
Index ranges	$-18 \le h \le 19$, $-15 \le k \le 15$, $-25 \le l \le 24$
Reflections collected	43504
Independent reflections	3815 [R_{int} = 0.0344, R_{sigma} = 0.0151]
Data/restraints/parameters	3815/2/250
Goodness-of-fit on F^2	1.040
Final *R* indexes [*I*>=2σ (I)]	R_1 = 0.0386, wR_2 = 0.0956
Final *R* indexes [all data]	R_1 = 0.0393, wR_2 = 0.0960
Largest diff. peak/hole / e Å^{-3}	0.55/-0.30

Table S-13 Fractional Atomic Coordinates (×10^4) and Equivalent Isotropic Displacement Parameters ($Å^2×10^3$). U_{eq} is defined as 1/3 of the trace of the orthogonalized U_{IJ} tensor.

Atom	*x*	*y*	*z*	U(eq)
S1	5121.6(2)	8381.1(3)	3556.3(2)	23.84(11)
N1	3021.1(8)	1659.7(9)	4714.3(6)	16.6(2)
C1	5606.0(9)	7277.7(12)	3214.2(7)	20.5(3)
C2	6234.7(10)	7379.2(13)	2826.5(8)	24.4(3)
C3	6561.5(10)	6411.9(14)	2602.0(8)	26.1(3)
C4	6278.5(10)	5357.0(13)	2769.8(8)	24.4(3)
C5	5661.9(9)	5254.5(12)	3155.2(7)	21.5(3)
C6	5299.7(9)	6224.3(12)	3380.4(7)	18.9(3)
C7	4658.4(9)	6345.9(12)	3792.4(7)	18.6(3)
C8	4516.9(10)	7450.3(12)	3913.0(8)	21.5(3)
C9	4202.9(9)	5420.8(11)	4058.3(7)	17.6(3)
C10	3970.0(9)	4417.6(12)	3685.1(7)	19.0(3)
C11	3526.9(9)	3565.5(11)	3936.1(7)	18.2(3)
C12	3295.9(8)	3683.6(11)	4569.9(7)	15.7(3)
C13	3536.8(9)	4679.7(11)	4949.2(7)	17.0(3)
C14	3984.6(9)	5525.4(11)	4699.1(7)	17.9(3)
C15	2828.4(9)	2769.3(11)	4823.2(7)	15.9(3)
C16	2500	1023.9(16)	5000	16.8(4)
C17	2500	-216.4(15)	5000	15.3(3)
C18	2333.6(8)	-806.9(11)	4356.4(7)	16.0(3)
C19	2135.0(9)	-256.6(12)	3686.1(7)	20.4(3)
C20	1916.5(10)	-863.1(14)	3076.4(8)	25.3(3)
C21	1880.7(10)	-2054.9(14)	3085.5(8)	25.9(3)
C22	2088.4(10)	-2612.6(12)	3708.9(8)	22.6(3)
C23	2316.6(8)	-2010.7(11)	4360.5(7)	17.2(3)
C24	2500	-2582.7(16)	5000	18.7(4)
O1	4495.2(9)	606.4(13)	4450.5(9)	48.1(4)
C25	5064.2(11)	1276.0(15)	4173.1(11)	35.7(4)

Table S-14 Selected Bond Distances (Å)

Atom	Atom	Length/Å	Atom	Atom	Length/Å
S1	C1	1.7374(15)	C11	C12	1.3954(19)
S1	C8	1.7208(14)	C12	C13	1.3991(19)
N1	C15	1.3832(17)	C12	C15	1.4688(17)
N1	C16	1.3388(16)	C13	C14	1.3878(18)
C1	C2	1.399(2)	C15	C15[1]	1.383(3)
C1	C6	1.4105(19)	C16	C17	1.476(3)
C2	C3	1.378(2)	C17	C18[1]	1.4134(16)
C3	C4	1.399(2)	C17	C18	1.4134(16)
C4	C5	1.380(2)	C18	C19	1.4338(19)
C5	C6	1.407(2)	C18	C23	1.4326(18)
C6	C7	1.4529(19)	C19	C20	1.366(2)
C7	C8	1.364(2)	C20	C21	1.419(2)
C7	C9	1.4792(18)	C21	C22	1.359(2)
C9	C10	1.3994(19)	C22	C23	1.4317(19)
C9	C14	1.3974(19)	C23	C24	1.3945(16)
C10	C11	1.3897(19)	O1	C25	1.407(2)

[1]1/2-X,+Y,1-Z

Table S-15 Selected Bond Angles

Atom	Atom	Atom	Angle/°	Atom	Atom	Atom	Angle/°
C8	S1	C1	90.79(7)	C13	C12	C15	122.27(12)
C16	N1	C15	107.04(12)	C14	C13	C12	120.96(12)
C2	C1	S1	125.91(11)	C13	C14	C9	121.44(12)
C2	C1	C6	122.21(14)	N1	C15	C12	120.43(11)
C6	C1	S1	111.87(11)	C15[1]	C15	N1	107.37(7)
C3	C2	C1	118.40(14)	C15[1]	C15	C12	132.20(7)
C2	C3	C4	120.44(14)	N1	C16	N1[1]	111.19(17)
C5	C4	C3	121.28(15)	N1[1]	C16	C17	124.41(8)
C4	C5	C6	119.84(13)	N1	C16	C17	124.41(8)
C1	C6	C7	111.53(13)	C18	C17	C16	119.81(8)
C5	C6	C1	117.81(13)	C18[1]	C17	C16	119.81(8)
C5	C6	C7	130.63(13)	C18	C17	C18[1]	120.38(17)
C6	C7	C9	126.17(12)	C17	C18	C19	122.99(13)
C8	C7	C6	111.13(12)	C17	C18	C23	119.42(12)
C8	C7	C9	122.70(13)	C23	C18	C19	117.52(12)
C7	C8	S1	114.68(11)	C20	C19	C18	120.87(13)

Atom	Atom	Atom	Angle/°	Atom	Atom	Atom	Angle/°
C10	C9	C7	122.10(12)	C19	C20	C21	121.21(14)
C14	C9	C7	120.53(12)	C22	C21	C20	119.90(14)
C14	C9	C10	117.37(12)	C21	C22	C23	120.74(14)
C11	C10	C9	121.35(12)	C22	C23	C18	119.72(13)
C10	C11	C12	121.00(12)	C24	C23	C18	119.56(13)
C11	C12	C13	117.86(12)	C24	C23	C22	120.70(13)
C11	C12	C15	119.87(12)	C23[1]	C24	C23	121.58(18)

[1]1/2-X,+Y,1-Z

Table S-16 Selected Torsion Angles

A	B	C	D	Angle/°	A	B	C	D	Angle/°
S1	C1	C2	C3	179.09(12)	C10	C11	C12	C13	-1.0(2)
S1	C1	C6	C5	-177.89(11)	C10	C11	C12	C15	-179.94(12)
S1	C1	C6	C7	0.13(15)	C11	C12	C13	C14	0.52(19)
N1	C16	C17	C18[1]	122.87(8)	C11	C12	C15	N1	35.97(19)
N1[1]	C16	C17	C18[1]	-57.13(8)	C11	C12	C15	C15[1]	-143.54(19)
N1	C16	C17	C18	-57.13(8)	C12	C13	C14	C9	0.7(2)
N1[1]	C16	C17	C18	122.87(8)	C13	C12	C15	N1	-142.96(13)
C1	S1	C8	C7	0.19(12)	C13	C12	C15	C15[1]	37.5(3)
C1	C2	C3	C4	-1.0(2)	C14	C9	C10	C11	1.0(2)
C1	C6	C7	C8	0.01(17)	C15	N1	C16	N1[1]	0.20(7)
C1	C6	C7	C9	-179.93(13)	C15	N1	C16	C17	-179.80(7)
C2	C1	C6	C5	1.3(2)	C15	C12	C13	C14	179.47(12)
C2	C1	C6	C7	179.36(13)	C16	N1	C15	C12	179.86(10)
C2	C3	C4	C5	0.7(2)	C16	N1	C15	C15[1]	-0.51(17)
C3	C4	C5	C6	0.7(2)	C16	C17	C18	C19	-1.89(14)
C4	C5	C6	C1	-1.6(2)	C16	C17	C18	C23	-178.70(8)
C4	C5	C6	C7	-179.20(14)	C17	C18	C19	C20	-175.28(12)
C5	C6	C7	C8	177.70(14)	C17	C18	C23	C22	175.71(10)
C5	C6	C7	C9	-2.2(2)	C17	C18	C23	C24	-2.62(16)
C6	C1	C2	C3	0.0(2)	C18[1]	C17	C18	C19	178.11(14)
C6	C7	C8	S1	-0.15(16)	C18[1]	C17	C18	C23	1.30(8)
C6	C7	C9	C10	-33.5(2)	C18	C19	C20	C21	-0.1(2)
C6	C7	C9	C14	146.79(14)	C18	C23	C24	C23[1]	1.31(8)
C7	C9	C10	C11	-178.74(13)	C19	C18	C23	C22	-1.27(18)
C7	C9	C14	C13	178.30(12)	C19	C18	C23	C24	-179.60(10)
C8	S1	C1	C2	-179.37(14)	C19	C20	C21	C22	-1.7(2)
C8	S1	C1	C6	-0.18(11)	C20	C21	C22	C23	2.0(2)

A	B	C	D	Angle/˚	A	B	C	D	Angle/˚
C8	C7	C9	C10	146.56(14)	C21	C22	C23	C18	-0.5(2)
C8	C7	C9	C14	-33.1(2)	C21	C22	C23	C24	177.79(12)
C9	C7	C8	S1	179.79(10)	C22	C23	C24	C23[1]	-177.00(14)
C9	C10	C11	C12	0.2(2)	C23	C18	C19	C20	1.6(2)
C10	C9	C14	C13	-1.4(2)					

[1]1/2-X,+Y,1-Z

Table S-17 Hydrogen Bonds

D	H	A	d(D-H)/Å	d(H-A)/Å	d(D-A)/Å	D-H-A/°
N1	H1	O1	0.77(4)	2.04(4)	2.7879(18)	165(3)
O1	H1A	O1[1]	0.84	2.17	2.733(3)	124.3
O1	H1B	N1	0.838(19)	1.96(2)	2.7879(18)	172(5)

[1]1-X,-Y,1-Z

6.4 Crystallographic Data and Detailed Refinements for **6**

Table S-18 Crystal data and structure refinement

Identification code	AM-BAM
Empirical formula	$C_{30}H_{20}Br_2N_2$
Formula weight	568.30
Temperature/K	293(2)
Crystal system	orthorhombic
Space group	$Pna2_1$
a/Å	15.5732(3)
b/Å	11.1891(3)
c/Å	28.3670(6)
α/°	90
β/°	90
γ/°	90
Volume/Å^3	4942.95(19)
Z	8
ρ_{calc}g/cm^3	1.527
μ/mm^{-1}	4.314
F(000)	2272.0

Crystal size/mm^3	0.183 × 0.122 × 0.047
Radiation	Cu $K\alpha$ (λ = 1.54184)
2θ range for data collection/°	6.232 to 159.148
Index ranges	$-19 \le h \le 14$, $-14 \le k \le 14$, $-36 \le l \le 36$
Reflections collected	57548
Independent reflections	10338 [R_{int} = 0.0328, R_{sigma} = 0.0202]
Data/restraints/parameters	10338/1/616
Goodness-of-fit on F^2	1.093
Final R indexes [I>=2σ (I)]	R_1 = 0.0471, wR_2 = 0.1290
Final R indexes [all data]	R_1 = 0.0548, wR_2 = 0.1448
Largest diff. peak/hole / e Å^{-3}	0.95/-0.54
Flack parameter	0.37(3)

Table S-19 Fractional Atomic Coordinates (×10^4) and Equivalent Isotropic Displacement Parameters (Å^2×10^3). U_{eq} is defined as 1/3 of the trace of the orthogonalised U_{IJ} tensor.

Atom	*x*	*y*	*z*	U(eq)
Br1	4434.6(8)	-2874.4(12)	7453.2(4)	95.1(4)
Br2	9168.0(7)	2514.0(10)	7261.9(4)	81.0(4)
N1	5563(3)	-97(4)	5368.3(17)	46.9(10)
N2	6603(3)	1244(4)	5299.1(18)	48.1(11)
C1	5974(4)	-52(6)	5802(2)	46.7(13)
C2	6623(4)	768(6)	5748(2)	46.8(13)
C3	5964(4)	713(5)	5083.4(19)	44.4(12)
C4	5644(4)	-746(6)	6204(2)	46.8(12)
C5	5642(4)	-1977(6)	6205(2)	49.9(13)
C6	5308(5)	-2615(6)	6573(3)	54(2)
C7	4947(5)	-2000(8)	6949(2)	60.1(16)
C8	4939(6)	-776(8)	6962(3)	76(2)
C9	5291(6)	-151(7)	6592(2)	67.5(19)
C10	7261(4)	1167(5)	6098(2)	47.3(13)
C11	7473(5)	456(7)	6484(3)	58.8(16)
C12	8044(5)	857(8)	6821(3)	65.6(18)
C13	8410(4)	1954(7)	6772(3)	57.8(16)
C14	8240(6)	2666(8)	6392(5)	66(2)
C15	7668(6)	2263(7)	6054(4)	62(2)
C16	4848(5)	-858(7)	5241(2)	62.7(18)
C17	5633(4)	958(6)	4605(2)	47.3(13)
C18	4990(4)	1830(6)	4551(2)	52.4(14)
C19	4654(7)	2484(6)	4945(4)	60(2)

Atom	*x*	*y*	*z*	U(eq)
C20	4021(6)	3326(7)	4874(4)	80(2)
C21	3725(6)	3582(8)	4415(4)	89(3)
C22	4014(6)	2989(8)	4043(4)	78(3)
C23	4648(5)	2078(7)	4092(3)	59.7(18)
C24	4941(5)	1393(8)	3712(3)	69(2)
C25	5567(5)	516(6)	3760(2)	58.8(16)
C26	5879(7)	-174(9)	3371(3)	85(3)
C27	6505(8)	-980(10)	3422(4)	97(3)
C28	6881(7)	-1177(8)	3871(3)	85(2)
C29	6582(5)	-544(7)	4253(3)	63.7(18)
C30	5936(4)	302(6)	4215(2)	51.1(13)
Br3	8063.6(10)	2297.6(14)	2541.9(4)	118.4(5)
Br4	3338.7(8)	7584.9(10)	2738.4(4)	84.3(4)
N3	6880(3)	4916(4)	4637.4(16)	46.2(10)
N4	5836(3)	6261(5)	4709.3(18)	48.7(11)
C31	6468(4)	4970(6)	4207(2)	44.9(12)
C32	5826(4)	5792(5)	4257(2)	44.2(12)
C33	6475(4)	5715(5)	4922.9(19)	44.9(12)
C34	6813(4)	4316(5)	3797.1(19)	44.6(12)
C35	6819(4)	3082(6)	3782(2)	53.4(14)
C36	7190(7)	2478(6)	3398(4)	61(3)
C37	7530(5)	3111(9)	3050(3)	68.1(19)
C38	7540(6)	4317(9)	3044(3)	75(2)
C39	7170(5)	4925(7)	3421(2)	64.3(18)
C40	5192(4)	6186(5)	3906(2)	46.3(12)
C41	4995(4)	5505(6)	3513(2)	58.2(16)
C42	4428(4)	5897(7)	3169(2)	59.3(16)
C43	4067(4)	7020(7)	3219(2)	57.4(16)
C44	4227(6)	7701(8)	3614(4)	64(2)
C45	4797(5)	7296(7)	3953(3)	58.1(19)
C46	7597(5)	4152(7)	4765(3)	65.0(19)
C47	6815(4)	5960(5)	5403(2)	44.8(12)
C48	7467(4)	6828(6)	5455(2)	48.8(13)
C49	7802(6)	7489(6)	5069(4)	58(2)
C50	8409(5)	8323(7)	5126(4)	77(2)
C51	8734(5)	8566(7)	5585(4)	79(2)
C52	8442(5)	7952(8)	5961(3)	69(2)
C53	7818(4)	7042(7)	5910(3)	55.1(15)
C54	7551(4)	6346(7)	6289(2)	57.5(16)
C55	6932(4)	5488(6)	6247(2)	55.9(15)
C56	6637(6)	4767(8)	6633(3)	73(2)

Atom	*x*	*y*	*z*	U(eq)
C57	5990(7)	3969(9)	6579(3)	81(2)
C58	5602(6)	3811(8)	6140(3)	78(2)
C59	5868(5)	4423(7)	5755(3)	62.4(17)
C60	6534(4)	5287(6)	5793(2)	49.4(13)

Table S-20 Selected Bond Distances (Å)

Atom	Atom	Length/Å	Atom	Atom	Length/Å
Br1	C7	1.907(7)	Br3	C37	1.896(8)
Br2	C13	1.928(6)	Br4	C43	1.883(7)
N1	C1	1.388(8)	N3	C31	1.379(8)
N1	C3	1.366(7)	N3	C33	1.361(7)
N1	C16	1.448(8)	N3	C46	1.452(8)
N2	C2	1.381(8)	N4	C32	1.388(7)
N2	C3	1.310(7)	N4	C33	1.315(7)
C1	C2	1.374(9)	C31	C32	1.366(8)
C1	C4	1.472(8)	C31	C34	1.476(8)
C2	C10	1.473(8)	C32	C40	1.469(8)
C3	C17	1.477(8)	C33	C47	1.487(8)
C4	C5	1.378(9)	C34	C35	1.382(9)
C4	C9	1.398(9)	C34	C39	1.382(9)
C5	C6	1.366(10)	C35	C36	1.406(12)
C6	C7	1.389(11)	C36	C37	1.327(14)
C7	C8	1.370(12)	C37	C38	1.349(13)
C8	C9	1.376(10)	C38	C39	1.393(10)
C10	C11	1.394(9)	C40	C41	1.384(9)
C10	C15	1.386(9)	C40	C45	1.393(9)
C11	C12	1.381(10)	C41	C42	1.386(9)
C12	C13	1.361(11)	C42	C43	1.384(11)
C13	C14	1.366(14)	C43	C44	1.377(13)
C14	C15	1.383(14)	C44	C45	1.384(12)
C17	C18	1.406(9)	C47	C48	1.412(9)
C17	C30	1.411(9)	C47	C60	1.408(9)
C18	C19	1.433(13)	C48	C49	1.419(13)
C18	C23	1.436(9)	C48	C53	1.424(9)
C19	C20	1.378(13)	C49	C50	1.338(12)
C20	C21	1.412(15)	C50	C51	1.423(14)
C21	C22	1.326(15)	C51	C52	1.347(14)
C22	C23	1.427(12)	C52	C53	1.415(11)

Atom	Atom	Length/Å	Atom	Atom	Length/Å
C23	C24	1.398(12)	C53	C54	1.391(11)
C24	C25	1.390(11)	C54	C55	1.366(10)
C25	C26	1.433(12)	C55	C56	1.437(10)
C25	C30	1.433(9)	C55	C60	1.445(8)
C26	C27	1.336(15)	C56	C57	1.355(13)
C27	C28	1.419(14)	C57	C58	1.393(13)
C28	C29	1.374(11)	C58	C59	1.354(11)
C29	C30	1.386(11)	C59	C60	1.422(10)

Table S-21 Selected Bond Angles

Atom	Atom	Atom	Angle/°	Atom	Atom	Atom	Angle/°
C1	N1	C16	126.7(5)	C31	N3	C46	127.1(5)
C3	N1	C1	106.8(5)	C33	N3	C31	106.4(5)
C3	N1	C16	126.5(5)	C33	N3	C46	126.5(5)
C3	N2	C2	105.8(5)	C33	N4	C32	105.1(5)
N1	C1	C4	120.5(5)	N3	C31	C34	120.4(5)
C2	C1	N1	105.4(5)	C32	C31	N3	106.3(5)
C2	C1	C4	134.0(6)	C32	C31	C34	132.9(6)
N2	C2	C10	121.3(6)	N4	C32	C40	121.5(5)
C1	C2	N2	110.1(5)	C31	C32	N4	109.9(5)
C1	C2	C10	128.6(6)	C31	C32	C40	128.7(5)
N1	C3	C17	120.4(5)	N3	C33	C47	120.1(5)
N2	C3	N1	111.9(5)	N4	C33	N3	112.4(5)
N2	C3	C17	127.6(5)	N4	C33	C47	127.3(5)
C5	C4	C1	122.0(6)	C35	C34	C31	121.5(6)
C5	C4	C9	118.2(6)	C35	C34	C39	117.8(6)
C9	C4	C1	119.7(6)	C39	C34	C31	120.7(6)
C6	C5	C4	121.7(6)	C34	C35	C36	120.5(7)
C5	C6	C7	118.8(7)	C37	C36	C35	119.0(7)
C6	C7	Br1	119.4(6)	C36	C37	Br3	119.0(7)
C8	C7	Br1	119.3(6)	C36	C37	C38	123.2(7)
C8	C7	C6	121.3(7)	C38	C37	Br3	117.8(7)
C7	C8	C9	118.9(7)	C37	C38	C39	118.3(7)
C8	C9	C4	121.0(7)	C34	C39	C38	121.2(7)
C11	C10	C2	121.1(6)	C41	C40	C32	121.9(5)
C15	C10	C2	121.1(6)	C41	C40	C45	118.0(6)
C15	C10	C11	117.8(6)	C45	C40	C32	120.0(6)

Atom	Atom	Atom	Angle/°	Atom	Atom	Atom	Angle/°
C12	C11	C10	120.7(7)	C40	C41	C42	122.2(6)
C13	C12	C11	119.5(7)	C43	C42	C41	118.3(6)
C12	C13	Br2	118.3(6)	C42	C43	Br4	118.4(5)
C12	C13	C14	121.7(7)	C44	C43	Br4	120.8(6)
C14	C13	Br2	119.9(6)	C44	C43	C42	120.8(7)
C13	C14	C15	118.7(7)	C43	C44	C45	120.0(7)
C14	C15	C10	121.4(8)	C44	C45	C40	120.5(8)
C18	C17	C3	118.6(6)	C48	C47	C33	118.5(5)
C18	C17	C30	120.9(6)	C60	C47	C33	120.7(5)
C30	C17	C3	120.4(6)	C60	C47	C48	120.7(5)
C17	C18	C19	121.9(7)	C47	C48	C49	122.9(6)
C17	C18	C23	119.8(7)	C47	C48	C53	119.0(6)
C19	C18	C23	118.2(7)	C49	C48	C53	118.1(7)
C20	C19	C18	119.8(10)	C50	C49	C48	122.0(10)
C19	C20	C21	120.4(10)	C49	C50	C51	119.7(9)
C22	C21	C20	121.5(8)	C52	C51	C50	120.4(7)
C21	C22	C23	121.0(9)	C51	C52	C53	121.3(8)
C22	C23	C18	118.9(8)	C52	C53	C48	118.5(8)
C24	C23	C18	118.2(7)	C54	C53	C48	119.5(6)
C24	C23	C22	122.9(8)	C54	C53	C52	122.0(7)
C25	C24	C23	122.7(6)	C55	C54	C53	122.4(6)
C24	C25	C26	122.9(7)	C54	C55	C56	123.5(6)
C24	C25	C30	119.2(7)	C54	C55	C60	119.4(6)
C30	C25	C26	117.9(7)	C56	C55	C60	117.1(7)
C27	C26	C25	121.8(8)	C57	C56	C55	121.3(7)
C26	C27	C28	120.3(9)	C56	C57	C58	120.6(8)
C29	C28	C27	119.2(9)	C59	C58	C57	121.5(8)
C28	C29	C30	122.4(8)	C58	C59	C60	120.4(8)
C17	C30	C25	119.0(6)	C47	C60	C55	118.9(6)
C29	C30	C17	122.6(6)	C47	C60	C59	122.1(6)
C29	C30	C25	118.4(6)	C59	C60	C55	119.0(6)

Table S-22 Selected Torsion Angles

A	B	C	D	Angle/°	A	B	C	D	Angle/°
Br1	C7	C8	C9	-178.4(7)	Br3	C37	C38	C39	-178.3(6)
Br2	C13	C14	C15	-177.4(7)	Br4	C43	C44	C45	-176.8(7)
N1	C1	C2	N2	1.6(7)	N3	C31	C32	N4	0.6(7)
N1	C1	C2	C10	179.9(6)	N3	C31	C32	C40	-179.8(6)

A	B	C	D	Angle/°	A	B	C	D	Angle/°
N1	C1	C4	C5	65.5(9)	N3	C31	C34	C35	67.2(9)
N1	C1	C4	C9	-112.1(8)	N3	C31	C34	C39	-110.3(7)
N1	C3	C17	C18	86.7(7)	N3	C33	C47	C48	85.6(7)
N1	C3	C17	C30	-91.3(7)	N3	C33	C47	C60	-90.4(7)
N2	C2	C10	C11	-158.9(6)	N4	C32	C40	C41	-159.8(6)
N2	C2	C10	C15	21.4(10)	N4	C32	C40	C45	22.8(9)
N2	C3	C17	C18	-88.7(8)	N4	C33	C47	C48	-88.6(8)
N2	C3	C17	C30	93.3(8)	N4	C33	C47	C60	95.4(8)
C1	N1	C3	N2	1.2(7)	C31	N3	C33	N4	0.6(7)
C1	N1	C3	C17	-174.9(6)	C31	N3	C33	C47	-174.4(6)
C1	C2	C10	C11	22.9(10)	C31	C32	C40	C41	20.6(10)
C1	C2	C10	C15	-156.8(8)	C31	C32	C40	C45	-156.8(7)
C1	C4	C5	C6	-177.7(7)	C31	C34	C35	C36	-176.8(7)
C1	C4	C9	C8	176.8(7)	C31	C34	C39	C38	176.4(7)
C2	N2	C3	N1	-0.2(7)	C32	N4	C33	N3	-0.2(7)
C2	N2	C3	C17	175.5(6)	C32	N4	C33	C47	174.4(6)
C2	C1	C4	C5	-119.6(9)	C32	C31	C34	C35	-121.4(8)
C2	C1	C4	C9	62.8(11)	C32	C31	C34	C39	61.0(11)
C2	C10	C11	C12	-177.3(7)	C32	C40	C41	C42	-176.9(7)
C2	C10	C15	C14	177.2(8)	C32	C40	C45	C44	177.2(8)
C3	N1	C1	C2	-1.7(7)	C33	N3	C31	C32	-0.7(7)
C3	N1	C1	C4	174.5(6)	C33	N3	C31	C34	172.7(6)
C3	N2	C2	C1	-0.9(7)	C33	N4	C32	C31	-0.3(7)
C3	N2	C2	C10	-179.4(6)	C33	N4	C32	C40	-179.9(5)
C3	C17	C18	C19	0.2(9)	C33	C47	C48	C49	0.9(9)
C3	C17	C18	C23	-179.3(6)	C33	C47	C48	C53	-177.9(5)
C3	C17	C30	C25	176.5(6)	C33	C47	C60	C55	175.0(5)
C3	C17	C30	C29	-3.6(10)	C33	C47	C60	C59	-5.3(9)
C4	C1	C2	N2	-173.8(7)	C34	C31	C32	N4	-171.6(7)
C4	C1	C2	C10	4.5(12)	C34	C31	C32	C40	8.0(12)
C4	C5	C6	C7	1.2(12)	C34	C35	C36	C37	-0.3(12)
C5	C4	C9	C8	-0.9(12)	C35	C34	C39	C38	-1.2(11)
C5	C6	C7	Br1	177.4(6)	C35	C36	C37	Br3	177.9(6)
C5	C6	C7	C8	-1.5(13)	C35	C36	C37	C38	0.1(14)
C6	C7	C8	C9	0.5(13)	C36	C37	C38	C39	-0.5(13)
C7	C8	C9	C4	0.7(13)	C37	C38	C39	C34	1.1(12)
C9	C4	C5	C6	-0.1(11)	C39	C34	C35	C36	0.8(10)
C10	C11	C12	C13	-0.7(12)	C40	C41	C42	C43	1.5(12)
C11	C10	C15	C14	-2.5(13)	C41	C40	C45	C44	-0.3(12)
C11	C12	C13	Br2	177.4(6)	C41	C42	C43	Br4	177.1(6)
C11	C12	C13	C14	-1.2(12)	C41	C42	C43	C44	-3.7(12)

A	B	C	D	Angle/°	A	B	C	D	Angle/°
C12	C13	C14	C15	1.2(14)	C42	C43	C44	C45	4.0(14)
C13	C14	C15	C10	0.7(15)	C43	C44	C45	C40	-1.9(14)
C15	C10	C11	C12	2.5(11)	C45	C40	C41	C42	0.5(11)
C16	N1	C1	C2	178.0(6)	C46	N3	C31	C32	178.6(6)
C16	N1	C1	C4	-5.8(10)	C46	N3	C31	C34	-8.0(10)
C16	N1	C3	N2	-178.5(6)	C46	N3	C33	N4	-178.7(6)
C16	N1	C3	C17	5.4(10)	C46	N3	C33	C47	6.2(10)
C17	C18	C19	C20	-179.6(7)	C47	C48	C49	C50	178.8(7)
C17	C18	C23	C22	-178.3(6)	C47	C48	C53	C52	-177.2(6)
C17	C18	C23	C24	3.1(9)	C47	C48	C53	C54	3.8(9)
C18	C17	C30	C25	-1.4(9)	C48	C47	C60	C55	-0.9(8)
C18	C17	C30	C29	178.5(6)	C48	C47	C60	C59	178.7(6)
C18	C19	C20	C21	-2.5(13)	C48	C49	C50	C51	-0.1(13)
C18	C23	C24	C25	-2.3(10)	C48	C53	C54	C55	-2.9(10)
C19	C18	C23	C22	2.2(10)	C49	C48	C53	C52	3.9(9)
C19	C18	C23	C24	-176.4(7)	C49	C48	C53	C54	-175.1(6)
C19	C20	C21	C22	3.1(14)	C49	C50	C51	C52	0.8(13)
C20	C21	C22	C23	-0.9(14)	C50	C51	C52	C53	0.9(13)
C21	C22	C23	C18	-1.7(12)	C51	C52	C53	C48	-3.3(11)
C21	C22	C23	C24	176.8(8)	C51	C52	C53	C54	175.7(7)
C22	C23	C24	C25	179.2(7)	C52	C53	C54	C55	178.2(7)
C23	C18	C19	C20	-0.1(11)	C53	C48	C49	C50	-2.3(11)
C23	C24	C25	C26	-178.8(7)	C53	C54	C55	C56	-179.2(7)
C23	C24	C25	C30	-0.4(11)	C53	C54	C55	C60	0.0(10)
C24	C25	C26	C27	177.4(9)	C54	C55	C56	C57	176.6(8)
C24	C25	C30	C17	2.3(9)	C54	C55	C60	C47	1.9(9)
C24	C25	C30	C29	-177.6(7)	C54	C55	C60	C59	-177.7(6)
C25	C26	C27	C28	0.0(16)	C55	C56	C57	C58	1.2(14)
C26	C25	C30	C17	-179.2(6)	C56	C55	C60	C47	-178.8(6)
C26	C25	C30	C29	0.9(10)	C56	C55	C60	C59	1.5(9)
C26	C27	C28	C29	1.2(16)	C56	C57	C58	C59	1.5(15)
C27	C28	C29	C30	-1.3(15)	C57	C58	C59	C60	-2.6(14)
C28	C29	C30	C17	-179.6(8)	C58	C59	C60	C47	-178.6(7)
C28	C29	C30	C25	0.3(12)	C58	C59	C60	C55	1.0(11)
C30	C17	C18	C19	178.2(6)	C60	C47	C48	C49	176.9(6)
C30	C17	C18	C23	-1.3(9)	C60	C47	C48	C53	-1.9(8)
C30	C25	C26	C27	-1.1(13)	C60	C55	C56	C57	-2.7(11)
